# A new balaenopterid whale from the late Miocene of the Southern North Sea Basin and the evolution of balaenopterid diversity (Cetacea, Mysticeti)

**DOI:** 10.7717/peerj.6915

**Published:** 2019-05-17

**Authors:** Michelangelo Bisconti, Dirk K. Munsterman, Klaas Post

**Affiliations:** 1 Paleobiology Department, San Diego Natural History Museum, San Diego, CA, USA; 2 Dipartimento di Scienze della Terra, Università degli Studi di Torino, Torino, Italia; 3 Toegepast Natuurwetenschappelijk Onderzoek (TNO-Netherlands Organization for Applied Scientific Research), Geological Survey of The Netherlands, Utrecht, The Netherlands; 4 Het Natuurhistorisch Museum, Rotterdam, The Netherlands

**Keywords:** Anatomy, Balaenopteridae, Cetacea, Dynocists, Miocene, Mysticeti, Skull, Phylogeny, North Sea, Evolutionary radiations

## Abstract

**Background:**

Balaenopterid mysticetes represent the most successful family-rank group of this clade. Their evolutionary history is characterized by a rich fossil record but the origin of the living genera is still largely not understood. Recent discoveries in the southern border of the North Sea revealed a number of well preserved fossil balaenopterid whales that may help resolving this problem. In particular, skull NMR 14035 shares morphological characters with the living humpback whale, *Megaptera novaeangliae* and, for this reason, its characteristics are investigated here.

**Methods:**

The comparative anatomical analysis of the new specimen formed the basis of a new phylogenetic analysis of the Mysticeti based on a matrix including 350 morphological character states scored for 82 Operational Taxonomic Units. The stratigraphic age of the specimen was determined based on the analysis of the dinocyst assemblage recovered in the associated sediment. We assessed clade diversity in Balaenopteridae by counting the numbers of clades in given time intervals and then plotted the results.

**Results:**

*Nehalaennia devossi* n. gen. et sp. is described for the first time from the late Tortonian (8.7–8.1 Ma) of the Westerschelde (The Netherlands). This new taxon belongs to Balaenopteridae and shows a surprisingly high number of advanced characters in the skull morphology. *Nehalaennia devossi* is compared to a large sample of balaenopterid mysticetes and a phylogenetic analysis placed it as the sister group of a clade including the genus *Archaebalaenoptera*. The inclusion of this fossil allowed to propose a phylogenetic hypothesis for Balaenopteridae in which (1) Eschrichtiidae (gray whales) represents a family of its own, (2) Balaenopteridae + Eschrichtiidae form a monophyletic group (superfamily Balaenopteroidea), (3) Cetotheriidae is the sister group of Balaenopteroidea, (4) living *Balaenoptera* species form a monophyletic group and (5) living *M. novaeangliae* is the sister group of *Balaenoptera*. Our work reveals a complex phylogenetic history of Balaenopteridae and *N. devossi* informs us about the early morphological transformations in this family. Over time, this family experienced a number of diversity pulses suggesting that true evolutionary radiations had taken place. The paleoecological drivers of these pulses are then investigated.

## Introduction

Rorquals and humpbacks belong to the family Balaenopteridae, the most successful group among living baleen whales. They include the largest animal ever lived on our Earth (the blue whale, *Balaenoptera musculus*) and filter feed on krill and small fishes. The biology and evolution of balaenopterid whales is the focus of numerous research projects carried out by different research teams all around the world. One of the most investigated questions, and maybe the most debated one, is about deciphering their origin and evolution as to understand how and when they attained their gigantic body size and the sophisticated biomechanical characteristics allowing them to exploit large masses of prey (Goldbogen et al., 2017; Slater, Goldbogen & Pyenson, 2017; Berta et al., 2016).

Different approaches have been followed to develop a hypothesis of phylogeny for balaenopterid whales resulting in conflicting results. As detailed in the Discussion section, morphology-based, molecule-based and total evidence analyses failed in finding a consensus about (1) the phylogenetic relationships of Balaenopteridae and other mysticetes and (2) the intra-family relationships of Balaenopteridae. The discrepancy is especially evident when molecule-based and morphology-based results are compared in that molecule-based do not support (1) the position of gray whales with respect to balaenopterid whales, (2) the monophyly of Balaenopteridae, (3) the monophyly of *Balaenoptera*, (4) the assignment of the humpback whale to its own genus (i.e., *Megaptera*) (see Arnason et al. (2018) and literature therein for a summary of molecular results). Also total evidence analyses got similar results challenging the ordered branching pattern obtained by early and recent traditional, morphology-based works (compare, e.g., Marx & Fordyce, 2016 and Slater, Goldbogen & Pyenson, 2017 vs. Deméré, Berta & McGowen, 2005 and Bisconti & Bosselaers, 2016).

One of the key differences between molecule-based and morphology-based works consists in that the fossil record may be included only in the latter ones or in total evidence studies. This is a major point to consider since the fossil record of Balaenopteridae is vast. It is a matter of fact that most of the balaenopterid species are now extinct and the extant taxa only represent a small fraction of the past diversity of this family (Berta et al., 2016). The fossil record of Balaenopteridae extends far back to the Tortonian (between *c.* 11.6 and *c.* 7.2 Ma); balaenopterids, then, experienced a burst in diversity during the Pliocene (between *c.* 5.3 and *c.* 2.5 Ma). Earliest balaenopterids show a number of primitive characters related to intermittent ram feeding mechanisms that were not as advanced as those observed in the living species (Berta et al., 2016; Bisconti, 2007, 2010a, 2010b, 2011); observations on the osteological structures in the dentary, the temporal fossa, the frontal and the craniomandibular joint suggest that advanced characters were developed gradually (that is, not abruptly) during the evolution of the family (Berta et al., 2016). The fossil record informs us, thus, not only about the past taxonomic diversity of the family but also about the tempo of the morphological transformations leading to the optimized biomechanics of the living rorquals and humpbacks. An interesting example of this kind of information came from the recent description of *Fragilicetus velponi* (Bisconti & Bosselaers, 2016) which preserves a number of archaic, cetotheriid- and eschrichtiid-like characters in the skull showing how a gradual process of character acquisition and loss could lead to the architecture of modern balaenopterid species.

**Figure 1:**
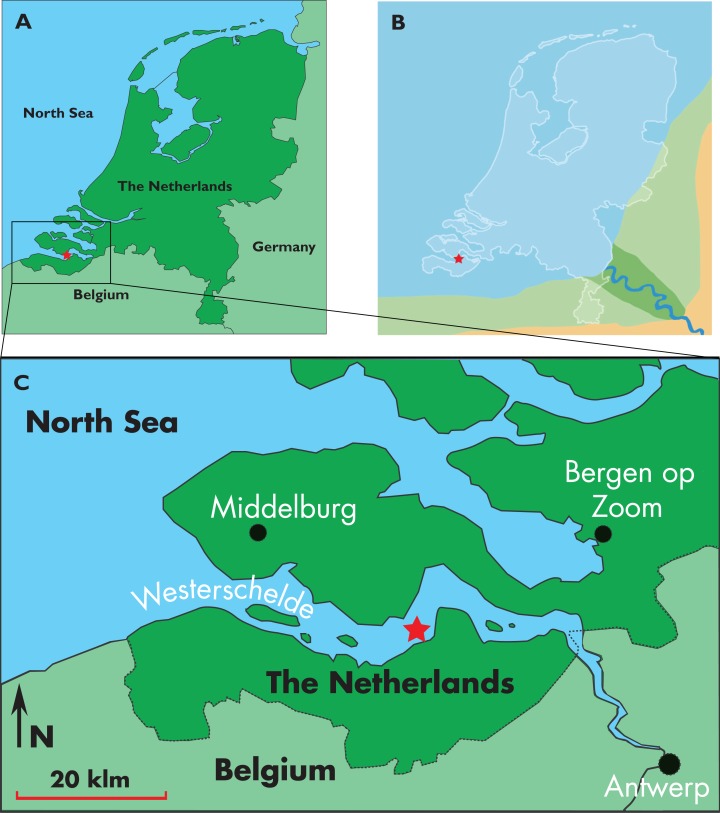
Type locality of *Nehalaennia devossi*. Map showing the location of the discovery of the holotype skull of *Nehalaennia devossi* (NMR 999100014035). (A) Type locality in the geography of Holland. (B) Close view of the type locality in the Westerschelde. (C) Reconstruction of the late Miocene paleogeography of The Netherlands. In all the illustrations, the star corresponds to the type locality.

Here, we describe a new balaenopterid genus and species, which is based on the discovery of a partial skull from the Westerschelde, The Netherlands (Fig. 1). The skull is one of the oldest balaenopterid fossils as it is dated to *c.* 8.7–8.1 Ma based on dinoflagellate cysts but, surprisingly, shows a number of synapomorphies of living balaenopterids. As it is beautifully preserved, it represents an invaluable source of character states helping the reconstruction of the early evolution of the balaenopterid whales. The discovery of this new taxon has the potential to resolve the questions related to the monophyly of *Balaenoptera* and *Megaptera*, and those related to the inclusion of the gray whale within Balaenopteridae or not.

The new skull is described and compared against a vast sample of living and fossil balaenopterid whales, and is used in a new morphology-based phylogenetic analysis. The new phylogenetic study includes as far as 350 characters scored for 82 taxa including 35 balaenopterid Operational Taxonomic Units (hereinafter: OTUs) being the most inclusive phylogenetic investigation into the phylogenetic relationships of this diverse and interesting family up to now.

Our new phylogenetic analysis represents a tool to describe eventual episodes of evolutionary radiations occurring in the clade including the crown mysticetes (i.e., Balaenomorpha Geisler & Sanders, 2003). Therefore, apart from resolving the phylogenetic relationships of the new taxon, we also performed a study of the past diversity within Balaenomorpha focusing on the whole clade and on Balaenopteridae and compared our results with those provided by works of other authors.

## Materials and Methods

### Institutional abbreviations

NHML, Natural History Museum, Lima, Peru. NMR, Natuurhistorisch Museum Rotterdam, The Netherlands. RBINS, Royal Belgian Institute of Natural Sciences, Brussels, Belgium.

### Terminology

Anatomical terminology is based on Mead & Fordyce (2009) and, when necessary, Ekdale, Berta & Deméré (2011) and Kellogg (1965, 1968).

### Studied specimen

The rorqual specimen coded NMR 999100014035 (holotype) was taken from the bed of the Westerschelde estuary by NMR expedition 2014/3, at a depth of 30 meters, at position *c.* 51°21′N, 03°54′E. (locality 6D, Post & Reumer, 2016) (Fig. 1). Preparation of the specimen was done with mechanical tools by one of the authors (KP).

### Comparative analysis

The skull NMR 999100014035 is compared with an extended record of living and fossil Balaenopteridae. In particular, the writers obtained first-hand data by the direct study of specimens of all the living balaenopterid species (with the exclusion of *B. omurai*) and the direct study of the type and associated materials belonging to *Archaebalaenoptera castriarquati*, *Plesiobalaenoptera quarantellii*, *‘Megaptera’ hubachi*, *Fragilicetus velponi*, *‘Balaenoptera’ cortesii* var. *portisi*, *Diunatans luctoretemergo*, a second and still undescribed specimen of *Incakujira anillodefuego* (i.e., NHML 1613), and undescribed balaenopterid specimens from Italy (MPTAM 207.13307, UT PU13842/5), Belgium (RBINS M. 2231 and M. 2315), The Netherlands (NMR 9991-00007096; hereinafter: NMR 7096) and Peru (MHNL 1610) cited and briefly mentioned in Bisconti & Bosselaers (2016). Non mentioned specimens are outlined in the Supplemental Information. Additional comparisons to mysticete species belonging to different families were carried out based on specimens cited in Bisconti (2011, 2012, 2015), Bisconti, Lambert & Bosselaers (2013) and Bisconti & Bosselaers (2016) (see Supplemental Information for the complete list).

### Nomenclatural act

The electronic version of this article in Portable Document Format will represent a published work according to the International Commission on Zoological Nomenclature (ICZN), and hence the new names contained in the electronic version are effectively published under that Code from the electronic edition alone. This published work and the nomenclatural acts it contains have been registered in ZooBank, the online registration system for the ICZN. The ZooBank LSIDs (Life Science Identifiers) can be resolved and the associated information viewed through any standard web browser by appending the LSID to the prefix http://zoobank.org/. Publication LSID is urn:lsid:zoobank.org:pub:EB25A914-5D9E-4838-9C10-AAB9897E2B51. The online version of this work is archived and available from the following digital repositories: PeerJ, PubMed Central and CLOCKSS.

### Phylogenetic analysis

The phylogenetic analysis was carried out based on a morphological dataset formed by 347 characters from osteology and three characters from baleen morphology. Character states were scored for 82 OTUs representing most of the mysticete radiations. Balaenopteridae are represented by 35 OTUs making this analysis the most detailed investigation into the phylogenetic relationships within this family. Both the list of character states and the taxon x character matrix are presented in the Supplemental Information together with the relevant literature used for character definitions and distribution, and species morphologies. The matrix was analyzed through TNT 1.5 (Goloboff & Catalano, 2016). We used the traditional search algorithm (hereinafter: TS) and the new technology search (hereinafter: NT) in the search for the most parsimonious solution(s). In the NT search, we followed two procedures: (1) we enabled the Sectorial Search and run three separate analyses by using alternatively Tree Fusing, Drift and Ratchet algorithms; (2) we disabled the Sectorial Search and run a single analysis with all the Tree Fusing, Drift and Ratchet algorithms. Both the TS and the NT searches were done with unweighted and weighted character states. The procedures with weighted character states were done by using default options in TNT 1.5. Calculus of consistency index, retention index, and homoplasy index were done through dedicated applications of TNT. A bootstrap analysis with 1,000 replicates and a symmetric resampling analysis with 100 replicates (33 change probability and output result as frequency differences) were performed to quantify the morphological support to nodes by using TNT.

The calculus of the Stratigraphic Consistency Index (Huelsenbeck, 1994) was performed to assess the degree of agreement between the stratigraphic ages of the OTUs and the branching pattern of the resulting cladograms. To this scope, a list of stratigraphic ages of the OTUs is presented in the Supplemental Information together with the relevant literature. Most of the stratigraphic information is from the Cetacea partition of the Paleobiology Database (http://www.fossilworks.org/) that was mainly compiled by Mark Uhen. Additional information is from dedicated literature cited in the Supplemental Information. The formula for the calculous of the SCI is the following:

SCI = (number of stratigraphically consistent nodes)/(total number of nodes) (Huelsenbeck, 1994).

Morphological transformations at selected nodes were reconstructed by the appropriate commands of Mesquite 3.51 (Maddison & Maddison, 2009). The reconstruction of character history made by Mesquite 3.51 used both a parsimony model and a probabilistic method using the Mk 1 model for maximum likelihood calculation.

### Testing the evolutionary radiation hypothesis

The evolutionary radiation is the dramatic proliferation of taxa in a clade (Simões et al., 2016 and literature therein). To see if evolutionary radiation episodes can be detected in the fossil record of the clade including crown mysticetes (Balaenomorpha), we counted presence of taxa in the last 40 Ma and plotted the numbers in a graph against the temporal scale expressed in million years. We counted not only presence of taxa based on their occurrence in the fossil record but also the inferred presence of taxa in given time intervals based on the phylogenetic results, for this reason, we counted all the rami predicted by the cladogram to occur in given time intervals. We made separate analyses: (1) Balaenomorpha as a whole, (2) Balaenomorpha without Balaenopteridae, (3) Balaenopteridae only and (4) Balaenoidea (data from Bisconti, Lambert & Bosselaers, 2017). The resulting graphs are then used to infer eventual episodes of evolutionary radiations.

### Palynological preparation and analysis

**Figure 2:**
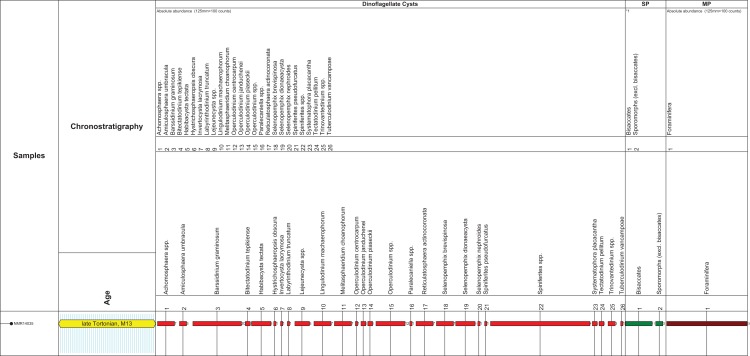
Stratigraphic markers associated to the holotype skull of *Nehalaennia devossi* (NMR 999100014035). Dinocyst stratigraphic markers and age assessment of sediment found inside the holotype skull of *Nehalaennia devossi* (NMR 14035). Zonation based on Munsterman & Brinkhuis (2004) recalibrated to Ogg, Ogg & Gradstein (2016). Abbreviations: MP, miscellaneous palynomorphs; SP, sporomorphs (spores and pollen).

Cemented fine sands (the matrix) attached to the present fossil rorqual cranium were prepared at Palynological Laboratory Services (hereinafter: PLS) located in UK, using the standard sample processing procedures, which involves HCl and HF treatment, heavy liquid separation, and sieving over a 15 µm mesh sieve. The organic residue was mounted with glycerin-gelatin on microscopic slides. Two microscopic slides were made: in addition to a non-oxidized kerogen slide, the organic residue was, also slightly oxidized with HNO_3_ in order to concentrate the palynomorphs and reduce the abundant ‘Structureless Organic Matter’. The palynological analysis was carried out at the Geological Survey of the Netherlands (TNO) according to standard procedures. The palynomorph association on the microscope slides was counted until approximately a total of 200 sporomorphs (pollen and spores) and marine dinoflagellate cysts was reached. The main miscellaneous categories (e.g., marine acritarchs, test linings of foraminifers and brackish water algae *Botryococcus*) were calculated separately. The remainder of the slide was thereafter scanned for any (rarer) dinocyst species. Diagnostic species are discussed in the dedicate chapter, a complete distribution chart including all species found is given in Fig. 2. The age interpretation is based on the last occurrence datum (LOD) and first occurrence datum of dinoflagellate cysts. For the dinoflagellate cyst taxonomy the so-called ‘Lentin and Williams index’ is followed (Williams, Fensome & MacRae, 2017). Palynological interpretation is based on key-references concerning the palynostratigraphy of the Neogene from the North Sea region such as: Dybkjær & Piasecki (2010), Köthe (2012), Kuhlmann et al. (2006), Louwye, Head & De Schepper (2004), Louwye & De Schepper (2010), Munsterman & Brinkhuis (2004) and Powell (1992). The Geological Time Scale 2016 is used (Ogg, Ogg & Gradstein, 2016). For the dinozones is referred to Munsterman & Brinkhuis (2004) recalibrated to Ogg, Ogg & Gradstein (2016) (Fig. 2).

## Systematic Paleontology

Mammalia Linneaus, 1758

Cetartiodactyla Montgelard, Caatzeflis & Douzery, 1997

Cetacea Brisson, 1762

Neoceti Fordyce & De Muizon, 2001

Mysticeti Flower, 1864

Chaeomysticeti Mitchell, 1989

Thalassotherii Bisconti, Lambert & Bosselaers, 2013

Balaenopteridae Gray, 1864

*Nehalaennia* new genus

### Diagnosis of genus

As for the only known species of this genus.

*Nehalaennia devossi* new species

### Holotype

Specimen 999100014035 of the collection of the Natuurhistorisch Museum Rotterdam.

### Repository

Natuurhistorisch Museum Rotterdam, Rotterdam, Holland.

### Etymology

The genus name is one of the spellings of the name of the Keltic pagan goddess of the sea which was also accepted by Romans when they conquered what is now the most southern province of The Netherlands. The species name is given to honor Dr. John de Vos for his lifelong contribution to Dutch paleontology and his leading role in creating the unique bond and trust between Dutch professional and amateur paleontologists.

### Diagnosis of species

Differential diagnosis: *Nehalaennia devossi* differs from *Archaebalaenoptera castriarquati* in having a rounded anterior border of the supraoccipital, anterior half of the supraoccipital not strongly compressed transversely, widely concave posterior border of the maxilla, shorter and wider ascending process of the maxilla, significantly shorter nasal bones and anterior border of the supraorbital process of the frontal anterolaterally concave. It differs from *Plesiobalaenoptera quarantellii* in showing a lower superior portion of the periotic, shorter and wider ascending process of the maxilla, more slender lateral process of the maxilla with deeper antorbital notch, posterior end of the posterior process of the periotic more robust and round. It differs from *‘Megaptera’ hubachi* in having a ventrally concave glenoid fossa of the squamosal with the postglenoid process projecting ventrally and forming a *c.* 90° angle with the zygomatic process of the squamosal, in having a rounded anterior border of the supraoccipital, and in lacking exposure of the alisphenoid in the temporal fossa. It differs from *‘Balaenoptera’ bertae* in having a wider and rounder anterior border of the supraoccipital, in having an anterolaterally concave anterior border of the supraorbital process of the frontal, in having a vertically-oriented postglenoid process of the squamosal making the glenoid fossa of the squamosal more concave in lateral view. It differs from *Incakujira anillodefuego* in having a rounder and wider anterior border of the supraoccipital, in having a comparatively shorter and slender supraorbital process of the frontal and a comparatively shorter zygomatic process of the squamosal, in having the premaxilla terminating anteriorly to the nasal. It differs from *‘Megaptera’ miocaena* in having a narrower anterior border of the supraoccipital, comparatively longer ascending process of the maxilla with ‘primary dorsal infraorbital foramina’, more concave glenoid fossa of the squamosal. It differs from *Fragilicetus velponi* in lacking a squamosal bulging into the temporal fossa, in having a wider anterior border of the supraoccipital, in having a less strongly protruding posterolateral corner of the exoccipital, in having a rounded dorsal border of the periotic. It differs from *Protororqualus cuvieri* in having a wider and rounder anterior border of the supraoccipital, in having shorter zygomatic process of the squamosal, in having a wider space between the posterior border of the maxilla and the anterior border of the supraorbital process of the frontal, and in having an anterolaterally concave anterior border of the supraorbital process of the frontal. The same differences are observed when *Nehalaennia devossi* is compared against *‘Balaenoptera’ cortesi* var. *portisi*. It differs from *Parabalaenoptera baulinensis* in having shorter and wider ascending process of the maxilla, rounded supraoccipital and shorter nasal bones.

*Nehalaennia devossi* differs from the genus *Balaenoptera* in having a rounded anterior border of the supraoccipital, rounded posterior end of the ascending process of the maxilla, anterolaterally concave anterior border of the supraorbital process of the frontal, alisphenoid not exposed in the temporal fossa. It differs from *Megaptera novaeangliae* in having zygomatic process of the squamosal less diverging from the longitudinal axis of the skull, anterior border of the pars cochlearis of the periotic not strongly protruded, and more concave glenoid fossa of the squamosal in lateral view.

### Horizon and locality

Cranium NMR 999100014035 (holotype of *N. devossi*) was embedded in a dense sandy glauconitic matrix originating from the Breda Formation, widespread at the site, which includes late Burdigalian to Messinian strata. The Breda Formation was deposited in a predominantly restricted-to open marine environment. The formation largely consists of fore-set and bottom-set beds deposited in a delta-front setting. Along the edges of the distribution area (near) coastal settings occur. Dinoflagellate cysts determined the matrix to be of late Tortonian age 8.7–8.1 Ma. From the same site and the same lithological unit, articulated fossils of an unidentified odontocete, several ziphiids, two balaenopterids different from the holotype of *N. devossi*, two cetotheres, and a basking shark were recovered and dated to the same geological period (Post & Reumer, 2016).

## Palynofacies and Age Assessment

The microflora is dominated by marine dinoflagellate cysts (92% of the total sum palynomorphs; Fig. 2). Only a few sporomorphs are present (8% of the total sum dinoflagellate cysts and sporomorphs). Most of the sporomorphs are bisaccate pollen (79% of the total sum sporomorphs). Bisaccate pollen are formed by conifers, gymnosperms (*Gymnospermae*). Bisaccate pollen have a higher aerial and aquatic buoyancy than other sporomorphs, indicating a relatively distal position from the coast. The relatively distal facies is confirmed by the concentration and composition of marine dinoflagellate cysts. The most common genus *Spiniferites* (27% of the total dinocyst sum) has a preferential orientation for open marine conditions. *Barssidinium graminosum* on the contrary, is also well-represented (13% of the total sum dinoflagellate cysts). This taxon has a temperate to tropical distribution in neritic and especially inner neritic waters. In addition, the coastal water taxon *Lingulodinium machaerophorum* is also present (5%) in the assemblage. Striking is the high number of heterotrophic genera like *Barssidinium, Lejeunecysta* and *Selenopemphix.* The heterotrophic dinoflagellate cysts refer to nutrient-rich water. As a whole, the marine dinocyst assemblage is relatively variegated indicating nutrient-rich neritic conditions.

Age diagnostic taxa are *Hystrichosphaeropsis obscura* and *Labyrinthodinium truncatum*. These taxa have a LOD in the late Miocene, late Tortonian, Zone SNSM14 (Munsterman & Brinkhuis, 2004). These events are also used to define the DN9 Zone by de Verteuil & Norris (1996) (east coast USA and generally adopted in Belgium), in Germany by Köthe (2012) and for the *Hystrichosphaeropsis obscura* Zone on- and offshore Denmark by Dybkjær & Piasecki (2010). In addition the dinocyst *Systematophora placacantha* is present. This taxon has a slightly older LOD in the late Miocene, late Tortonian, Zone SNSM13 (Munsterman & Brinkhuis, 2004). Zone SNSM13 comprises the lower part of the DN 9 Zone defined by de Verteuil & Norris (1996) (east coast USA and generally adopted in Belgium), in Germany by Köthe (2012) and for the *Hystrichosphaeropsis obscura* Zone on- and offshore Denmark by Dybkjær & Piasecki (2010). Marker taxa indicating possible older zones, like for example, *Palaeocystodinum golzowensis* (LOD in Zone SNSM12) are missing. The presence of *Operculodinium janduchenei* with an maximum age range in the Tortonian confirms the dating. In conclusion the age assessment of the present assemblage is late Miocene, late Tortonian SNSM13 Zone, ca. 8.7–8.1 Ma (Munsterman & Brinkhuis, 2004, recalibrated to the GTS of Ogg, Ogg & Gradstein, 2016).

## Description

### Overview and preservation

The specimen includes a well preserved skull with periotics in articulation. The rostrum is truncated anteriorly at about three-fourths of the narial fossa. The right zygomatic process, both premaxillae and both tympanic bullae are missing due to postmortem damage. A complex fracture is observed on the left side of the skull posteriorly to the supraorbital process of the frontal making it difficult to understand the sutural pattern among parietal, pterygoid, frontal and alisphenoid. A small amount of deformation is also observed as the position of the left maxilla is slightly more lateral than expected with respect to the longitudinal axis of the skull suggesting that an initial stage of rostral disarticulation was occurring just before the burial of the specimen.

**Figure 3:**
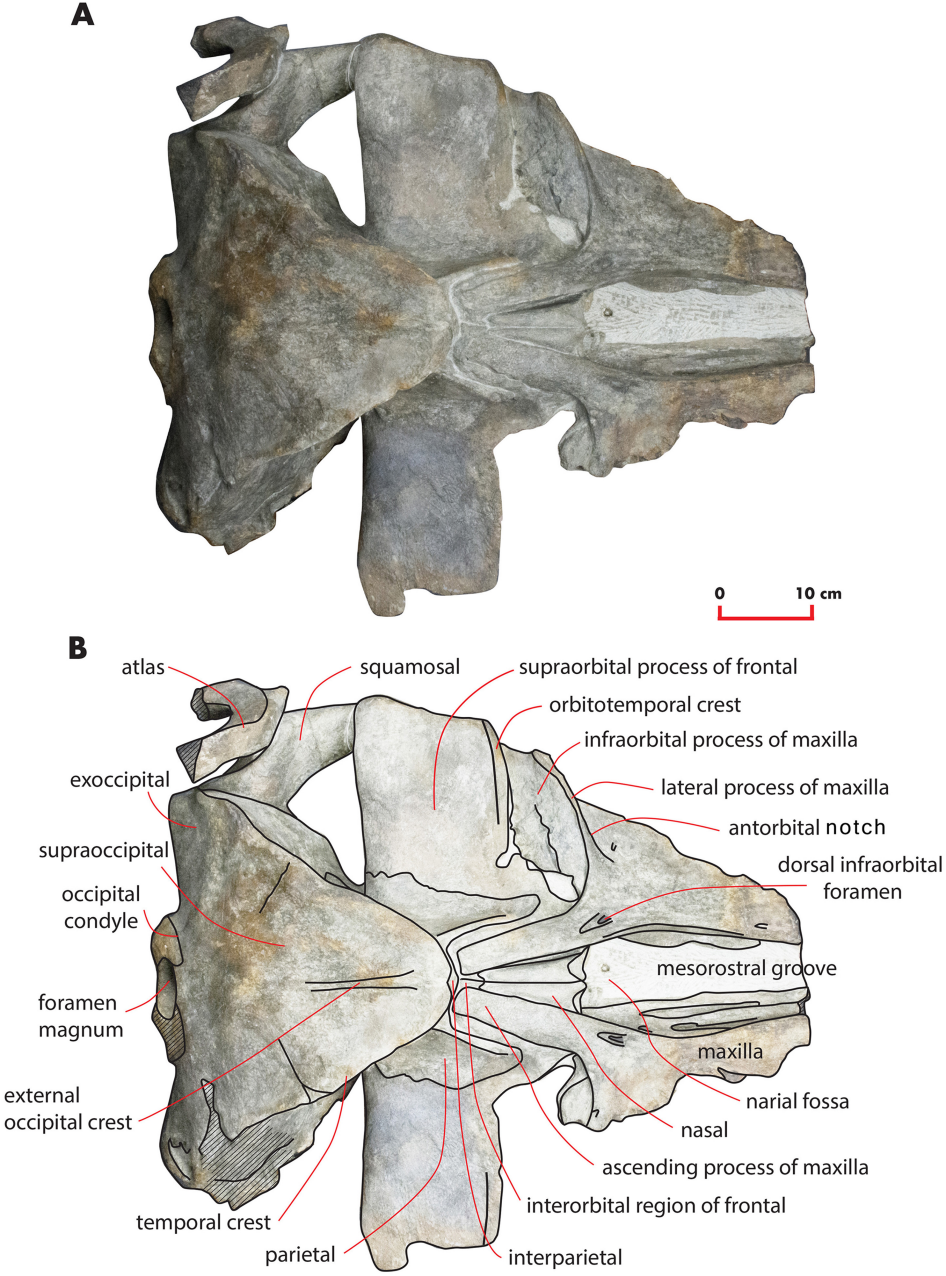
The holotype skull in dorsal view. Dorsal view of the holotype skull of *Nehalaennia devossi* (NMR 999100014035). (A) Photographic representation. (B) Interpretation. Scale bar equals 10 cm.

As preserved, the skull is 730 mm in length, 224 mm in maximum height and 621 mm in width at the postorbital process of the frontal. Additional measurements of the skull are reported in Table 1. The skull as a whole is illustrated in Figs. 3 (dorsal view), 4 (right lateral view), 5 (anterolateral view), 6 (ventral view), 7 (anterior view) and 8 (posterior view).

**Figure 4:**
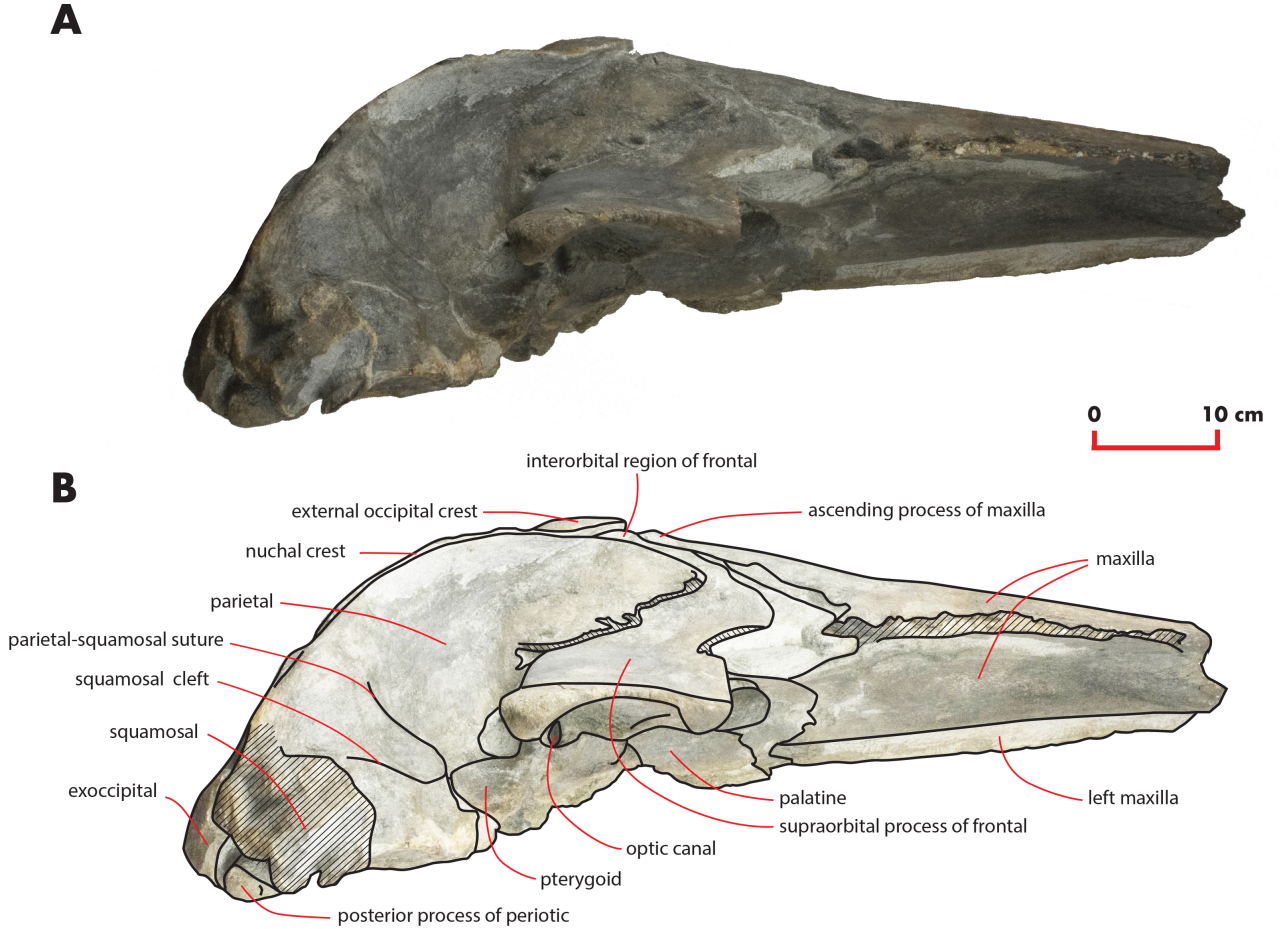
The holotype skull in right lateral view. Right lateral view of the holotype skull of *Nehalaennia devossi* (NMR 999100014035). (A) Photographic representation. (B) Interpretation. Scale bar equals 10 cm.

**Figure 5:**
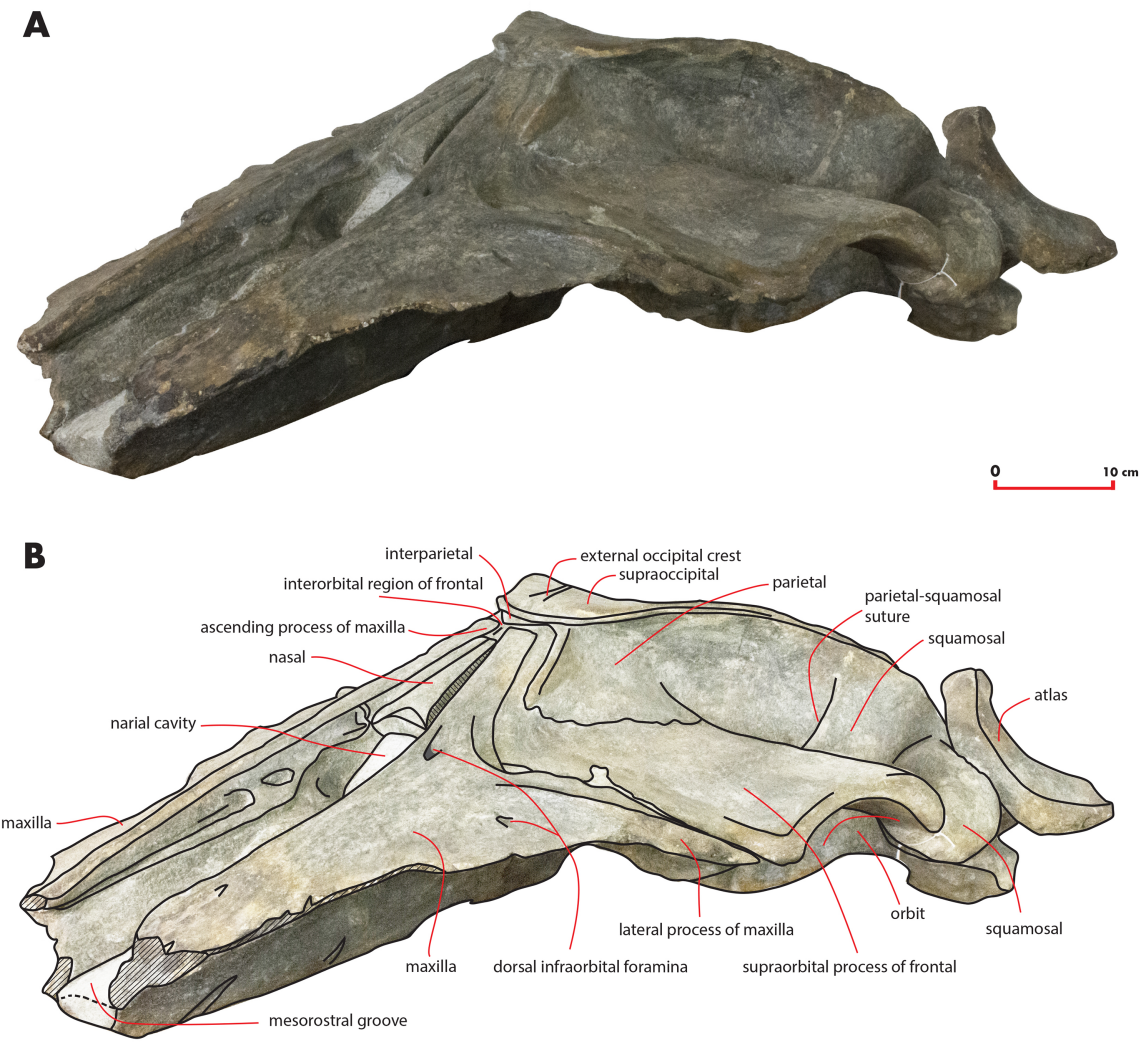
The holotype skull in anterolateral view. Anterolateral view of the left side of the holotype skull of *Nehalaennia devossi* (NMR 999100014035). (A) Photographic representation. (B) Interpretation. Scale bar equals 10 cm.

**Figure 6:**
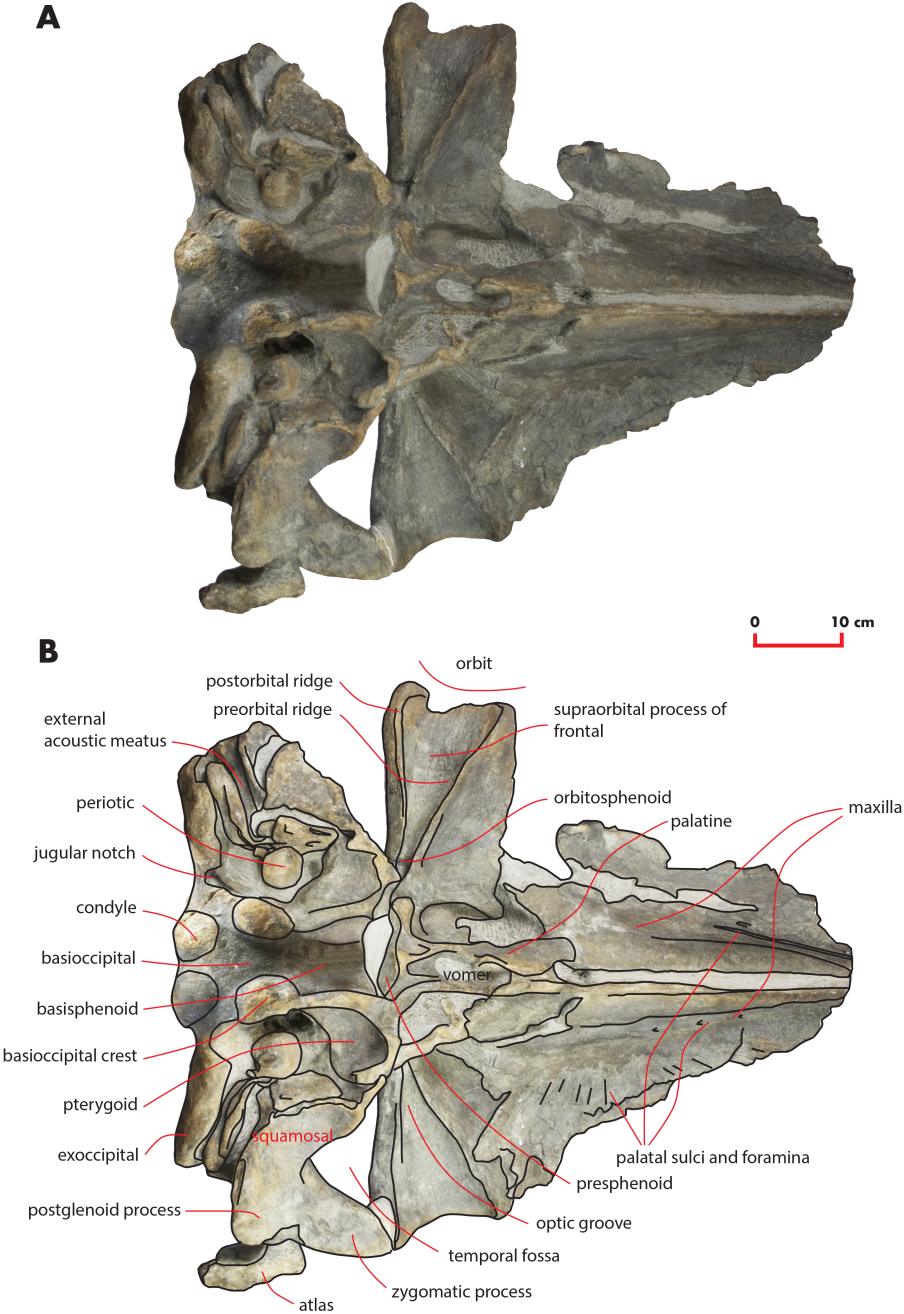
The holotype skull in ventral view. Ventral view of the holotype skull of *Nehalaennia devossi* (NMR 999100014035). (A) Photographic representation. (B) Interpretation. Scale bar equals 10 cm.

**Figure 7:**
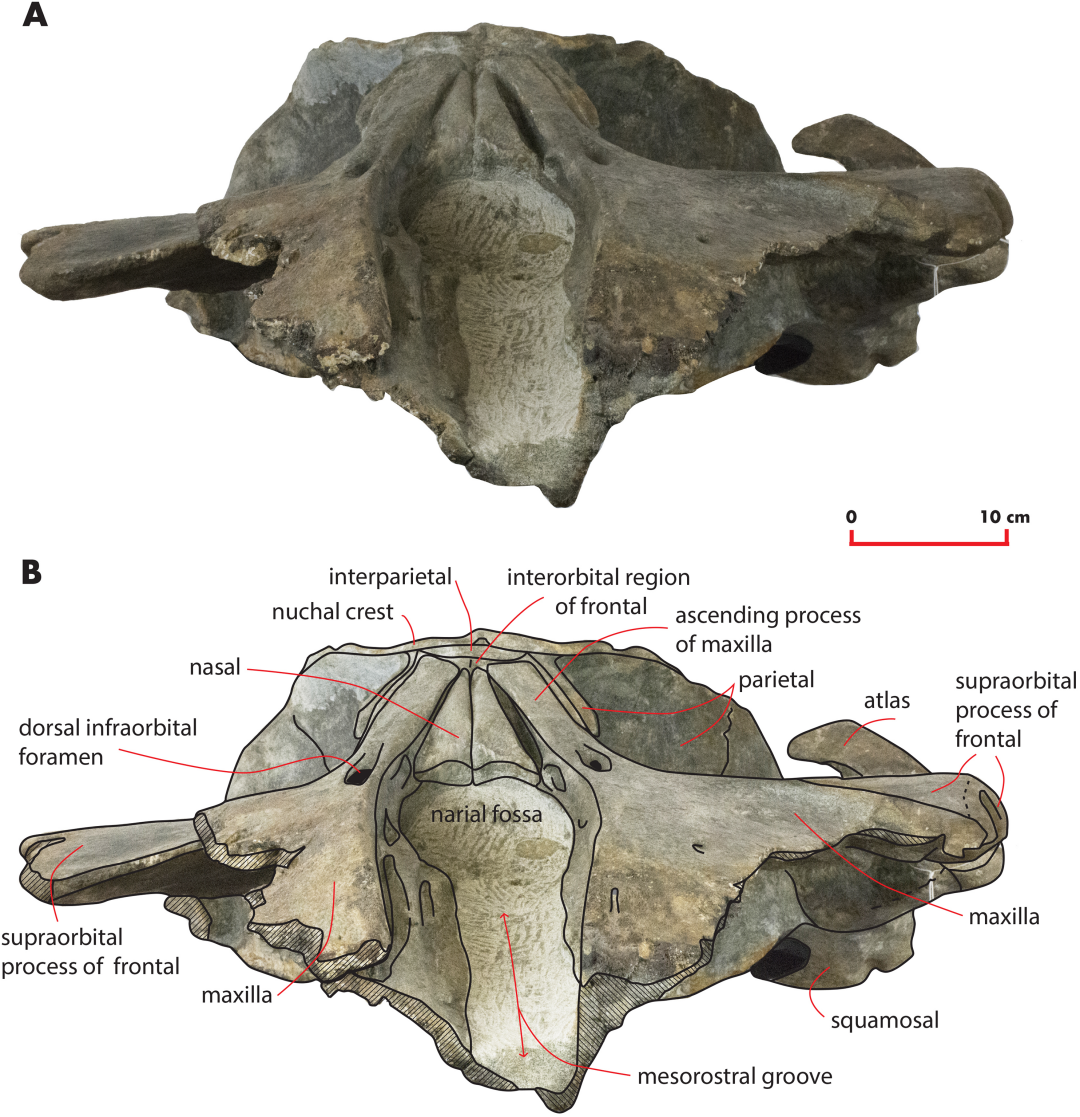
The holotype skull in anterior view. Anterior view of the holotype skull of *Nehalaennia devossi* (NMR 999100014035). (A) Photographic representation. (B) Interpretation. Scale bar equals 10 cm.

**Figure 8:**
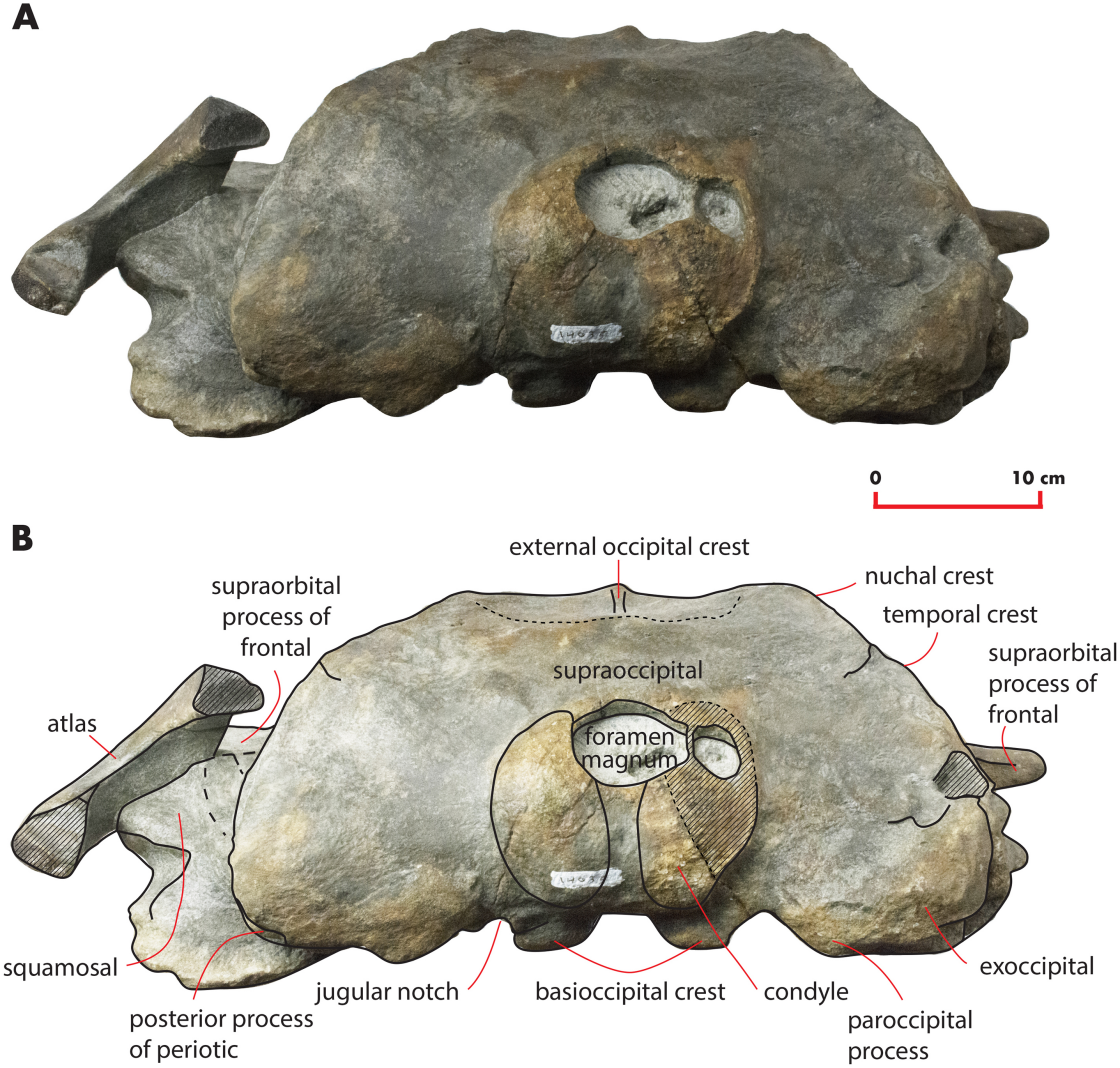
The holotype skull in anterior view. Anterior view of the holotype skull of *Nehalaennia devossi* (NMR 999100014035). (A) Photographic representation. (B) Interpretation. Scale bar equals 10 cm.

**Table 1:** Skull measurements.

Character	Measure
Maximum transverse diameter of the skull at postorbital process of frontal	621 (as preserved)
Maximum transverse diameter of the skull at the antorbital process of frontal	578
Maximum transverse diameter of the skull at the base of the ascending process of the maxilla	440 (value obtained by doubling the distance between the sagittal axis of the skull and the left part of the character)
Maximum transverse diameter of the skull at the apex of the zygomatic process of the squamosal	670 (value obtained by doubling the distance between the apex of the left zygomatic process of the squamosal and the sagittal axis of the skull)
Maximum transverse diameter of the skull at the posterior apex of the lambdoid crest	434
Maximum transverse diameter across the occipital condyles	154
Maximum length of the skull	730 (as preserved)
Maximum height of the skull	224

**Note:**

Linear measurements of the skull of *Nehalaennia devossi* (NMR 999100014035, holotype). Data in mm.

### Maxilla

**Figure 9:**
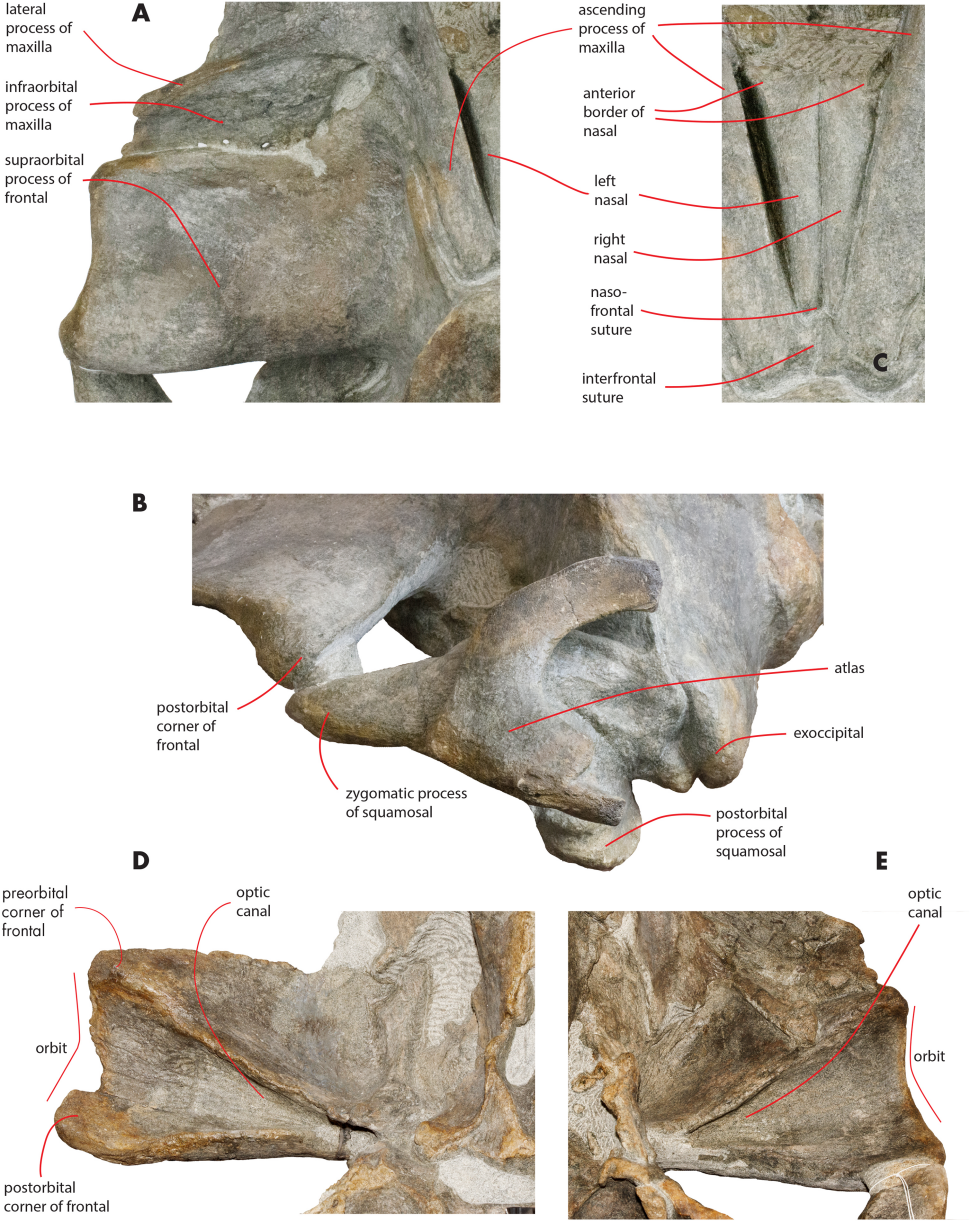
Osteological details of the holotype skull. Details of the osteology of the holotype skull of *Nehalaennia devossi* (NMR 999100014035). (A) Close-up view of the left ascending process of the maxilla and supraorbital process of the frontal showing the wide and long infraorbital process of themaxilla. (B) Posterolateral view of the left side of the skull showing the atlas and the relationships of zygomatic process of the squamosal and supraorbital process of the frontal. (C) Close-up view of the nasal bones. (D) Close-up view of the right supraorbital process of the frontal in ventral view. (E) Close-up view of the left supraorbital process of the frontal in ventral view. Not to scale.

The maxilla is transversely wide and mostly flat. Measurements are provided in Table 2. The lateral process of the maxilla is short and project posterolaterally; the antorbital notch is wide and concave anterolaterally. There is a wide space between the posterior border of the maxilla and the anterior border of the supraorbital process of the frontal (Figs. 3 and 9A). The infraorbital process of the maxilla is wide and long; it projects ventrally to the supraorbital process of the frontal and its posterior border terminates anteriorly to the antorbital process of the supraorbital process of the frontal. Three dorsal infraorbital foramina are located in the left maxilla. One of the foramina is wide and deep (maximum transverse diameter, 4.5 mm; maximum anteroposterior diameter, 13 mm) and located at the base of the ascending process of the maxilla; this foramen opens in a wide and elliptical fossa characterized by a maximum diameter that is anteroposteriorly oriented; the foramen is prolonged into a posteriorly directed sulcus. In the right maxilla, a large dorsal infraorbital foramen is observed at the base of the ascending process (maximum transverse diameter, four mm; maximum anteroposterior diameter, 13 mm); this foramen opens in an elliptical fossa and, as observed on the left side, is prolonged into a posteriorly directed sulcus. The foramina observed at the bases of the ascending processes of the maxillae together with their associated sulci are in the same position of the primary dorsal infraorbital foramina described by Marx, Bosselaers & Louwye (2016); we suggest that these foramina are homologous to the primary dorsal infraorbital foramina of Marx, Bosselaers & Louwye (2016). Three additional dorsal infraorbital foramina are observed in the right maxilla.

**Table 2:** Measurements of the rostrum.

Character	Right side	Left side
Maximum length of maxilla along medial border	413	440
Maximum length of maxilla up to the base of the ascending process	280	280
Length of ascending process of maxilla	155	156
Anterior width of ascending process of maxilla	47	46
Posterior width of ascending process of maxilla	25.5	26.6
Width of ascending process of maxilla at mid-length	37.6	32
Maximum width of narial cavity	113	
Posterior width of narial cavity	73	
Posterior width of maxilla from apex of lateral process to medial border of ascending process	116	236
Posterior width of maxilla up to base of ascending process	65	177
Anteroposterior length of lateral process of maxilla		19
Transverse width of lateral process of maxilla		64
Length of nasal along the medial border	107.3	107.1
Length of nasal along the lateral border	103.1^1^	116.3
Anterior width of nasal	25.5^1^	33
Width of nasal at mid-length	18.5	15
Posterior width of nasal	4.7	6
Width of both nasals at anterior border (doubling value for left nasal)	66	

**Notes:**

Measurements of the rostral bones of *Nehalaennia devossi* (NMR 999100014035, holotype). Data in mm.

1 As preserved.

The lateral borders of both maxillae are partially eroded (Fig. 3).

The ascending process of the maxilla is long and projects posterodorsally up to a point located a few mm anteriorly from the anterior end of the supraoccipital. The ascending process of the maxilla is interdigitated with frontal and the parietal. In dorsal view, the ascending process of the maxilla projects posteriorly and medially and converges towards the longitudinal axis of the skull. Medial and lateral borders of the ascending process of the maxilla are parallel; there is a posterolateral concavity between the lateral border of the ascending process of the maxilla and the posterior border of the maxilla; ascending process and posterior border are located at a right angle in dorsal view (Fig. 3). The posterior border of the ascending process of the maxilla is squared with the posterolateral corner located more posteriorly than the posteromedial corner. The posterior end of the ascending process of the maxilla is located more posteriorly than the nasofrontal suture. The ascending process of the maxilla is not clearly observed in lateral view as it is developed on the dorsal side of the skull and not on the lateral side.

**Figure 10:**
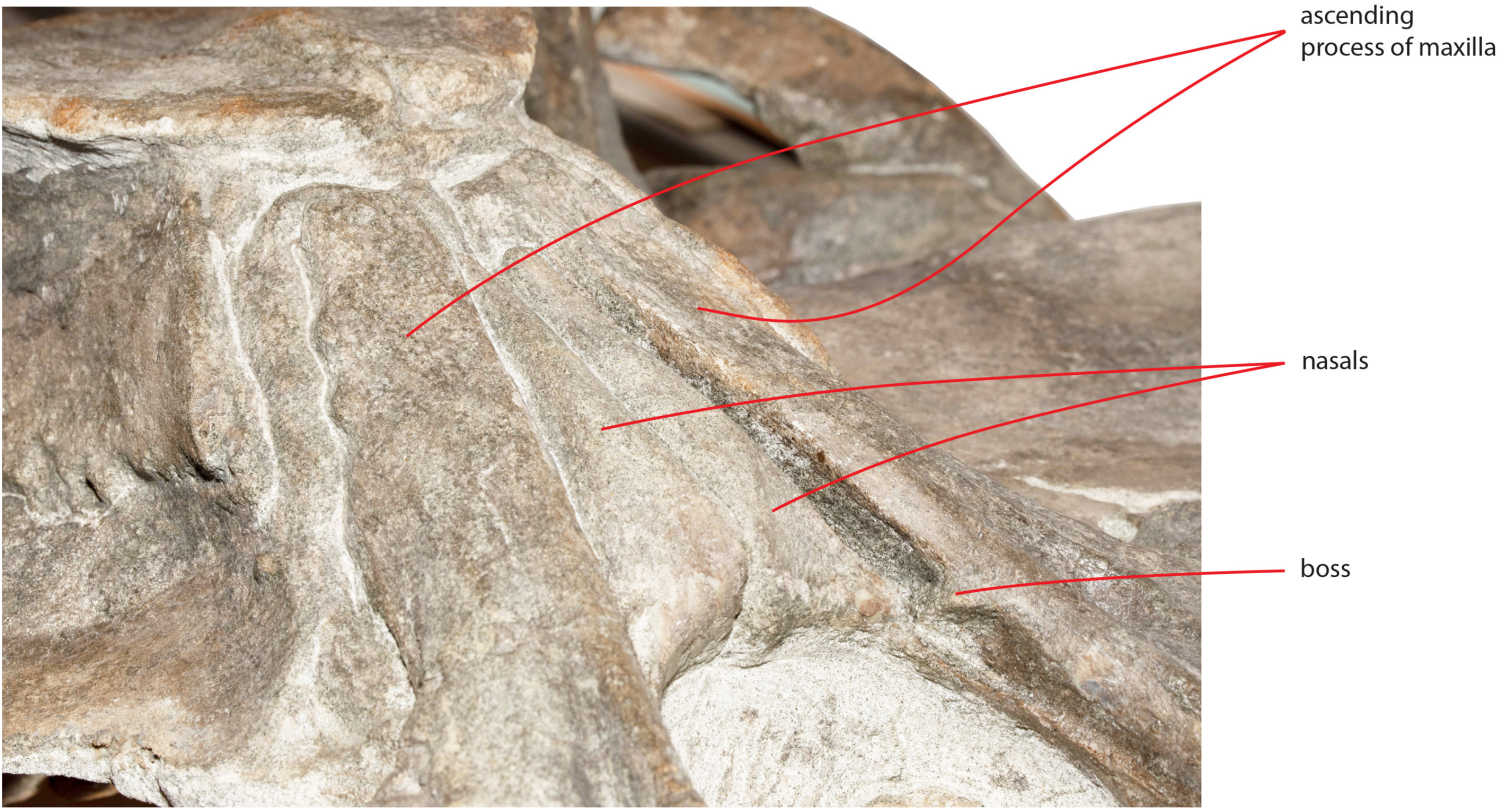
Detail of the medial view of the maxilla. Detail of the medial view of the maxilla showing the presence of a boss that is here interpreted as the posterior limit reached by the premaxilla.

A space is present between the medial border of the ascending process of the maxilla and the lateral border of the nasal; such a space is particularly evident on the left side (see previous paragraph) probably due to postmortem deformation of the corresponding maxilla. It is not completely clear whether the posterior portion of the premaxilla occupied this space or not. In fact, a boss is present on the medial border of the maxilla that could mark the posterior end of the premaxilla slightly in front to the anterolateral corner of the nasal (Fig. 10). Such a boss is present only on the left maxilla but its presence suggests that the premaxilla was not developed more posteriorly.

The narial fossa is relatively wide and long. Anteriorly its borders mildly converge to the mesorostral groove but posteriorly it narrows abruptly.

In ventral view, the maxilla shows a pronounced ventral keel that is laterally paralleled by an anteroposterior concavity (Fig. 6). The grooves for the vasculature of the baleen-bearing epithelium are present on the ventral surface; the more posterior grooves project posterolaterally and the more anterior ones project anterolaterally. A single groove located on the right maxilla shows a different orientation being its main axis oriented from a posterolateral point to an anterolateral point. Such a groove is 155 mm in length and 15 mm in width. Four additional foramina are observed in the concavity that parallels the ventral keel; the foramina are prolonged into short grooves mainly parallel to the longitudinal axis of the skull.

### Vomer

Only two squared fragments of the posterior portion of the vomer can be observed in the holotype skull (Fig. 6). These fragments belongs to the nasal plate of the vomer and are 131 mm in length in total and their maximum width is 73 mm.

### Nasal

In dorsal view, the nasal has a triangular shape (Fig. 9C). The posterior border of the nasal is deeply inserted within the interorbital region of the frontal. The posterior border is posteriorly triangular and pointed. The anterior border is anteriorly concave; the anterolateral corner of the nasal is located more anteriorly than the anteromedial corner. The anterolateral corner of the left nasal terminates closely to a boss emerging from the medial surface of the maxilla that probably marks the posterior end of the premaxilla. The dorsal surface of the posterior portion of the nasal is flat and substantially horizontal but, more anteriorly, it becomes dorsally concave and projects anteroventrally. Measurements are provided in Table 2.

### Frontal

**Figure 11:**
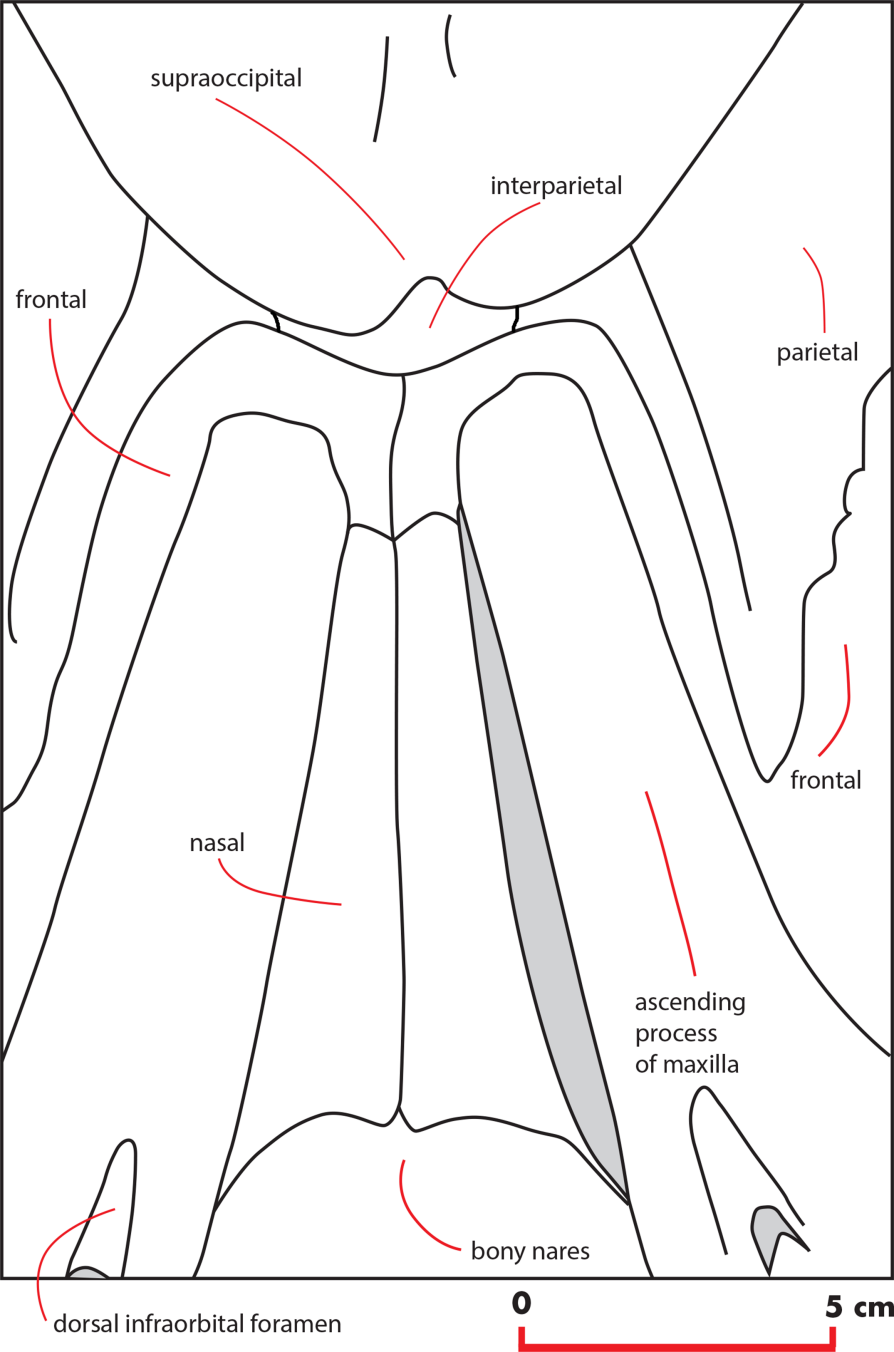
Vertex structure of the holotype skull. Line drawing showing the vertex structure of the holotype skull of *Nehalaennia devossi* (NMR 999100014035). Scale bar equals five cm.

The interorbital region of the frontal is exposed in dorsal view posteriorly and laterally to the ascending process of the maxilla (Fig. 11). The posterior border of the interorbital region is anteriorly convex and surrounds the posterior half of the ascending process of the maxilla. The maximum width of the interorbital region of the frontal (between the depressions of the supraorbital processes of the frontal) is 74.6 mm and its maximum length along the sagittal axis of the skull is 25 mm. Additional measurements of the frontal are provided in Table 3.

**Table 3:** Measurements of the skull vault.

Character	Right measure	Axial measure	Left measure
Maximum transverse width of SOP at the middle of the orbit			239
Maximum transverse width of SOP at the postorbital process			256
Length of SOP medially			207
Length of SOP at the middle of its maximum width (as measured at the middle of the orbit)			161
Maximum anteroposterior length of orbit	118		161
Height of orbit			31
Total length of parietal	290		
Length of parietal squama	95		
Anteroposterior diameter of interparietal		11	
Maximum width of interparietal		81	
Maximum length of the glenoid fossa of the squamosal	228		
Maximum width of the glenoid fossa of the squamosal	113		
Maximum width of the zygomatic process of the squamosal at the anterior end	57		
Length of supraoccipital		295	
Anterior width of supraoccipital		83	
Width of supraoccipital at anterolateral concavity		185	
Maximum width of supraoccipital		365	
Anteroposterior diameter of occipital condyle			114
Lateromedial diameter of occipital condyle	64		56
Anteroposterior diameter of foramen magnum		57.5	
Lateromedial diameter of foramen magnum		67.9	
Distance between the posterolateral corner of exoccipital and center of foramen magnum		220	

**Notes:**

Measurements of the skull vault of the skull of *Nehalaennia devossi* (NMR 999100014035, holotype). Data in mm.

Caption: SOP, supraorbital process of frontal.

The interfrontal suture is open. The nasofrontal suture is transversely short and anteriorly convex as a subtle portion of the infraorbital region of the frontal is interposed between the posterior apices of the nasals. Posteriorly, the interorbital region of the frontal is in sutural contact with the interparietal and with the parietals (Fig. 11).

**Figure 12:**
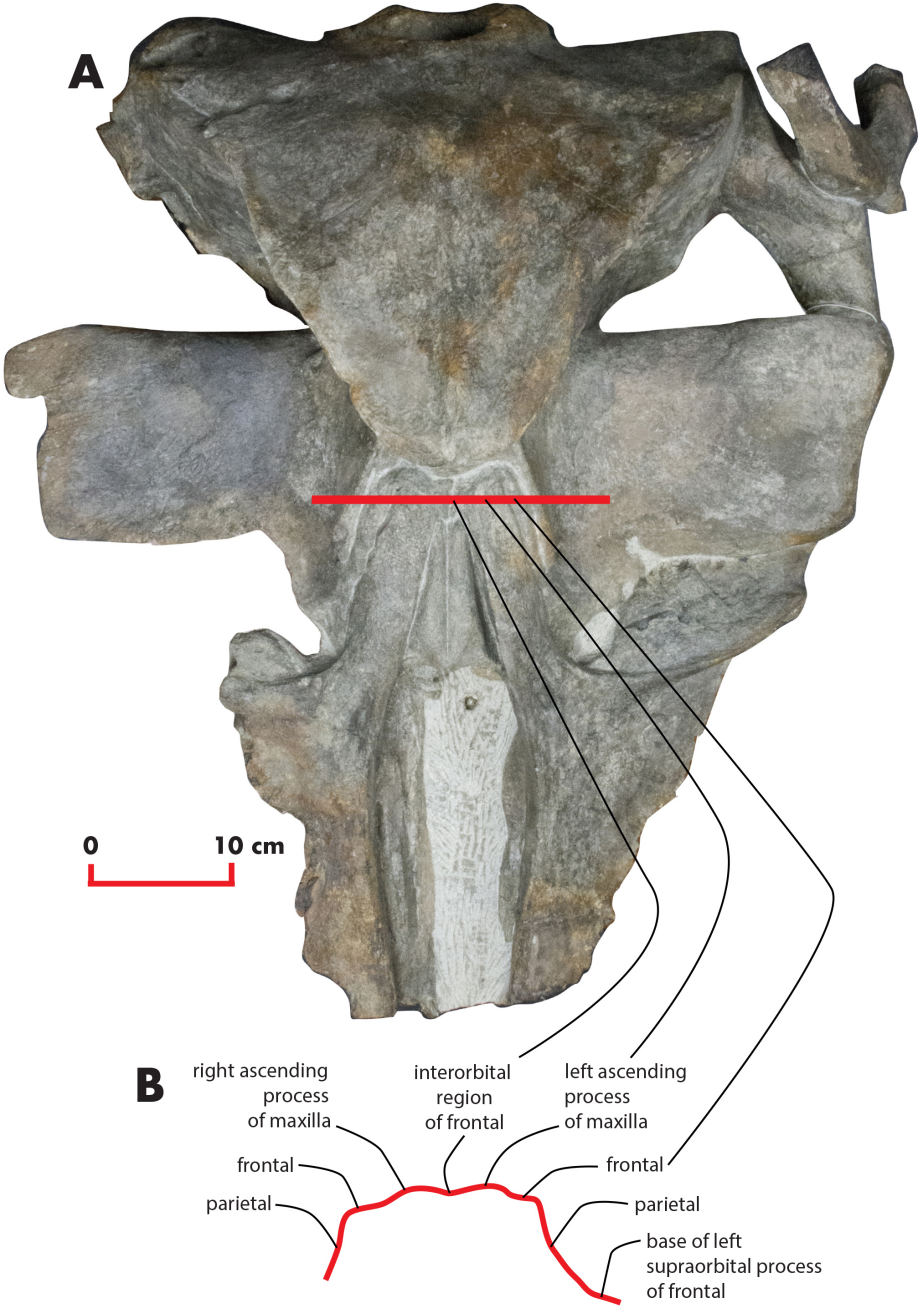
Cross section of the skull. Cross section of the holotype skull of *Nehalaennia devossi* (NMR 999100014035) showing the depression of the supraorbital process of the frontal and the scarce development of the temporal crest at the level of the orbit. (A) The skull in dorsal view with the position of the section shown as a horizontal line crossing the middle of the orbits. (B) Section in anterior view. Not to scale.

The supraorbital process of the frontal is abruptly depressed from the interorbital region of the frontal (Figs. 7 and 12). It is wide, anteroposteriorly flat and slightly relieved lateromedially. In anterior view, the depression of the supraorbital region of the frontal is mild since parietal and supraorbital process form a wide laterodorsal concavity. The orbitotemporal crest is strongly reduced to a slight relief paralleling the anterolateral border of the supraorbital process of the frontal close to the orbit.

The anteromedial corner of the supraorbital process of the frontal is located more anteriorly than the remainder of the anterior border. The anterior border of the supraorbital process of the frontal forms a wide anterior concavity and then projects laterally. Both the anterior and the posterior borders of the supraorbital process of the frontal project lateroposteriorly.

The orbit is long and dorsoventrally low (Figs. 4 and 5). It opens laterally and is bordered by a small antorbital process and by a robust postorbital process. The latter projects more posteriorly and slightly ventrally; the articular facet for the articulation with the zygomatic process of the squamosal is not evident. However, the distal portion of the posterior border of the supraorbital process of the frontal shows an anterior orientation differing from the more medial portion which has a clear posterolateral orientation.

In ventral view, a triangular optical channel, that widens while approaching the orbital rim, characterizes the supraorbital process of the frontal (Figs. 6 and 9D). The orbital channel is anteriorly bordered by a remarkably protruding crest and, posteriorly, by a sharp crest.

### Parietal

In lateral view, the parietal shows an anteriorly protruding frontal border and a ventrally protruding posterior portion (Figs. 4 and 5). The frontal border is anteriorly triangular and its anterior border reaches a point located more anteriorly than the posterior end of the ascending process of the maxilla. The frontal border is superimposed on the vertical portion that leads, more laterally, to the supraorbital process of the frontal. In this sense, parietal, maxilla and frontal are largely interdigitated as in all known balaenopterids. The anterior end of the frontal border of the parietal is located 73 mm anteriorly to the posterolateral corner of the ascending process of the maxilla and 62 mm anteriorly to the posterolateral corner of the left nasal. As demonstrated by the cross section of the skull (Fig. 12), the lateral surface of the parietal is laterally concave but not as observed in living balaenopterid species where the depression of the parietal lateral to the interorbital region of the frontal is nearly vertical. The anterior portion of the parietal widens on the posteromedial portion of the supraorbital process of the frontal. The supraoccipital border of the parietal projects laterally starting 95 mm from its anterior end and terminates ventrally to the lateral borders of the supraoccipital. In this way, the supraoccipital border of the parietal contributes to the formation of the temporal crest. The temporal crest projects laterally forming a lateral concavity in the medial wall of the temporal fossa. The concavity is particularly deep posteriorly to the posterior border of the supraorbital process of the frontal. Because of this, the anterior portion of the medial wall of the temporal fossa cannot be observed in dorsal view (Fig. 3).

**Figure 13:**
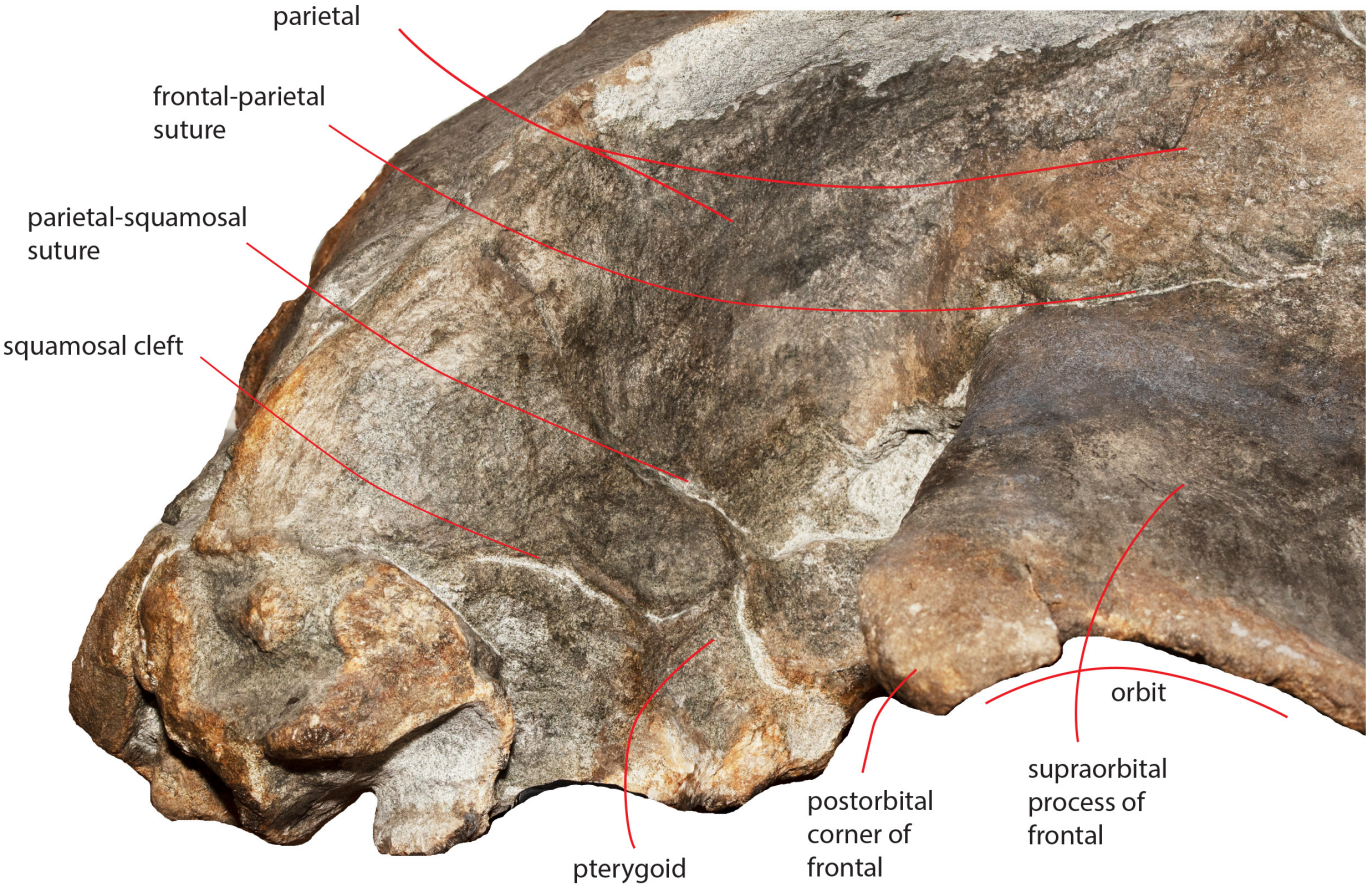
The right temporal fossa of the holotype skull. Close-up view of the right temporal fossa of the holotype skull of *Nehalaennia devossi* (NMR 999100014035) showing the squamosal cleft, the parietal-squamosal suture and the presence of the pterygoid in the temporal fossa.

The parietal-squamosal suture starts from the posterodorsal corner of the pterygoid and projects posterodorsally in a sinuous way (Fig. 13); it projects more posteriorly when approaching the posterolateral border of the supraoccipital and reaches a point located 117 mm anteriorly to the posterior apex of the nuchal crest (Figs. 4 and 5).

### Interparietal

The anterodorsal borders of the parietals are divided by the interposition of a transversely elongated interparietal. The interparietal shows an anteriorly protruding medial portion that is insinuated within the interorbital region of the frontal (Fig. 11). Laterally to this medial portion, two narrow elements of the interparietal project laterally and contact the parietal posteriorly to the interorbital region of the frontal.

### Supraoccipital

The supraoccipital is elongated and triangular in dorsal view and shows sinuous lateral borders (Fig. 3). A lateral concavity in the lateral border is located approximately at one-fourth of the total length of the supraoccipital. The lateral border of the supraoccipital protrudes laterally and superimposes on the supraoccipital border of the parietal forming the temporal crest. The laterally-protruded temporal crest overhangs the temporal fossa and makes it impossible to observe the medial wall of the anterior portion of the temporal fossa in dorsal view. The anterior border of the supraoccipital is rounded and narrow. There is a sagittal notch in the anterior border that could be due a to postmortem erosional process.

Anteriorly, the dorsal surface of the supraoccipital bears a 71-mm-long external occipital crest (Fig. 3). The posterior end of the ridge becomes dorsally rounded and is interposed between two narrow fossae characterized by an elliptical shape. These fossae are 81 mm in length and 82 mm in width.

### Exoccipital

In posterior view, the exoccipital is triangular (Fig. 8). Its ventral border is lower than the ventral border of the occipital condyle. The foramen magnum is elliptical and dorsoventrally compressed. The occipital condyles are dorsally narrow but, starting from the middle of the foramen magnum, widen remarkably. The articular surface for the atlas is transversely and dorsoventrally convex and can be observed in the left condyle; the surface of the right condyle is rough suggesting the lack of the epyphysis. The intercondyloid notch is slightly developed and has the shape of a narrow concavity along the dorsoventral axis. A wide hole is present on the right side of the foramen magnum that is an artifact probably due to postmortem taphonomic processes.

### Squamosal

Near the parietal-squamosal suture, the squamosal plate is laterally convex. In dorsal view, the squamosal plate projects posteromedially from the parietal-squamosal suture; it protrudes posteriorly forming a triangular surface posteriorly bounded by the posterior apex of the nuchal crest. The apex terminates more anteriorly than the articular surface of the occipital condyles in dorsal view. The squamosal plate does not protrude within the temporal fossa in dorsal view. A dorsoventral furrow is observed medially to the zygomatic process of the squamosal.

The squamosal cleft is present. It starts from the posterior border of the pterygoid at the pterygoid-squamosal suture only a few mm ventrally to the point where the squamosal, the parietal and the pterygoid meet (Fig. 13). It projects posteriorly showing a sinuous development. Approaching the posteromedial portion of the zygomatic process of the squamosal, it turns anteroventrally and terminates within the dorsoventral furrow medial to the emergence of the zygomatic process of the squamosal. The length of the right squamosal cleft is 101 mm.

**Figure 14:**
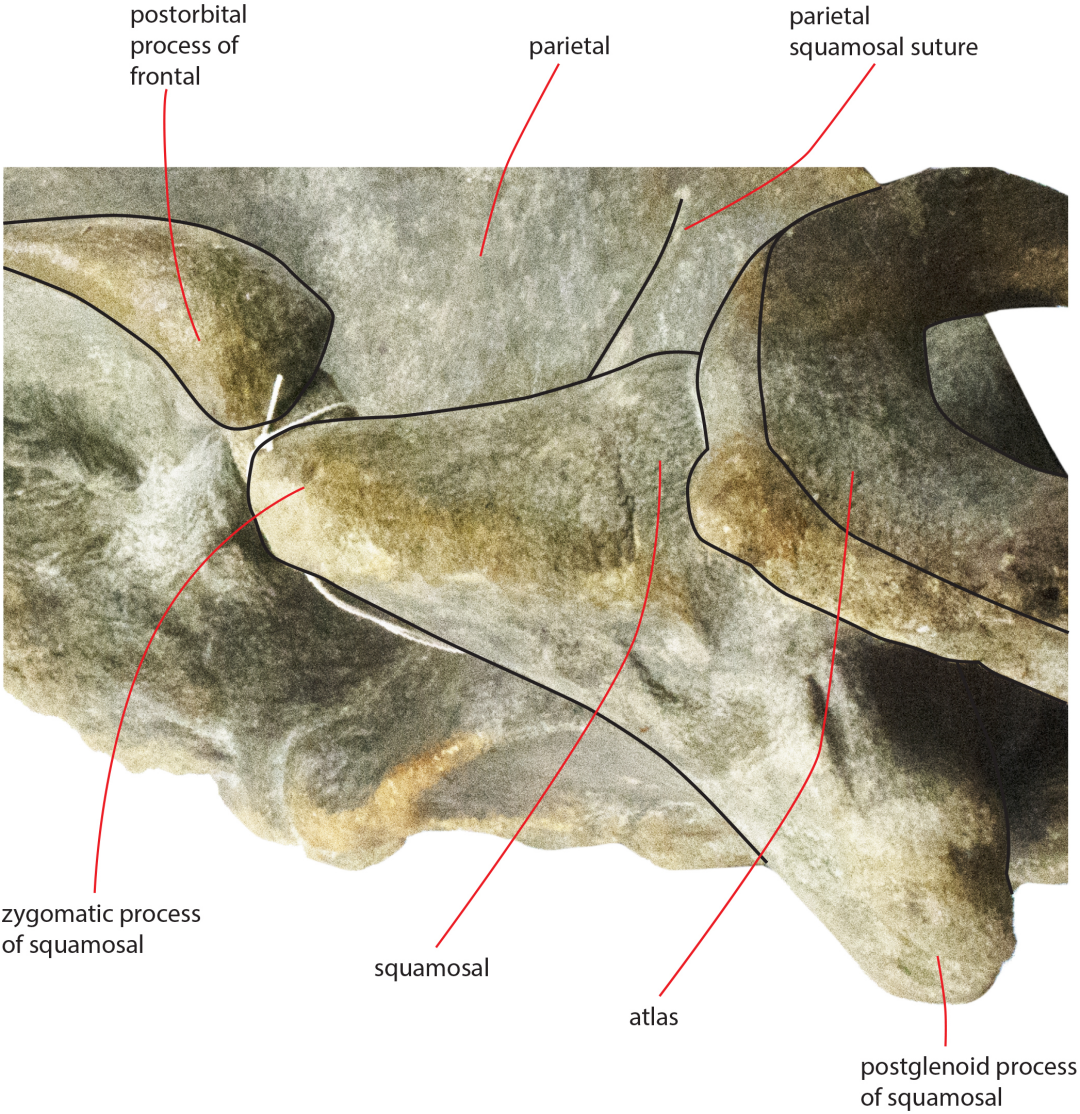
Zygomatic process of the squamosal of the holotype skull. Close-up view of the left zygomatic process of the holotype skull of *Nehalaennia devossi* showing its triangular shape and the crescent-shape of the glenoid fossa of the squamosal.

The zygomatic process of the squamosal is triangular in lateral view (Fig. 14). It projects anteriorly to reach a point located very closely to the postorbital process of the supraorbital process of the frontal. Posteriorly to the zygomatic process of the squamosal, an anterodorsally rounded supramastoid crest is well evident in lateral view. Laterally and ventrally to the supramastoid crest there are three different fossae for the attachment of neck muscles. In dorsal view, the zygomatic process of the squamosal is anteriorly divergent from the longitudinal axis of the skull along its whole length (i.e., the zygomatic process of the squamosal is not twisted medially).

The glenoid fossa of the squamosal is long and wide (see Table 3) and is posteriorly bounded by a moderately well developed postglenoid process of the squamosal. The glenoid fossa of the squamosal shows a ventral concavity along the anteroposterior axis and a ventral convexity along the transverse axis. The postglenoid process of the squamosal is oriented posteroventrally like in modern balaenopterid species.

The foramen ovale is not well distinguishable probably because of the breakage of the pterygoid. It probably opened anteriorly to the anterior process of the periotic as shown in Fig. 14.

### Vertex

The vertex shows the typical characters of an advanced balaenopterid whale (Fig. 11). The anterior border of the supraoccipital projects anteriorly and reaches a point more anterior than the apex of the zygomatic process of the squamosal. The anterior border of the supraoccipital is separated from the interorbital region of the frontal by the interposition of a short and wide interparietal bone. The interorbital region of the frontal surrounds the ascending process of the maxilla laterally and posteriorly. The anterior portion of the parietal runs parallel to the lateral portion of the interorbital region of the frontal and reaches a point located more anteriorly than the posterior end of the ascending process of the maxilla. The nasofrontal suture is located well within the interorbital region of the frontal; because of this, the posterior end of the nasal is located more posteriorly than the anterior border of the supraorbital process of the frontal. Part of the interorbital region of the frontal is located between the posteromedial borders of the nasals.

### Temporal fossa

In dorsal view, the temporal fossa is anteroposteriorly short and triangular in shape (Fig. 3). Anteriorly, it is bordered by the straight posterior border of the supraorbital process of the frontal that projects laterally; posteromedially it is bordered by the posterior portion of the parietal and by the anterior portion of the squamosal and posterolaterally it is entirely bordered by the zygomatic process of the squamosal. In lateral view, the medial wall of the temporal fossa is formed by a mosaic of bones including parietal, squamosal and pterygoid (Fig. 13). The alisphenoid is not exposed and cannot be observed in lateral view. The parietal-pterygoid suture runs anteroposteriorly for a few centimeters. The parietal-squamosal suture starts from the posterodorsal corner of the pterygoid that is exposed in the temporal fossa. The parietal-squamosal suture runs posteriorly and dorsally in a sinuous way up to the posterolateral portion of the supraoccipital. A squamosal cleft starts from the vertically-oriented posterior border of the pterygoid (pterygoid-squamosal suture) and runs posteriorly in a sinuous way. Around 90 mm posterolaterally from its starting point, the squamosal cleft points anteriorly and ventrally forming a sharp corner. No postparietal foramen can be observed in the specimen.

### Basicranium

In ventral view, the skull shows the sequence formed by presphenoid, basisphenoid and basioccipital and most of the lateral structures forming parts of squamosal and exoccipital, and the periotics. The presphenoid is short and shows a wide longitudinal concavity that is triangular in shape (Table 4). The body of the presphenoid is robust and has two wings (orbitosphenoid) that are partially covered by the vomer and by the hamular process of the pterygoid (Fig. 6).

**Table 4:** Basicranial measurements.

Character	Right measure	Axial measure	Left measure
Presphenoid: length		58	
Presphenoid: maximum width (as preserved)		100	
Presphenoid: maximum height		26	
Basisphenoid-basioccipital length			
Maximum width of basioccipital across basioccipital descending processes		122	
Maximum anteroposterior diameter of the basioccipital descending process	65		65
Maximum transverse diameter of the basioccipital descending process	34		39
Distance between the posterior edge of the postglenoid process of the squamosal and the anterior end of the zygomatic process of the squamosal			229
Maximum length of the foramen lacerus posterius	94		70
Maximum width of the foramen lacerus posterius along the anterior border	75		51

**Note:**

Measurements of the basicranium of NMR 999100014035. Data in mm.

The basisphenoid is separated from the presphenoid by a wide space filled by matrix. The basisphenoid is an elongated platform showing a ventral convexity along the transverse axis. The basioccipital shows a ventral concavity along the transverse axis as a strong basioccipital crest is protruded from its lateral border. The basioccipital crest is tubercle-like and has a wide surface for the attachment of the hyoid. In ventral view, the outline of the basioccipital crest is oval and its articular facet for the hyoid is ventrally convex.

In ventral view, the exoccipital is straight and oriented posterolaterally with respect to the longitudinal axis of the skull. Its posterolateral corner is located more medially than the postglenoid process of the squamosal but protrudes posteriorly in a remarkable way. The paroccipital process is bulbous and appears separated from the lateroventral portion of the exoccipital by a lateromedial concavity.

The pterygoid is damaged on both sides of the skull (Fig. 6) as its ventral-most portion is broken. The hamular process of the right pterygoid is broken and moved from its original position as it is found very close to the presphenoid. In ventral view, the palatal surface is strong; it is formed by a lateral portion proceeding to the temporal fossa and by a medial portion that parallels the longitudinal axis of the skull. Lateral and medial portions form a right angle in ventral view. The palatal surface forms the anterior border of the pterygoid fossa whose dorsal surface is posteroventrally concave and is bordered by a posterolateral edge that is oriented posterolaterally with respect to the longitudinal axis of the skull. In lateral view, the lateral portion of the palatal surface projects dorsally and posteriorly forming a wide and approximately rectangular portion entering the temporal fossa. Part of the pterygoid is exposed in the temporal fossa and is bordered by the parietal dorsally and by the squamosal posteriorly. The parietal-squamosal suture begins from the posterodorsal corner of this portion of the pterygoid and the squamosal cleft starts from its posterior border (Fig. 13).

In ventral view, the squamosal is oriented posterolaterally. The zygomatic process of the squamosal starts abruptly from the main body of the bone and projects laterally and anteriorly. The squamosal does not bulge into the temporal fossa. The posteroventral edge of the squamosal has a sinuous development being posteriorly concave in its medial portion and posteriorly convex along the posterior outline of the postglenoid process. The medial portion corresponds to the concavity of the external acoustic meatus which is anteroposteriorly long and transversely wide (anteroposterior diameter, 30 mm; transverse width, 83 mm on the left side). Posteriorly, the external acoustic meatus is bordered by the posterior process of the periotic that is still in articulation. The glenoid cavity of the squamosal is ventrally concave along its anteroposterior axis but ventrally convex along its transverse axis.

**Figure 15:**
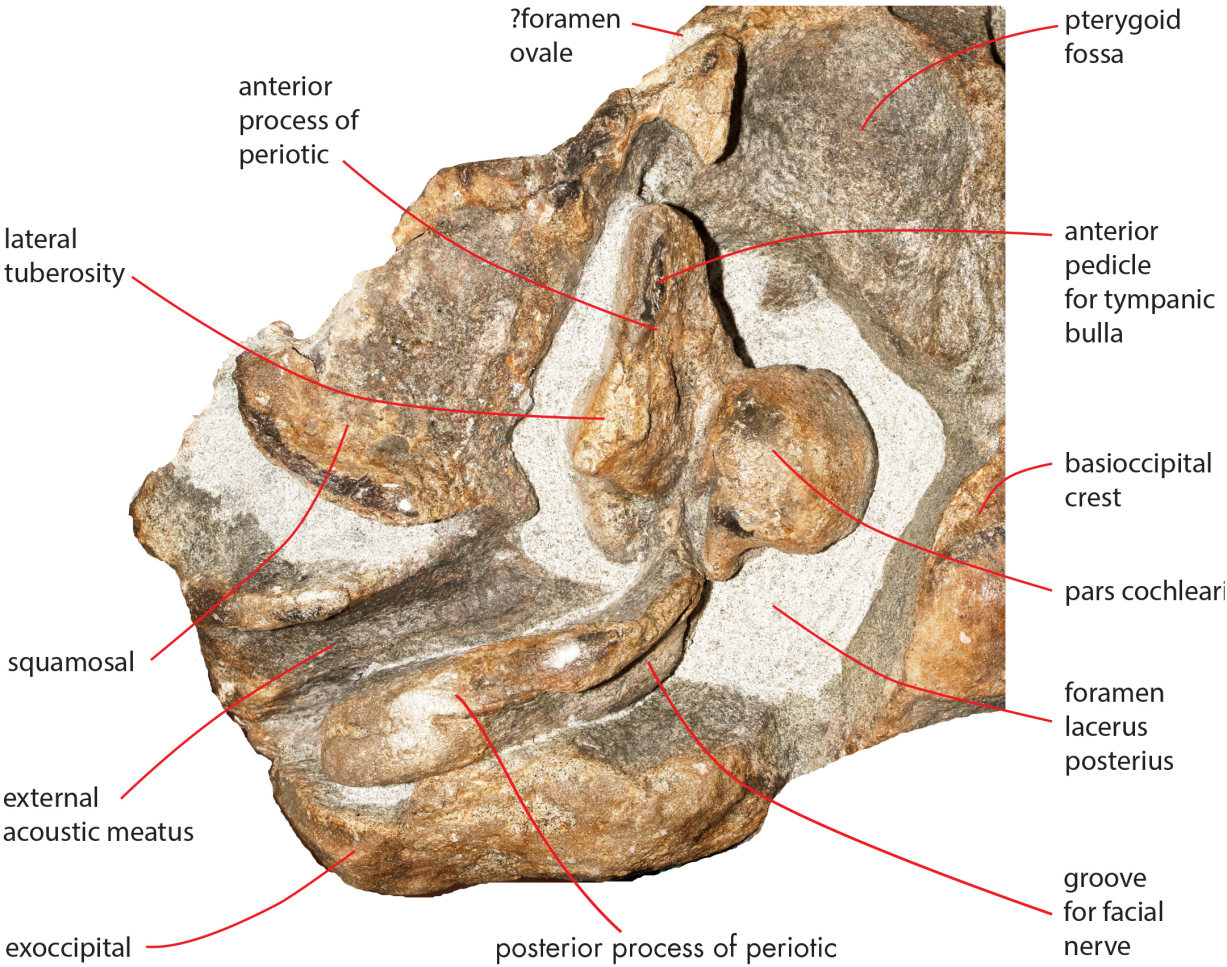
Osteological details of basicranium in the holotype skull. Right posterolateral portion of the holotype skull of *Nehalaennia devossi* (NMR 999100014035) in ventral view showing the periotic and the surrounding structures including the foramen lacerus posterius.

The foramen lacerus posterius is wide and long (Fig. 15). The mediall wall of the foramen lacerus posterius is formed by the lateral surface of the medial portion of the palatal surface of the pterygoid and by the lateral surface of the basioccipital crest and is mainly straight. Anteriorly, the foramen lacerus posterius is bordered by the posterior edge of the pterygoid fossa that is anterolaterally oriented. A small portion of squamosal forms the anterolateral wall of the foramen lacerus posterius anterior to the anterior process of the periotic. The periotic forms most of the lateral border of the foramen lacerus posterius. Posteromedially, a few mm laterally from the posterolateral corner of the basioccipital crest, a narrow jugular notch runs anteroposteriorly. Posterior process of the periotic and exoccipital form the posterior wall of the foramen lacerus posterius.

### Periotic

**Figure 16:**
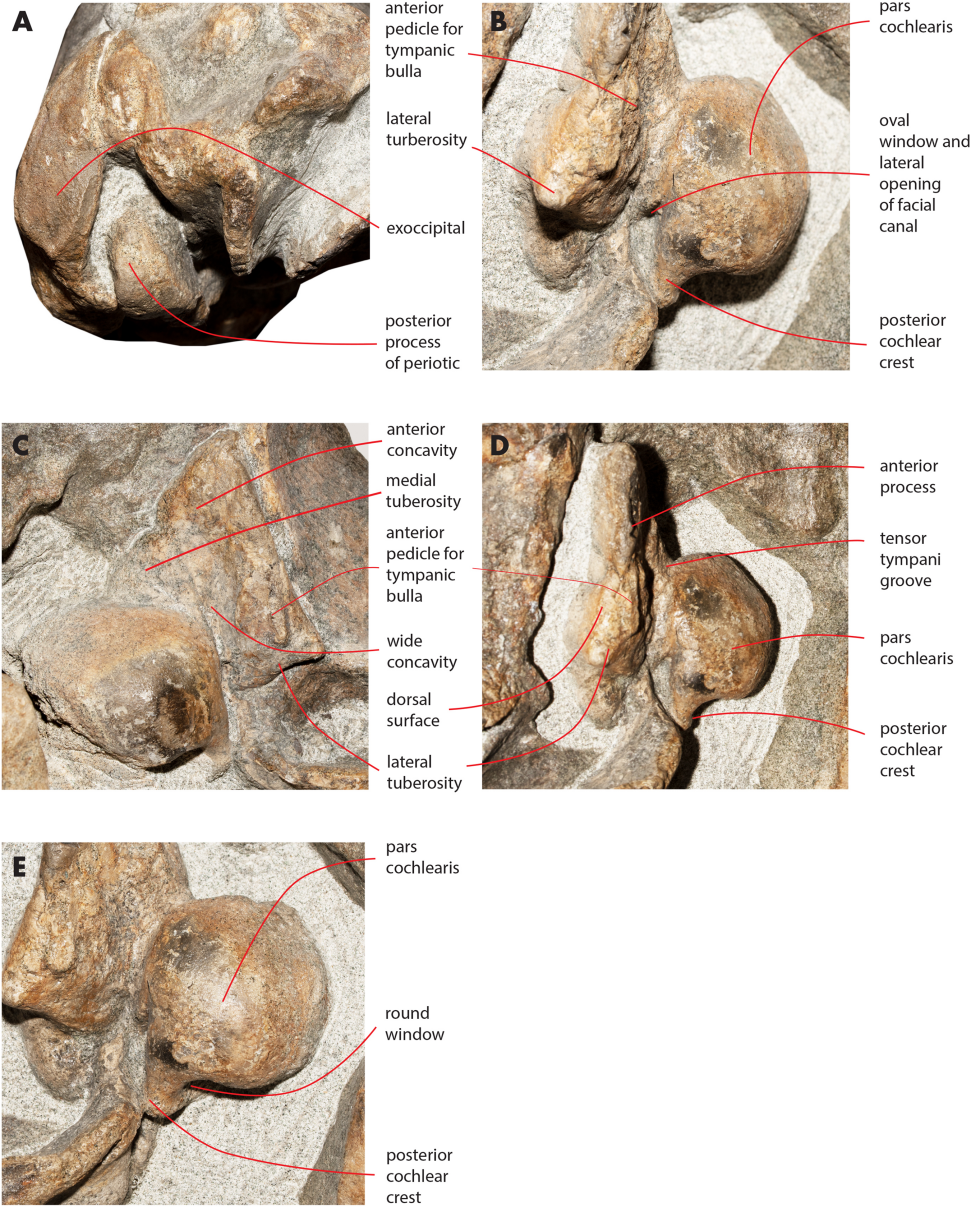
Details of the ear region of the holotype skull. Details of the osteology of the ear region of the holotype skull of *Nehalaennia devossi* (NMR 999100014035). (A) Exposure of posterior process of the periotic on the lateral wall of the skull. (B) Pars cochlearis, anterior process and proximal part of posterior process of the right periotic in lateroventral view. (C) Details of the ventral surface of the anterior process showing the fossae described in the text. (D) Details of the dorsal surface of the right periotic. (E) Details of oval window and associated structures on the right periotic.

Both periotics are still in articulation (Fig. 16). Both have broken pedicles for the tympanic bulla. The posterior process is relatively short, flat and robust (Table 5). In lateral view, the posterior end of the posterior process of the periotic is exposed between the exoccipital and the postglenoid process of the squamosal (Fig. 16A). The distal-most portion of the posterior process is more robust and thick than the medial portion. A groove for the transit of the facial nerve can be observed posteromedially along the ventral surface of the posterior process; such a groove is bordered by a robust and wide crest. The groove for the facial nerve is highly concave and leads to a foramen located between the posterior process and the pars cochlearis; this foramen is still filled by matrix (Fig. 16B). Approaching the pars cochlearis, the posterior process forms a small portion (22 mm in length) that narrows and turns.

**Table 5:** Measurements of periotics.

Character	Right periotic	Left periotic
Posterior process of the periotic: length	94	104
Posterior process of the periotic: maximum diameter (distal)	26	32
Posterior process of the periotic: maximum diameter (mid-length)	20	21
Posterior process of the periotic: maximum diameter (medial)	15	16
Posterior process of the periotic: maximum height (distal exposure)	36	32
Anterior process of the periotic: length	41	51
Anterior process of the periotic: maximum width (posterior)	45	55
Pars cochlearis: maximum anteroposterior diameter	45	43
Pars cochlearis: maximum transverse diameter	43	58

**Note:**

Measurements of the periotics of *Nehalaennia devossi* (NMR 999100014035, holotype). Data in mm.

The anterior process is triangular and flat (Fig. 16C). The lateral tuberosity (sensu Ekdale, Berta & Deméré, 2011) protrudes laterally in a remarkable manner and has a triangular outline. The anterior border of the lateral tuberosity is continuous with the lateral border of the anterior process. The lateral border of the anterior process is straight, the medial border is medially convex. The anterior end is triangular and pointed. The anterior pedicle for the attachment of the tympanic bulla is located only a few mm anteriorly to the lateral tuberosity; it is rectangular in shape, narrow and runs for 24 mm (on the left side) paralleling the lateral border of the anterior process. Posteromedially, a robust structure connects the anterior process and the pars cochlearis. That structure represents the medial border of a complex system of grooves developed in the ventral surface of the anterior process that is described as follows (Fig. 16C). A groove starts from the anterolateral corner of the pars cochlearis, it runs paralleling the medial border of the anterior process up to half the length of the anterior process; its starting point is located at the location of the fossa incudes. Once this groove has reached the middle of the anterior process, it is confluent into a small fossa; from that fossa, two grooves depart: one projects medially and forms the anterior limit of the posteromedial emergence of the anterior process, the other runs to up to the apex of the anterior process.

The pars cochlearis is elongated along both the anteroposterior and transverse axes. Its ventral surface is slightly rounded. The groove for the tensor tympani muscle is wide and long. There is no trace of any median promontorial groove. The round window is open and confluent into the aperture for the cochlear aqueduct.

Only a small part of the dorsal surface of the periotic can be observed in the right periotic. Based on that, it is evident that the anterior process is mainly flat dorsoventrally and, dorsally to the pars cochlearis, the dorsal surface of the periotic forms a high and bulbous projection (Fig. 15D) with a dorsally convex outline.

**Figure 17:**
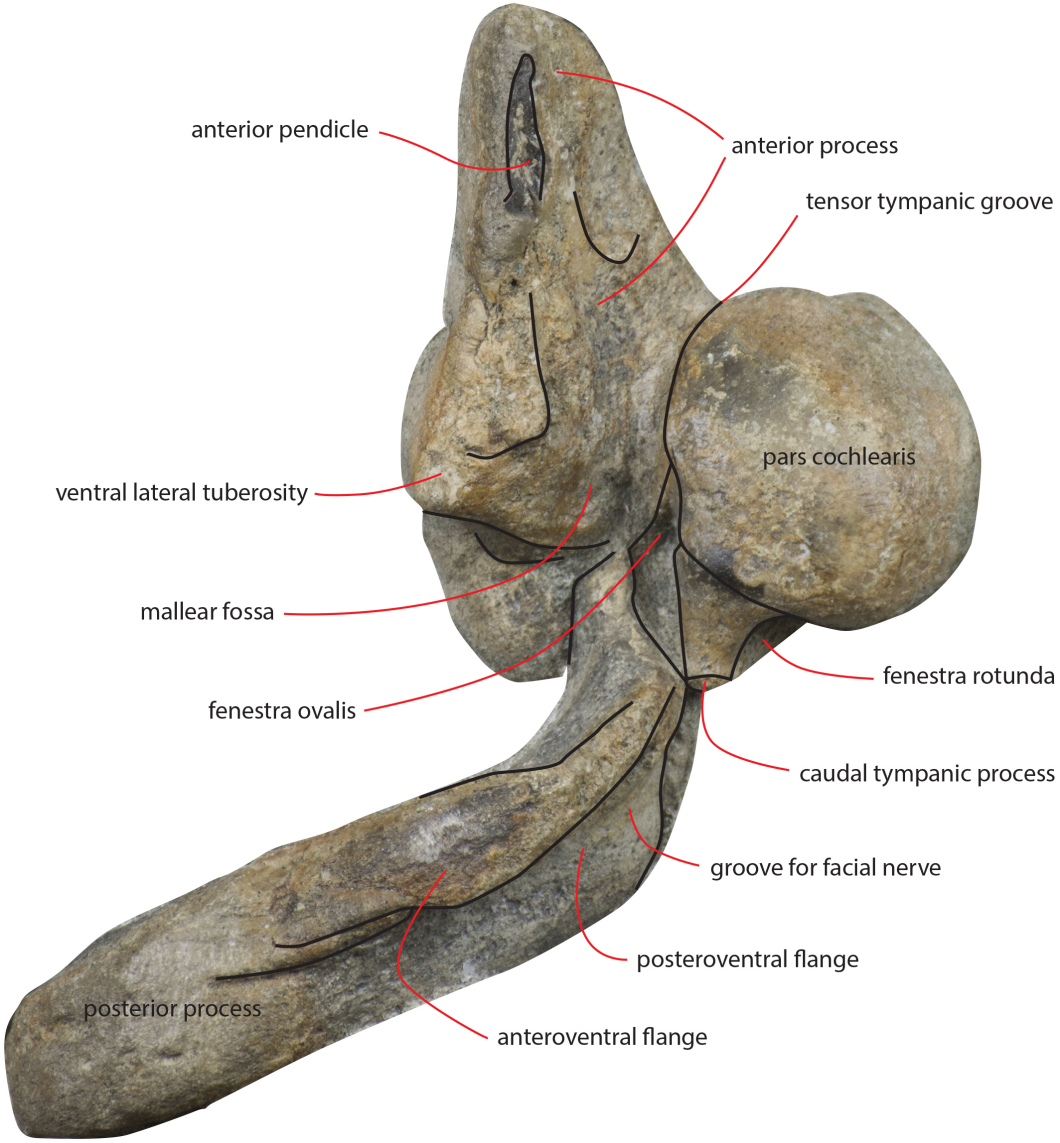
Periotic in ventral view. Right periotic of the holotype of *Nehalaennia devossi* (NMR 9991000 14035) in ventral view.

The caudal tympanic process is large and long (Fig. 17). It projects posteriorly and forms the border of a foramen for the transit of the facial nerve linked to the groove located along the ventral surface of the posterior process of the periotic.

The oval window is difficult to observe (Fig. 16E). However, it is clearly evident that the oval window is separated from the fossa for the stapedial muscle by the interposition of a crest. Such a fossa has an oval outline. An additional crest separates the oval window from the endocranial (i.e., internal) opening of the facial canal.

### Atlas

**Figure 18:**
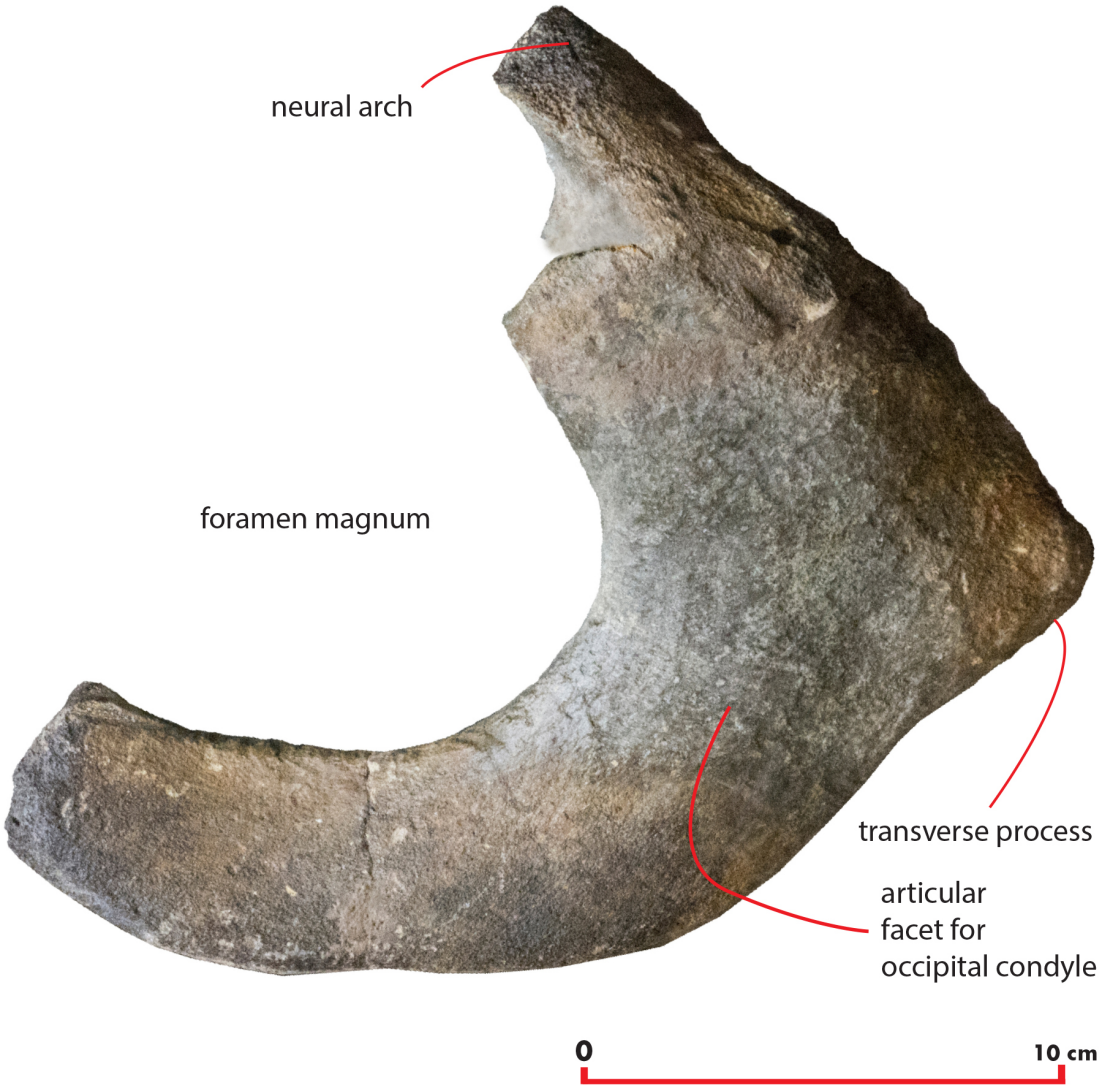
Atlas. Morphology of the atlas of the holotype skull of *Nehalaennia devossi*. The atlas was digitally isolated from the remainder of the skull.

A fragment of the atlas is in close connection with the skull as it is disarticulated from its original position and moved laterally and anteriorly along the left side (Fig. 18). The atlas is broken along the dorsoventral axis so that only half of it is still preserved. The ventral surface is thin and flat (Table 6). The lateroventral corner of the vertebra is evident but delicate. The articular facet for the second cervical vertebra is posteriorly convex and transversely elongated. Only a small fragment of the neural arch is preserved that is subtle and delicate suggesting that the individual was not fully grown. The foramen magnum is wide both dorsoventrally and transversely.

**Table 6:** Measurements of the atlas.

Character	Measurement
Maximum height	140
Maximum width	220
Height of foramen	105
Width of ventral border	75
Width of dorsolateral border	115

**Note:**

Measurements of the atlas of *Nehalaennia devossi* (NMR 999100014035, holotype). Data in mm.

## Comparisons

*Nehalaennia devossi* shows a surprisingly modern morphology for a whale of its stratigraphic age. The shape of the anterior border of the supraoccipital together with most of the other cranial details strikingly support the hypothesis that this specimen represents a close relative of modern balaenopterid whales of the genera *Balaenoptera* and *Megaptera*. As stated in the differential diagnosis section, *Nehalaennia devossi* exhibits a number of different characters with respect to the Pliocene and Miocene records of Balaenopteridae. In particular, most of fossil balaenopterid species share the presence of a transversely constricted anterior portion of the supraoccipital with a transversely short anterior border of the supraoccipital. Based on our observations, living balaenopterid species show a transverse expansion of the postorbital constriction and a consequent increase in the transverse diameter of the anterior portion of the supraoccipital posteriorly to the interorbital region of the frontal. *Nehalaennia devossi* shares this character with modern balaenopterid species and, in that, it differs from the transversely narrow anterior border of supraoccipital that is observed in Pliocene and Miocene balaenopterid species (with the exception of *‘Megaptera’ miocaena* that has a transversely anterior border of the supraoccipital).

Gol’din & Steeman (2015) described the supraoccipital of *Tranatocetus argillarius* and stated that it shows what seems a distinctive character: the supraoccipital, in this species, is bent and changes its orientation approximately at mid-length. This means that the posterior-most portion of the supraoccipital is more vertically-oriented and the anterior-most portion is more horizontally-oriented. Gol’din & Steeman (2015) stated that this characters may be used to support the monophyly of Tranatocetidae. Unfortunately, a bent supraoccipital is observed also in fossil balaenopterid species. In particular, this character is evident in *Archaebalaenoptera castriarquati* and *N. devossi* thus suggesting that it may not be used as an unambiguous evidence in support for Tranatocetidae.

The shape of the glenoid fossa of the squamosal observed in *N. devossi* represents an additional evidence of its close affinity with modern balaenopterid species. In most of the Miocene and Pliocene balaenopterids the glenoid fossa of the squamosal is not highly concave as in modern balaenopterids. The concavity of this fossa is slight when the skull is observed in lateral view in *‘Balaenoptera’ cortesi* var. *portisi*, *‘Megaptera’ hubachi*, *‘Balaenoptera’ bertae*, *Incakujira anillodefuego* and *Fragilicetus velponi*. In *Archaebalaenoptera castriarquati* the glenoid fossa of the squamosal is straight in lateral view. In these species, the slight concavity or the straight profile of the glenoid fossa of the squamosal depends upon a posterior or posteroventral orientation of the postglenoid process of the squamosal; such a character is absent in modern balaenopterid species and *N. devossi* where the postglenoid process of the squamosal projects ventrally in a more marked way.

The rostrum of *N. devossi* shows the typical balaenopterid characters in the proportions and shapes of the ascending process of the maxilla, the position of the nasofrontal suture, and the interdigitation between maxilla, frontal and parietal (Kellogg, 1928; Miller, 1923).

The ‘primary dorsal infraorbital foramen’ was described and discussed in detail by Marx, Bosselaers & Louwye (2016) with focus on Cetotheriidae. These authors stated that the foramen is located at the base of the ascending process of the maxilla and is prolonged into a sulcus running posterodorsally. Such a pattern is rarely observed outside the Cetotheriidae but *N. devossi* shows it clearly. In particular, the foramen is prolonged into a concavity that occupies most of the dorsal surface of the ascending process of the maxilla that resembles very closely the sulcus described by Marx, Bosselaers & Louwye (2016). If these authors are right in suggesting that the ‘primary infraorbital foramen’ and the associated sulci are related to a peculiar vascularization pattern of the skull, then such a pattern should be expected to have been present also in *N. devossi*. However, we suggest caution when naming peculiar dorsal infraorbital foramina because intraspecific variation has been detected about the distribution of this structure in the balaenopterid rostrum (Haberland et al., 2018).

Our reconstruction of the temporal fossa of *N. devossi* supports the hypothesis that the in this species there was not any lateral exposure of the alisphenoid. Among living balaenopterid species, according to Fraser & Purves (1960), the lack of lateral exposure of the alisphenoid in the temporal fossa is observed in *M. novaeangliae* only. As far as the fossil record is concerned, it is hard to get this information as in many specimens the temporal fossa is not well preserved or is still obliterated by the matrix. In particular, the alisphenoid is exposed in the temporal fossa in *‘Megaptera’ hubachi*, *Fragilicetus velponi*, *Incakujira anillodefuego*, *Diunatans luctoretemergo*, and in all the living species of the genus *Balaenoptera*. It is hard to state if the alisphenoid is exposed in the temporal fossa in the fossil *Archaebalaenoptera castriarquati* because the sutures present in the temporal fossa are hardly seen; the lateral view of the skull of *Protororqualus cuvieri* is not known thus it is not possible to state whether the alisphenoid was exposed in this species or not. The temporal fossa is not preserved in *Plesiobalaenoptera quarantellii* and is preserved only in part in *‘Balaenoptera’ bertae* therefore, unfortunately, this prevents the observation of the sutural pattern of this region showing the presence of the alisphenoid or not. Interestingly, according to Kellogg (1922) the alisphenoid is not observed in *‘Megaptera’ miocaena*, a species that some authors link to the living humpback whale, *M. novaeangliae* (Kellogg, 1922; Boessenecker, 2013; Boessenecker & Fordyce, 2015a, 2015b).

The presence of a wide anterolateral concavity along the anterior border of the supraorbital process of the frontal is observed in the living *M. novaeangliae*. Both the anterolateral concavity in the anterior border and the straight posterior border of the supraorbital process of the frontal are observed in *N. devossi*.

## Phylogenetic Analysis

### Mysticete phylogeny

The results of our analyses are shown in Table S2 in the Supplemental Information. As shown, the most parsimonious solutions were found by the simultaneous use of Tree Fusing, Drift and Ratchet algorithms implemented in the New Technology search method of TNT setting off the Sectorial Search. All the other combinations of weighted and unweighted analyses provided less parsimonious solutions. The remainder of the present paper is thus based on the most parsimonious cladograms found by using the New Technology Search method.

**Figure 19:**
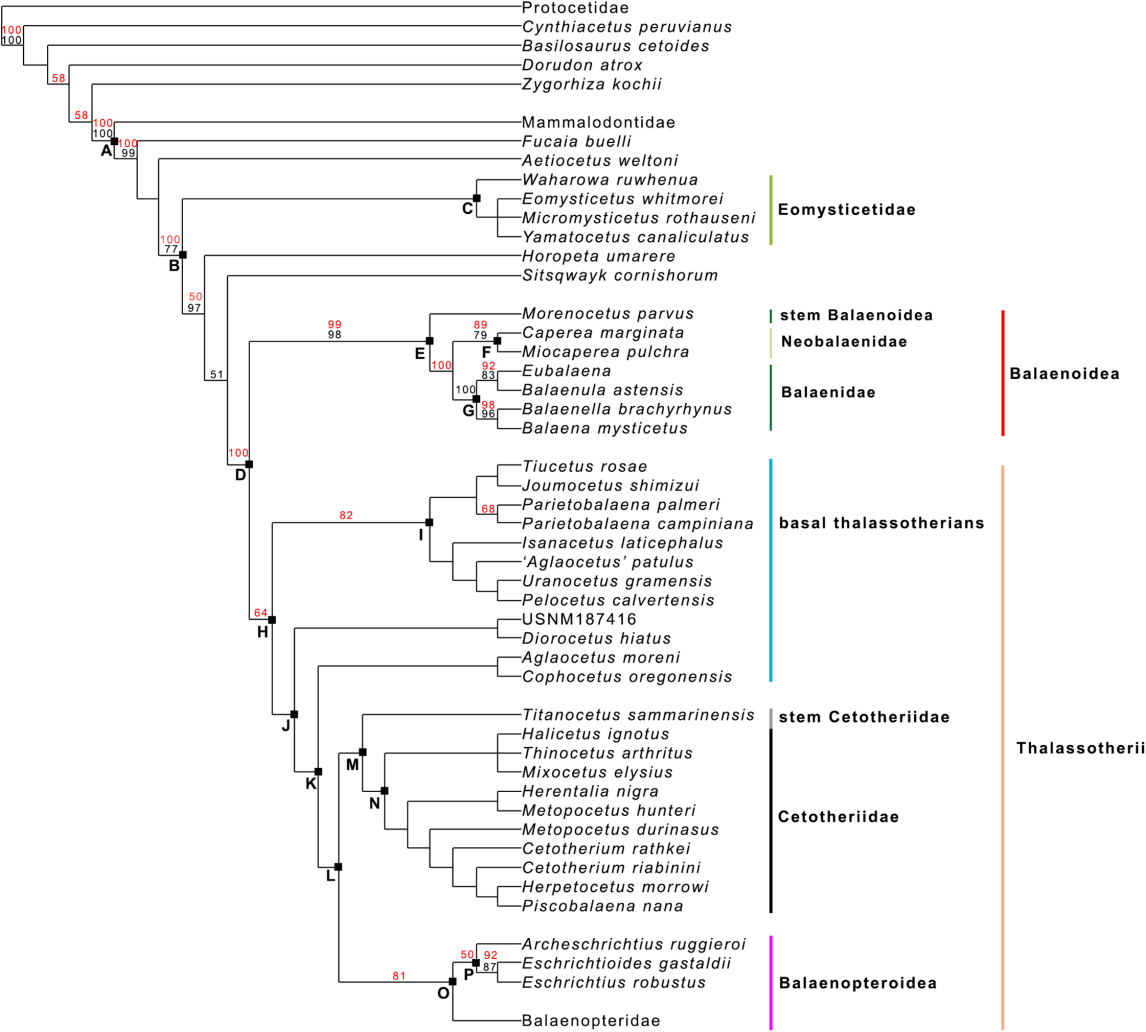
General features of the phylogeny of mysticetes. Strict (Nelsen) consensus of two equally parsimonious trees showing the phylogenetic relationships of selected mysticete taxa. The tree is 1,604 steps in length and has a Consistency Index (CI) of 0.291 and a Retention Index (RI) of 0.756 (both indexes are calculated by TNT). The Homoplasy Index (HI = 1−CI) is 0.709 and the Rescaled Consistency Index (RC = CI × RI) is 0.219. Black numbers above the branches represent bootstrap support values, red numbers represent branch support values obtained from symmetrical resampling. Previously named clades corresponding to clade letters are the following: A, Mysticeti; B, Chaeomysticeti; C, Eomysticetidae; D, Balaenomorpha; E, Balaenoidea; F, Neobalaenidae; G, Balaenidae; H, Thalassotherii; I, basal thalassotherian taxa; M, stem Cetotheriidae; N, Cetotheriidae; O, Balaenopteroidea; P, Eschrichtiidae. Other letters (J, K and L) are unnamed clades. Intra-family relationships of Balaenopteridae are shown in Fig. 20.

**Figure 20:**
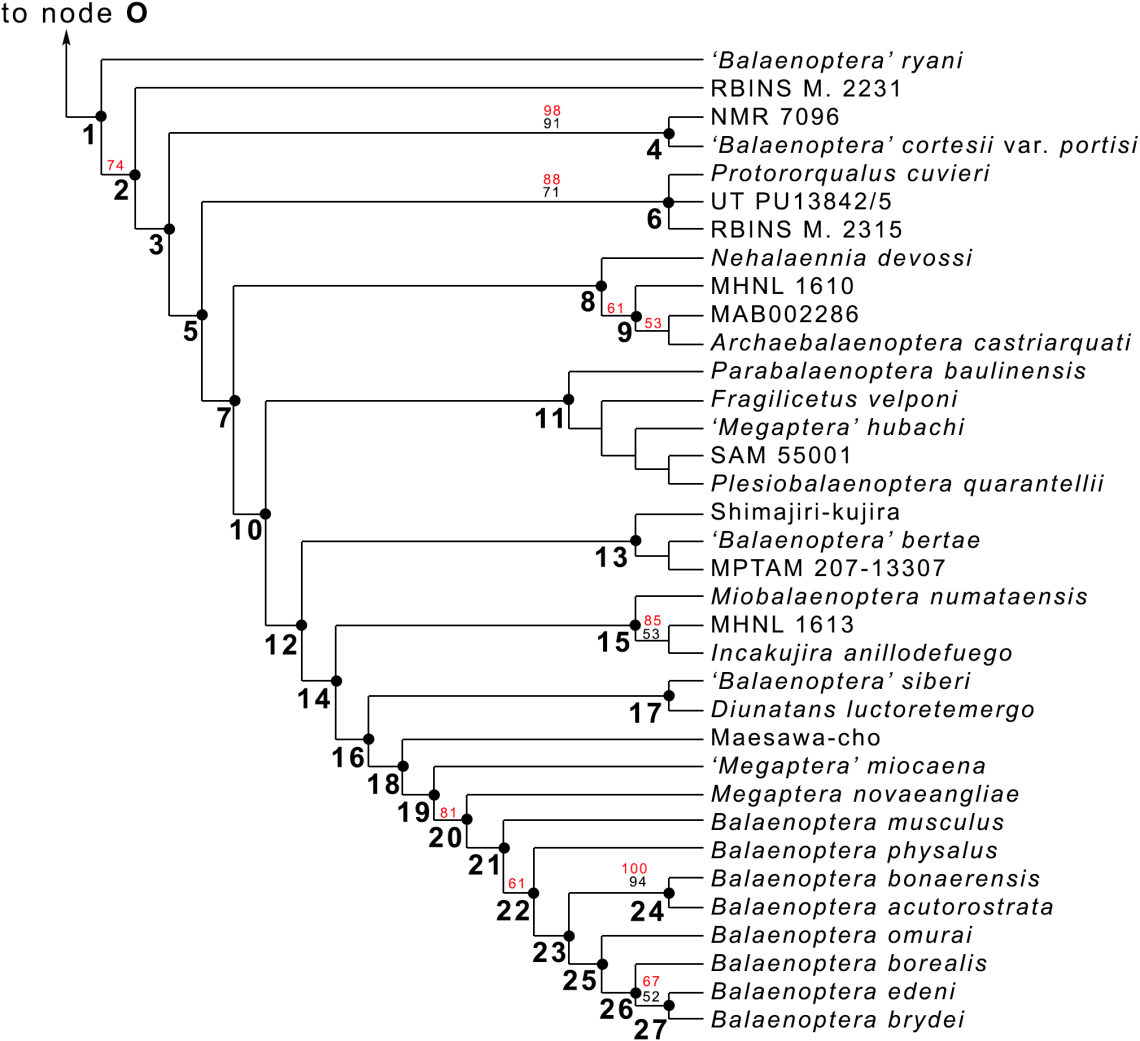
Phylogeny of Balaenopteridae. Expansion of phylogenetic relationships of Balaenopteridae that are not shown in Fig. 18. Letters at nodes represent major balaenopterid clades discussed in the text. Previously named clades corresponding to node numbers are the following: 1, Balaenopteridae; 20, crown balaenopterids; 21, *Balaenoptera*. Other clades are discussed in the text.

Our phylogenetic analysis resulted in two equally parsimonious cladograms (1,604 steps in length) whose strict consensus (Nelsen) tree is shown in Fig. 19 (cladogram statistics are presented in the corresponding caption). The phylogenetic relationships within Balaenopteridae are showed in Fig. 20. Our morphological data support the monophyly of Mysticeti (node A in Fig. 19) with a bootstrap support value (BSV) and symmetric resampling support value (SRV) of 100%. Highly supported clades include also *Aetiocetus weltoni* + Chaeomysticeti (node B: BSV = 77%; SRV = 100%), Chaeomysticeti (node D: BSV = 98%; SRV = 100%), Balaenomorpha (SRV = 100%), Balaenoidea (node E: BSV = 98%; SRV = 99%), Balaenidae (BSV = 100%), Neobalaenidae (BSV = 79%; SRV = 89%), and Balaenopteroidea (SRV = 81%). Most of the other clades received BSV and SRV less than 50%. We interpret the lack of higher support at the other nodes as a sign of high homoplasy in the dataset. It is also possible that the low support values are due to a large amount of unknown (? States in the matrix) character states for fossil taxa.

Interestingly, from our results, a clade exists including a number of basal thalassotherian taxa that incorporates *Tiucetus, Joumocetus, Parietobalaena, ‘Aglatocetus’ patulus, Uranocetus, Pelocetus* and *Isanacetus*. This clade does not include all the basal thalassotherian taxa as four genera branch out from it at positions that are closer to Cetotheriidae. In particular, *Aglaocetus patulus, Diorocetus hiatus,* USNM 187416 and *Cophocetus oregonensis* form a sequence of sister groups of the clade including both Cetotheriidae and Balaenopteroidea.

From our results, thus, Thalassotherii (node H in Fig. 19) is formed by three large, epifamily-rank clades respectively branching from nodes I, M and O. The first one (node I) includes a large group of basal thalassotherian taxa characterized by wide rostrum at its base. The second group (node M) includes Cetotheriidae and the third group (node O) includes the Balaenopteroidea. Stem groups are also observed as one species of stem cetotheriid (*Titanocetus sammarinensis*) and two subsequent stem Balaenopteridae + Cetotheriidae taxa exist.

Continuous ram feeder mysticetes form a single, monophyletic clade, namely Balaenoidea (node E). This includes both Balaenidae and Neobalaenidae. Morphological support includes the presence of fuzed cervical vertebrae, anterior thrust of the supraoccipital that is superimposed on the parietal and on the posterior portion of the infraorbital region of the frontal, reduced conical process in the tympanic bulla, low tympanic cavity, articular surface of the mandibular condyle faced dorsally with respect to the long axis of the dentary, reduced coronoid process in the dentary, well developed mylohyoidal groove in the dentary.

Taxa branching from node O (Balaenopteroidea) share elongated and narrow ascending process of the maxilla and nearly flat supraorbital process of the frontal. Balaenopteroidea share the presence of a transversely and anteroposteriorly elongated pars cochlearis in the periotic. The morphological support of Balaenopteridae includes characters described and discussed by Bisconti & Bosselaers (2016).

### Phylogenetic relationships within Balaenopteridae

From our results, in the monophyletic Balaenopteridae, the basal-most balaenopterid taxon is represented by *‘Balaenoptera’ ryani* (Fig. 20). This, in its turn, is the sister group of a clade whose earliest diverging branch is represented by a specimen whose formal description is currently under preparation by one of the authors (MB) and that is here identified as RBINS M. 2331 (see Bisconti & Bosselaers (2016) for a general introduction to the morphology of this taxon that they called Belgium 1). Subsequent branching patterns observed within Balaenopteridae allow to identify a number of monophyletic groups. RBINS M. 2331 is the sister group of a clade (node 4 in Fig. 20) that includes*‘Balaenoptera’ cortesi* var. *portisi* and NMR 7096. In its turn, this clade is the sister group of the genus *Protororqualus* that is here represented by *Protororqualus cuvieri*, UT PU13842/5 and RBINS M. 2315. The genera *Archaebalaenoptera* and *Nehalaennia* form a monophyletic group that is the sister group of clades branching from node 10. Earliest diverging rami of node 10 include *Plesiobalaenoptera quarantellii* and SAM55001 supporting an early interpretation of the latter as belonging to the genus *Plesiobalaenoptera* (Govender, Bisconti & Chinsamy, 2016). Interestingly, *‘Megaptera’ hubachi* is the sister group of *Plesiobalaenoptera*, *Fragilicetus* is the sister group of *‘M.’ hubachi* and *Parabalaenoptera* is he sister group of *Fragilicetus*. Branching from node 12, three large clades form a sequence of sister groups including such genera as *Miobalaenoptera*, *Incakujira*, *‘Balaenoptera’ bertae*, and crown balaenopterids.

*‘Balaenoptera’ siberi* and *Diunatans luctoretemergo* represent the sister group of a clade including all living balaenopterids and a small number of fossil species characterized by modern morphologies. These include the specimen from Maesawa-cho, and *‘Megaptera’ miocaena*. The latter is the sister group of the crown balaenopterids including the genera *Balaenoptera* and *Megaptera*.

Crown balaenopterids branch from the node 19 and include *M. novaeangliae* that is the sister group of genus *Balaenoptera*. *Balaenoptera* is a monophyletic genus including two basal species (*Balaenoptera musculus* and *Balaenoptera physalus*) and two clades that comprehend (1) *Balaenoptera acutorostrata + Balaenoptera bonaerensis* and (2) a group in which *Balaenoptera omurai* is basal to a clade comprising *Balaenoptera borealis*, *Balaenoptera edeni* and *Balaenoptera brydei*.

Support values for balaenopterid clades are generally <50% and are not presented in Fig. 20. The only relationships that are highly supported include: (a) the clade comprising *Protororqualus cuvieri*, UT PU13842/5 and RBINS M. 2315 that could reasonably belong to the same genus (i.e., *Protororqualus*) with a BSV of 71% and a SRV of 88%; (b) the clade including *‘Balaenoptera’ cortesii* var. *portisi* and NMR 7096 with a BSV of 91% and a SRV of 98%; (c) the clade comprising MHNL 1613 and *Incakujira anillodefuego* that should belong to the same species being supported by a BSV of 53% and a SRV of 85%; (d) crown balaenopterids including *M. novaeangliae* and the living *Balaenoptera* species that is supported by a SRV of 81%.

### Assessing the stratigraphic coherence of the branching pattern

**Figure 21:**
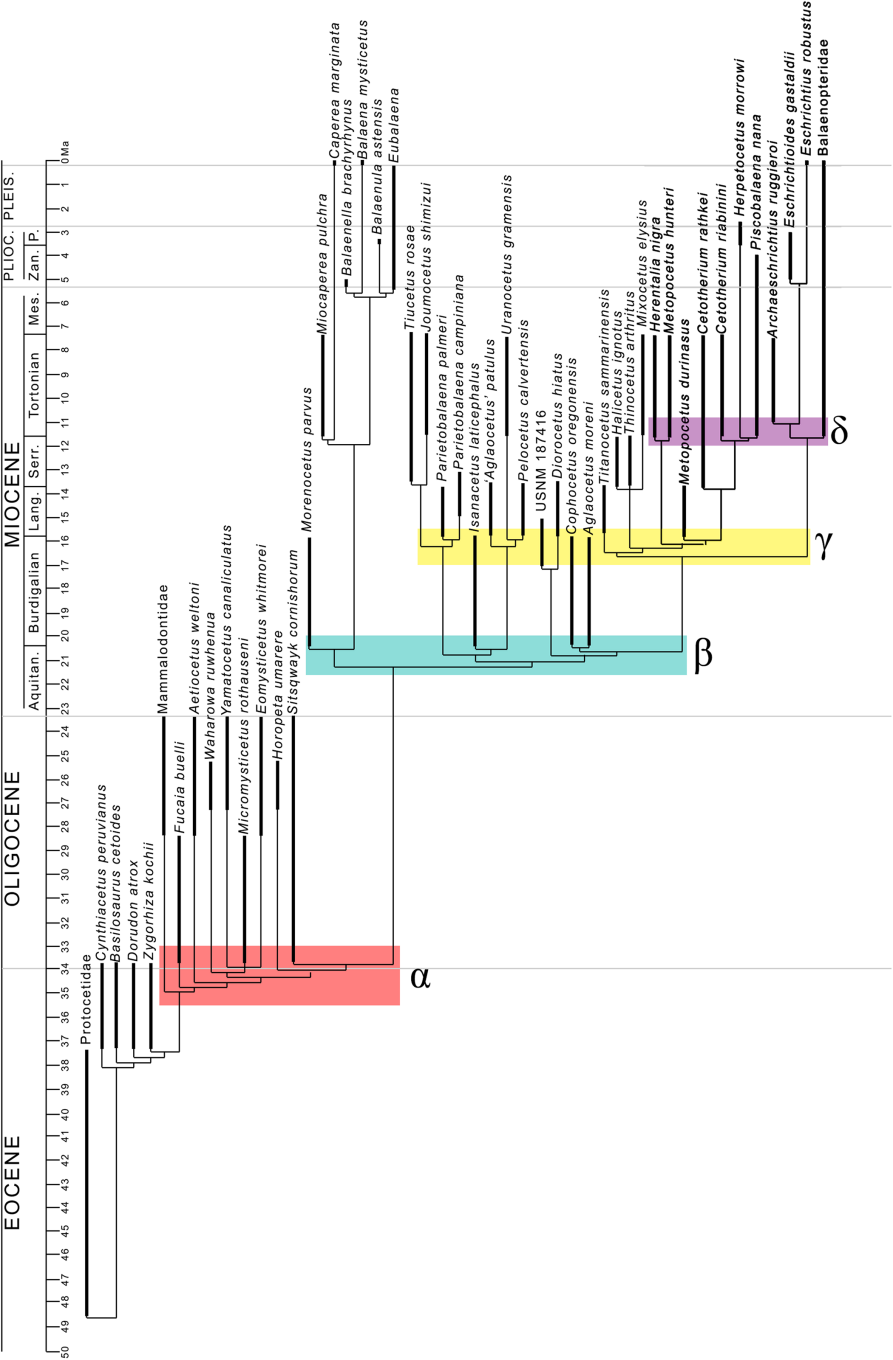
Phylogeny of Mysticeti against temporal scale. Phylogenetic relationships of Mysticeti based on the results shown in Fig. 18 plotted against the temporal scale showing major events of diversification and extinction. Abbreviations: Ma, million years; Aquitan., Aquitanian; Lang., Langhian; Mes, Messinian; P., Piacenzian; Pleis., Pleistocene; Plioc., Pliocene; Serr., Serravallian; Zan., Zanclean. Bold lines represent stratigraphic ages of the taxa (see Supplementary Information for details); thin lines represent inferred lineage durations based on the hypothesis of phylogeny presented in this work.

The SCI of the whole hypothesis of phylogeny is obtained by dividing the number of stratigraphically consistent nodes (that is 44) against the total number of nodes (given by the total number of taxa minus 2: 82 – 2 = 80). From these calculations, the SCI for the whole dataset is 0.65. The balaenopterid partition of the dataset is particularly at odd with this observation because only a small number of balaenopterid nodes are stratigraphically consistent (14 from a total of 33); for this reason, the SCI for the balaenopterid partition is 14/33 = 0.42. On the contrary, the SCI calculated for all non-balaenopterid mysticetes given by 44/80 = 0.55. The relationships between the branching order of mysticete clades and the stratigraphic ages are shown in Fig. 21. As shown, most of the mysticete branches are from the Miocene.

**Figure 22:**
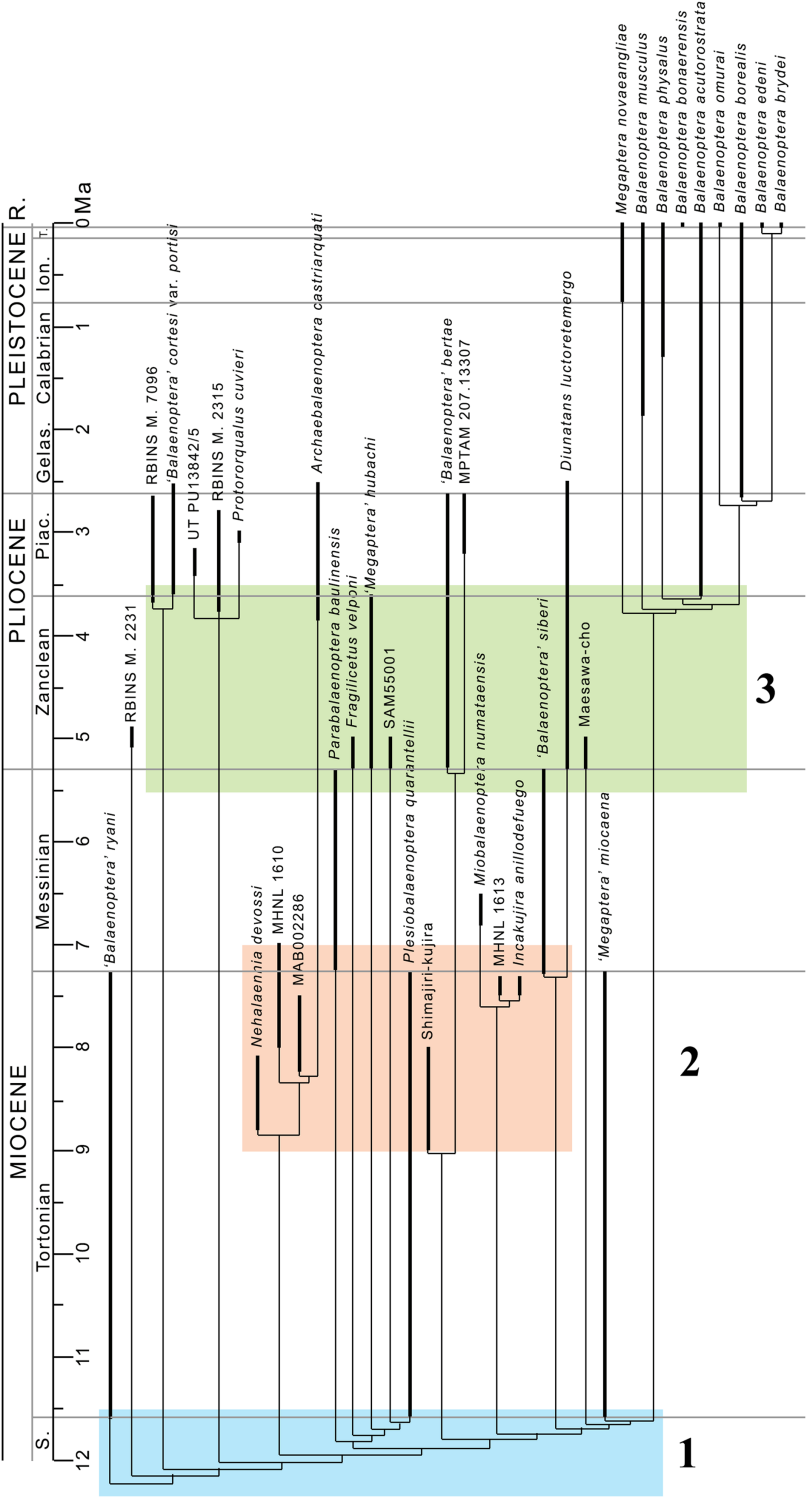
Phylogeny of Balaenopteridae against temporal scale. Phylogenetic relaltionships of Balaenopteridae based on the results shown in Fig. 20 and plotted against the temporal scale showing major events of balaenopterid diversification and extinction. Abbreviations: Ma, million years; Messin., Messinian; Piac., Piacenzian; Serravall., Serravallian; Zancl., Zanclean.

In Fig. 22, the relationships between the branching order of the balaenopterid taxa and their stratigraphic ages is also shown suggesting that a high number of ghost taxa has to be hypothesized in order to interpret the present phylogenetic hypothesis. This is probably the reason of the particularly low SCI calculated above. From our hypothesis, most of the balaenopterid radiations must be initiated before the Tortonian including the origin of the lineage leading to the crown balaenopterids. Living balaenopterids seem to have been originated in the Pliocene from a lineage whose history started in the early Tortonian. Unfortunately, presently, it is not possible to break the long ramus leading to the extant *Balaenoptera* and *Megaptera*.

The clade including *Nehalaennia* and *Archaebalaenoptera castriarquati* originated from the mid-Tortonian but has a longer evolutionary history. Interestingly, the late Pliocene occurrence of the last member of this clade shows that long gaps exist even in this small species group.

### Detecting evolutionary radiations

**Figure 23:**
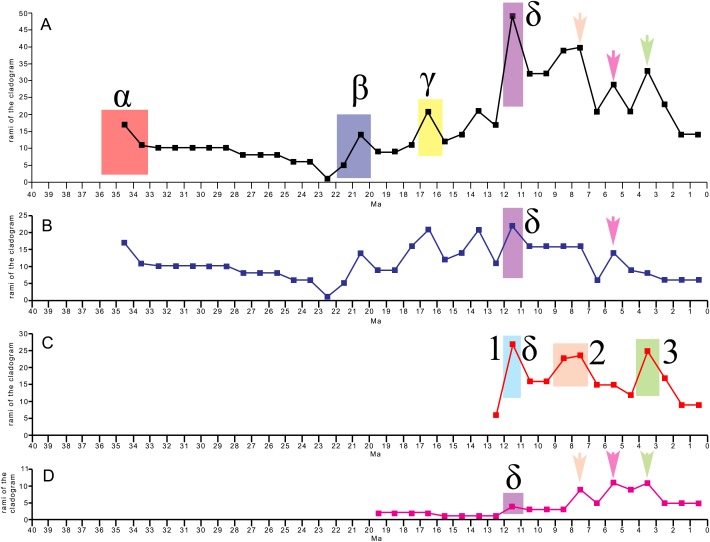
Clade diversity of baleen-bearing mysticetes through time. Plots showing clade diversity through time starting from the origin of Chaeomysticeti. (A) Clade diversity of Chaeomysticeti. (B) Clade diversity in Chaeomysticeti without Balaenopteridae. (C) Clade diversity in Balaenopteridae. (D) Clade diversity in Balaenidae (data from Bisconti, Lambert & Bosselaers, 2017). Numbers, arrows, Latin and Greek letters refer to events discussed in the text. Ma, million years ago.

The clade diversity of Balaenomorpha is presented in Table S2 in the Supplemental Information File. In Fig. 23 we show the results of our investigation about the occurrence of radiations within Balaenopteridae in the broader context of the evolution of balaenomorph diversity. It is evident that three main radiations can be observed based on the cladogram of Fig. 21. The Event α is given by the first appearance of toothed and baleen-bearing Mysticeti but does not include balaenomorph mysticetes. This radiation occurred between *c.* 36 and 33 Ma. The Event β depended upon a burst in early thalassotherian diversification occurred between *c.* 22 and 20 Ma. The Event γ was triggered by an additional increase in thalassotherian diversity with the exclusion of Balaenopteridae (that were still not existent) and occurred between *c.* 17 and 16 Ma. The Event δ depended upon a third pulse in thalassotherian diversity and upon the early radiation of Balaenopteridae as it coincides with the Event 1 of Balaenopteridae. A small contribution to this radiation was also provided by an increase in balaenoid diversity between *c.* 12 and 10 Ma (origin of *Miocaperea pulchra* and the lineage leading to the living *Caperea marginata*). The Event 2 of Balaenopteridae is not linked to other thalassotherians but is connected to an increase in clade diversity of Balaenoidea (pink arrow). This radiation occurred between nine and seven Ma. The Event 3 of Balaenopteridae occurred between *c.* five and three Ma and is coincident with an additional diversification event of Balaenoidea (green arrow). The purple arrow indicates a diversity increase in Balaenoidea occurring between *c.* six and five Ma and does not coincide with diversity peaks of other clades.

## Discussion

### Phylogenetic relationships of Chaeomysticeti

The phylogenetic hypothesis presented in the present paper about mysticetes, shows several points that have to be discussed bearing in mind that discussions about the early radiation of chaeomysticetes are outside the scope of the present article. The first point is about the general structure of the chaeomysticete branching pattern. From our results, Chaeomysticeti is subdivided into two main clades, that is, Eomysticetidae (branching from node B in Fig. 19) and Balaenomorpha (node D in Fig. 19). This pattern is confirmed also by other works (Boessenecker & Fordyce, 2015a, 2015b, 2016; Peredo et al., 2018; Bisconti, 2012, 2015; Bisconti & Bosselaers, 2016; Bisconti, Lambert & Bosselaers, 2013, 2017; Sanders & Barnes, 2002). In its turn, Balaenomorpha is subdivided into two superfamily-rank clades: (1) Balaenoidea (node E in Fig. 19) and Thalassotherii (node H in Fig. 19). This pattern conflicts with recent works suggesting that Balaenoidea does not exist because one of its components (namely, Neobalaenidae) is part of Cetotheriidae (Marx & Fordyce, 2016; Fordyce & Marx, 2012). We are unable to confirm this hypothesis based on our dataset; in this respect, our results agree with those provided by other workers using our and different morphological datasets (Peredo et al., 2018; Boessenecker & Fordyce, 2015a, 2015b, 2016; El Adli, Deméré & Boessenecker, 2014; Bisconti & Bosselaers, 2016; Bisconti, Lambert & Bosselaers, 2013, 2017; Bisconti, 2012).

As a general discussion, it is true that there is a big discrepancy between morphological and molecular results. This is also true in other works. Compare, for instance, the morphological vs. total evidence results in Geisler et al. (2011) and Deméré, Berta & McGowen (2005). Moreover, results by Boessenecker & Fordyce (2015b) and Peredo & Uhen (2016) differ from molecular results in the position of *Caperea*. We admit that the discrepancies between morphological and molecular reconstructions represent a major problem in cetacean phylogenetics that requires a major effort to be resolved. We do not think that our paper should be designed to resolve this big problem because we feel that a dedicated research program should be devoted to this and such a program is outside the scope of the present manuscript. In general, however, it is well known that molecular phylogenetics suffers of its own problems especially when the group under study includes high numbers of extinct species (Heath et al., 2008; Wagner, 2000). Dedicated works show, in fact, that when groups formed by a majority of extinct taxa are studied, morphological phylogenetics is more likely to provide more accurate results that better approximate the true phylogenetic history (Wagner, 2000). This is exactly the case of Balaenopteridae that includes only a few living taxa and a huge number of extinct taxa.

One of the most interesting results of the present phylogenetic analysis is the full resolution of the relationships of basal thalassotherian taxa; in previous works based on earlier versions of this dataset, the basal thalassotherian taxa formed an unresolved polytomy (Bisconti, Lambert & Bosselaers, 2013; Bisconti & Bosselaers, 2016). By the present analysis we were able to resolve the relationships of the basal thalassotherian taxa that are now subdivided into well resolved groups: (1) a monophyletic group (node I in Fig. 19) including broad-rostrum, narrow-nasal taxa (2) a paraphyletic group of stem-cetotheriids (nodes J and K in Fig. 19) with pyruliform tympanic bulla and endocranial opening of the facial canal widely separated from the internal acoustic meatus. It is interesting to note that transverse reduction of the rostrum, lowering of the dorsal surface of the periotic and elongation of ascending process of the maxilla occur in the taxa branching from nodes J and K according to their branching order; in fact, in the more basal stem thalassotherian taxa (*Joumocetus* and *Parietobalaena*) the rostrum is wider at its base, the dorsal surface of the periotic is raised and the ascending process of the maxilla is short; in more advanced forms, such as *Diorocetus* and USNM 187416, the rostrum is transversely narrow, the ascending process of the maxilla tends to converge toward the long axis of the skull and the dorsal surface of the periotic is not raised.

Support for this hypothesis is also provided by a number of previously published works: Kimura & Hasegawa (2010) proposed that *Joumocetus* and Cetotheriidae form a monophyletic group; they suggested that *Joumocetus* is a Cetotheriidae but we cannot confirm this statement based on our dataset. Boessenecker & Fordyce (2015a, 2015b, 2016) suggested that the basal thalassotherian taxa formed a monophyletic group but in their hypothesis, also *Titanocetus*, *Cophocetus*, *Aglaocetus moreni* and *Diorocetus* were part of such a clade. Marx & Kohno (2016) and Tanaka, Ando & Sawamura (2018) confirmed the close affinity of *Diorocetus hiatus* and Cetotheriidae and found a monophyletic group including most of the basal thalassotherian taxa but also including *Parietobalaena* and *Joumocetus*. Peredo & Uhen (2016) and Peredo et al. (2018) found also a monophyletic group including most of the basal thalassotherian taxa but such a group was the sister group of Balaenopteroidea being Cetotheriidae placed at a more basal position. This discussion underlines that results about the relationships of the basal thalassotherian taxa are still not shared by all the authors; however, in the recent literature, there is a general tendency to consider the basal thalassotherian taxa as forming a monophyletic group closely related to Cetotheriidae and Balaenopteroidea (Boessenecker & Fordyce, 2015a, 2016; Peredo & Uhen, 2016; Peredo et al., 2018). This tendency is in sharp contrast with publications from the early 2000s where basal thalassotherian taxa (e.g., cetotheres *sensu* Kellogg, 1965, 1968, 1969) and Cetotheriidae were considered a taxonomic ‘wastebasket’ (Fordyce & De Muizon, 2001; Kimura & Ozawa, 2002). Debate is still going on about the precise placements of some taxa. Noteworthy exceptions to this interpretation are the results of Gol’din & Steeman (2015) and Gol’din (2018) where the basal thalassotherian taxa are splitted across several branches in the Balaenomorpha clade and do not form any monophyletic group.

Our results support the monophyly of Balaenopteroidea and its subdivision into two family-rank clades: (1) Eschrichtiidae and (2) Balaenopteridae. This result conflicts with most of the molecule-based hypotheses where the living *Eschrichtius robustus* is placed within Balaenopteridae (Gatesy et al., 2013; Hatch, Dopman & Harrison, 2006; May-Collado & Agnarsson, 2006). Also, some recent total evidence studies agree that *Eschrichtius* is to be placed inside Balaenopteridae thus making Eschrichtiidae a non-existent family (Slater, Goldbogen & Pyenson, 2017; Marx, Bosselaers & Louwye, 2016). From a morphological view, the inclusion of *Eschrichtius* within Balaenopteridae is difficult to accept. *Eschrichtius* and the fossil eschrichtiid *Eschrichtioides* and *Gricetoides* share characters that are much more primitive than those observed in the living balaenopterid genera. In some respects, eschrichtiids exhibit morphologies that are intermediate between Balaenopteridae and Cetotheriidae as they are characterized by an intermixing of cetotheriid (protruding posterolateral corner of the exoccipital, temporal crest at vertex formed by two distinct lateral concavities, triangular and stocky zygomatic process of the squamosal) and balaenopterid (depressed supraorbital process of the frontal, transversely elongated pars cochlearis in the periotic) characters. As shown by Bisconti & Bosselaers (2016) the sister group relationship of Eschrichtiidae and Balaenopteridae is clearly evident by a consistent distribution of synapomorphies across their phylogenetic hypothesis that, concerning this point, is the same provided in Fig. 19 of the present paper. We, therefore, conclude that Eschrichtiidae is an existent family and that it is the sister group of Balaenopteridae. Additional morphological support to this hypothesis is provided by Peredo et al. (2018), Peredo & Uhen (2016), Boessenecker & Fordyce (2016, 2015a, 2015b), El Adli, Deméré & Boessenecker (2014) and Deméré, Berta & McGowen (2005). Future studies will undoubtly proof where and why this conflict between our morphological model and molecule-based models occurs.

### Phylogenetic relationships of Balaenopteridae

The hypothesis of balaenopterid phylogeny proposed in the present paper (Fig. 20) suggest that several radiations occurred after the origin of the family. Morphologically archaic taxa form clades from 1 to 15 in Fig. 20, and include taxa that have been already considered primitive by different authors. In fact, for example, *Protororqualus cuvieri* and *Parabalaenoptera baulinensis* were considered the most primitive balaenopterids by Zeigler, Chan & Barnes (1997); *Fragilicetus velponi* was thought to be the most primitive balaenopterid by Bisconti & Bosselaers (2016); and *‘Balaenoptera’ cortesii* var. *portisi* was supposed to be the most primitive balaenopterid by Deméré, Berta & McGowen (2005). These taxa are arranged at the earliest nodes in the balaenopterid phylogeny in our hypothesis. More advanced balaenopterid clades are then individuated: from node 16 to 27 including a variety of living and extinct advanced balaenopterid taxa such as *‘Balaenoptera’ siberi*, *Diunatans luctoretemergo*, *‘Megaptera’ miocaena*, Maesawa-cho (that is worth a formal description with taxonomic identification), *M. novaeangliae* and the living *Balaenoptera* species. This result is in partial agreement with the hypothesis provided by Peredo et al. (2018) in which the modern *Balaenoptera* is monophyletic to the exclusion of *Megaptera*.

Such a result is in striking contrast to molecule- and total evidence-based results that support a view in which *Megaptera* is nested within *Balaenoptera* thus making *Balaenoptera* itself a paraphyletic group (Slater, Goldbogen & Pyenson, 2017; Marx & Kohno, 2016; Gatesy et al., 2013). This hypothesis is not supported by our observations of the balaenopterid morphology and by our phylogenetic results.

The clade branching from node 18 of Fig. 20 includes a number of balaenopterid taxa with advanced morphological characters that have been included, in the past, in the same genera as the living balaenopterids (i.e., *Balaenoptera* and *Megaptera*). Subsequent analyses made it clear that different generic names are necessary to correctly identify some of them (e.g., *‘Balaenoptera’ siberi*; see Bisconti, 2011; Bisconti & Bosselaers, 2016). The taxonomy and phylogenetic relationships of *‘Balaenoptera’ bertae* are a more complicated question. Boessenecker (2013) considered it as a member of the genus *Balaenoptera* and Peredo et al. (2018) included it within a living group of small-sized balaenopterid species including also *Balaenoptera borealis*, *Balaenoptera acutorostrata* and *Balaenoptera bonaerensis*. However, in consideration that *‘Balaenoptera’ bertae* exhibits (a) strongly protruded posterolateral corner of the exoccipital, (b) incavated supraorbital process of the frontal in its posteromedial border, (c) strongly reduced length of the zygomatic process of the squamosal that (d) reaches a point located highly ventral with respect to the postorbital corner of the supraorbital process of the frontal, and based on our phylogenetic results, we suggest that this taxon does not belong to the genus *Balaenoptera*.

### Evolution of balaenopterid diversity through time

Our study of the diversity radiations of Balaenopteridae through time revealed three distinct pulses of diversity increase intermixed with extinction events (Fig. 23). Comparing the observed pattern with the curve describing changes in temperature and δC_13_ provided by Zachos et al. (2001) it is clear that all the diversity peaks occurred during periods of temperature decline. In particular, the Event 1 occurred between *c.* 13 and 11 Ma when the East Antarctic ice-sheet accumulated; the Event 2 occurred between *c.* nine and seven Ma when global temperatures declined and the West Antarctic ice-sheet accumulated; the Event 3 occurred between four and two Ma when a drop in temperatures is recorded based on several sources (Zachos et al., 2001; Miller et al., 2005). Interestingly, the Event 1 was important also for other non-balaenopterid mysticetes but the other events are recorded only in Balaenopteridae and Balaenidae.

Marx & Uhen (2010) analyzed mysticete diversity trends in the past based on actual presence/absence of species in the fossil record. Their approach was different from ours because in our study we analyzed clade diversity including presence/absence of species and inferred presence/absence of lineages in time intervals. Our results are slightly different from those of Marx & Uhen (2010) in that our Event 1 is slightly younger than the main peak in mysticete diversity that they observed occurring between 12 and 14 Ma. The difference between our result and that of Marx & Uhen (2010) concerning this event is so slight that should be considered not significant. Interestingly, our results coincide with those of Marx & Uhen (2010) about the diversity peak occurred during the Pliocene that we assign to a major diversification event within Balaenopteridae and Balaenidae. Marx & Uhen (2010) observed a corresponding pattern in temperature change and diatom diversity in that in both the peaks they observed there was an increase in diatom diversity and a temperature decline. Our approach includes also inferred presence/absence of ghost lineages and suggests that the diversity change of mysticetes through time was influenced by more or more complex drivers but, as a general conclusion, it may be said that as a global cooling is connected to higher erosion rates, and as high erosion rates are connected to major pulses of nutrient availability in the oceans, then the major peaks in mysticete diversity observed here and by Marx & Uhen (2010) occurred when the available food resources were more abundant thus allowing survival of a higher number of clades.

The hypothesized link between Event 2 and a global cooling and enriched nutrient availability during the cooling of the mid-late Tortonian is also confirmed by palynological results in the Southern North Sea Basin (Donders et al., 2009). In several samples of the Westerschelde and Liessel areas increased numbers of *Habibacysta tectata,* a cold-adapted dinoflagellate cyst taxon are recorded. In addition, increased numbers of heterotrophic species in the present area indicate nutrient-rich marine conditions. A recent work also shows increased sedimentation rates in the area during the late Tortonian (Deckers & Louwye, 2019).

Zachos et al. (2001) record a dramatic drop in productivity based on δC_13_ starting from the Zanclean/Piacenzian transition. Productivity did not reach previous levels anymore in the next three million years. Based on our analysis and according to Marx & Uhen (2010) results about mysticetes, in the last three million years mysticete diversity declined. It is likely that the decrease of balaenomorph diversity observed since the mid-Pliocene is due to a reduction of food availability implying high competition rates among filter-feeding mysticetes that led to the survival of only a small amount of species. This hypothesis should be tested with appropriate methods that are outside the scope of the present paper.

### Conclusion: the mysticete fauna from the Neogene of the North Sea

The Miocene and Pliocene outcrops in the southern portion of the North Sea (occurring in Belgium, Denmark and Holland) are rich in fossil whales. The mysticete fauna from Belgium, mainly described by Van Beneden (Van Beneden, 1880; Van Beneden & Gervais, 1868) and Abel (1941), is formed by hundreds of fragments belonging to tens of individual whales that were variously assembled into discrete morphological groups following ambiguous rules. The resulting taxonomy does not appear as a well established one (Deméré, Berta & McGowen, 2005; Bisconti, Lambert & Bosselaers, 2013; Bosselaers & Post, 2010), therefore, the genera and species belonging into this fauna are in critical need of revision. However, as different approaches have been used to perform such a revision (Bosselaers & Post, 2010; Steeman, 2009; Bisconti, Lambert & Bosselaers, 2013, 2017), clear and unambiguous results are still lacking and further work appears necessary to make order into the Belgian fauna.

In the last decade, the mysticete fauna from Denmark revealed interesting data. Works by Steeman and coworkers (Steeman, 2007, 2009; Gol’din & Steeman, 2015) enlightened the morphology and relationships of important specimens including moderately well preserved skulls and postcrania and showing that basal thalassotherian taxa were present in the late Miocene of the North Sea together with a putative new family of Cetotheriidae-like mysticetes subsequently called Tranatocetidae. Given the nature of our dataset, we cannot confirm the monophyly of Tranatocetidae because most of the typical taxa of this family were not included into our matrix.

The mysticete fauna from The Netherlands is being unfolded just recently in a few papers (Marx, Bosselaers & Louwye, 2016; Marx et al., 2019; Bisconti, 2005, 2015) revealing well preserved cranial materials that may be really useful for taxonomy and phylogenetic inference.

**Figure 24:**
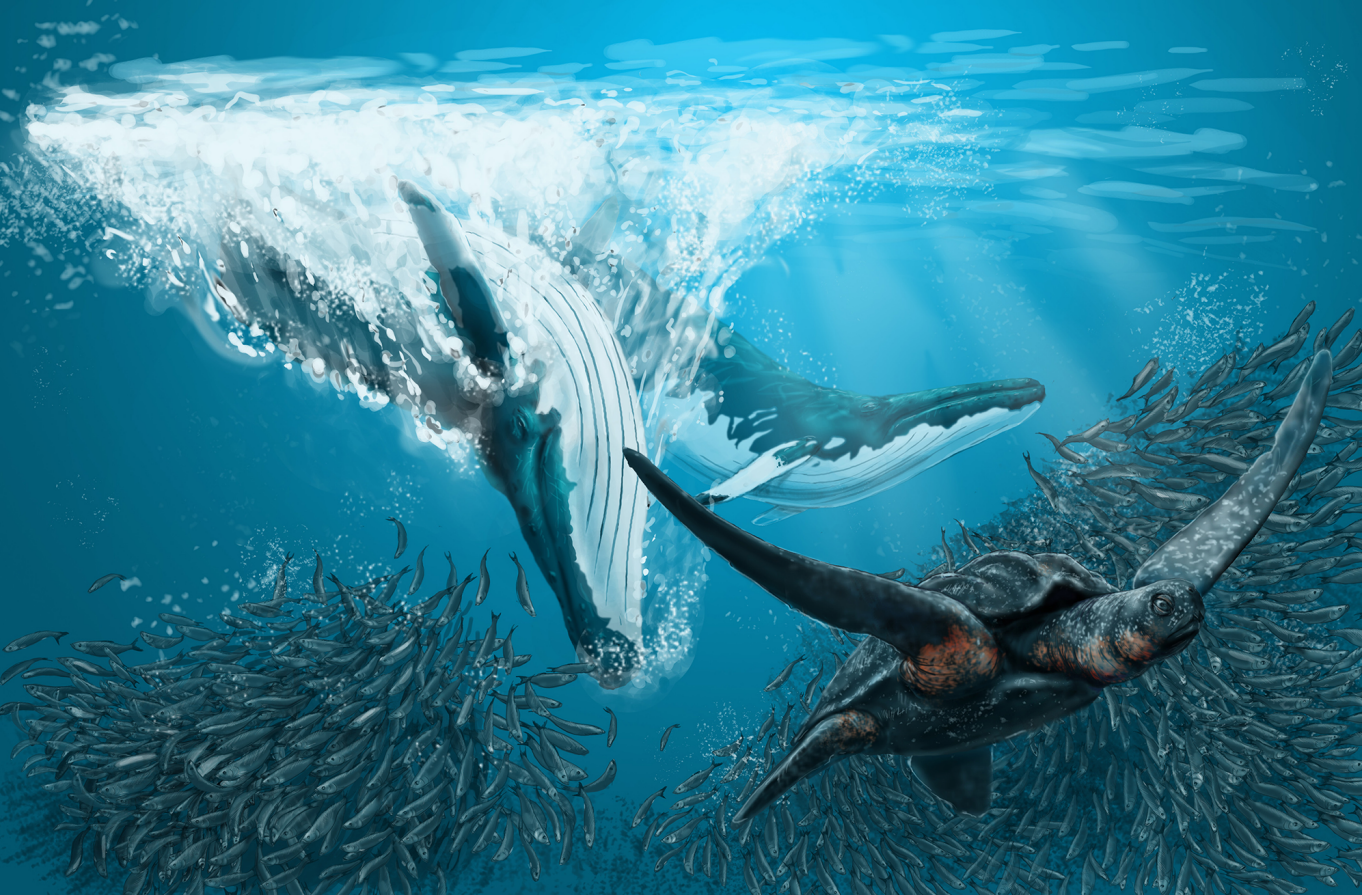
Artistic representation of *Nehalaennia devossi*. This artistic reconstruction of *Nehalaennia devossi* shows two individuals during feeding upon schooling fishes. The leatherback turtle is used as a reference to show the hypothesized size of the rorqual. Credits for the illustration: Remie Bakker, Manimal Works, Rotterdam, The Netherlands.

The recent discovery of a new fauna from the late Miocene Westerschelde adds new and significant information about the past diversity of northern Chaeomysticeti. This fauna is currently on exhibition (‘Zeeuwse Oerwalvissen – Topfossielen uit de Westerschelde’) at Het Natuurmuseum Rotterdam where it attracts visitors and press contributing to the diffusion of paleontological culture in The Netherlands (Fig. 24). In conclusion, we can state that the mysticete fauna from the North Sea will play a major role in deciphering the evolution of diversity of chaeomysticete whales from the Neogene (see also Marx et al., 2019 for a first result). New specimens are currently under preparation and other ones are under study by different authors and it is highly expected that these can shed further light on the evolution of baleen-bearing whales.

## Supplemental Information

10.7717/peerj.6915/supp-1 Supplemental Information 1 Phylogeny dataset.

10.7717/peerj.6915/supp-2 Supplemental Information 2 Supplemental Information. The Supplemental Information includes the (1) list of the specimens examined by the authors together with their repositories and accession numbers, (2) the character list realized by the authors, (3) the taxonx character matrix realized by the authors, (4) the phylogenetic results, (5) the clade numbers per time interval realized by the authors, (6) the geographic and stratigraphic occurrences of the taxa and the literature used as source, (7) the references cited in the Supplemental Information.

## Additional Information and Declarations

### Data Availability

The following information was supplied regarding data availability: The raw data is available as Supplemental Information, including: (1) stratigraphic ages and geographic ranges of the taxa included into the analyses, (2) descriptions of morphological characters used in phylogenetic analysis and (3) character x taxon matrix used in phylogenetic analysis. The list of the specimens examined by the authors includes both the repository (museum or other institutions where the specimens are located) and accession numbers together with the relevant literature used for gather our data. The sources of the geographic occurrences and stratigraphic ages of the taxa are available in the Supplemental Information File taxon by taxon. The character list and the taxon x character matrix were prepared by us with the help of the literature cited in the Supplemental Information File.

### New Species Registration

The following information was supplied regarding the registration of a newly described species: Publication LSID: urn:lsid:zoobank.org:pub:EB25A914-5D9E-4838-9C10-AAB9897E2B51. Nehalaennia LSID: urn:lsid:zoobank.org:act:5DCFFD28-B975-4A07-96EE-C136DE6B5771. *N. devossi* LSID: urn:lsid:zoobank.org:act:9D0B1C6D-1E43-4E16-8BB5-A1862633A8E9.
