# Supplementary material for: A new balaenopterid whale from the late Miocene of the Southern North Sea Basin and the evolution of balaenopterid diversity (Cetacea, Mysticeti)
Source: PeerJ. 2019 May 17;7:e6915. doi: 10.7717/peerj.6915 (PMC6526909; doi:10.7717/peerj.6915)
Supplement: Supplemental Information 2 — The Supplemental Information includes the (1) list of the specimens examined by the authors together with their repositories and accession numbers, (2) the character list realized by the authors, (3) the taxonx character matrix realized by the authors, (4) the phylogenetic results, (5) the clade numbers per time interval realized by the authors, (6) the geographic and stratigraphic occurrences of the taxa and the literature used as source, (7) the references cited in the Supplemental Information. [file peerj-07-6915-s002.pdf]

Supplementary Information to:

A new balaenopterid whale from the late Miocene of the Southern North Sea Basin and the evolution of balaenopterid diversity (Cetacea, Mysticeti)

Michelangelo Bisconti[1,2], Dirk K. Munsterman[3], Klaas Post[4]

[1]Paleobiology Department, San Diego Natural History Museum, 1788 El Prado, 90122 CA, United States of America
[2]Dipartimento di Scienze della Terra, Università di Torino, via Valperga Caluso 35, 10125, Torino
[3]Toegepast Natuurwetenschappelijk Onderzoek (TNO-Netherlands Organization for Applied Scientific Research), Geological Survey of The Netherlands, P.O. Box 80015, 3508 TA, Utrecht, The Netherlands
[4]Het Natuurhistorisch Museum, Westzeedijk 345, 3015 AA Rotterdam, The Netherlands

Corresponding author
Michelangelo Bisconti
Email address: michelangelobisconti@unito.it

Index

p. 2 Table S1. Stratigraphic and geographic data of the taxa used in the analyses
p. 4 Institutional abbreviations
p. 5 Specimens used for comparative analysis
p. 9 Outline of undescribed specimens
p. 10 Character List
p. 35 Taxon x character matrix used in phylogenetic analysis
p. 45 Table S2. Results of phylogenetic analysis
p. 46 Table S3. Dataset used for analysis of evolutionary radiation hypothesis
p. 47 Literature cited in the Supplementary Information

Table S1

Stratigraphic and geographic data for the taxa used in the analyses

Taxon name	FO (1)	LO (1)	Areas of occurrence	References
Protocetus atavus	48.6	40	Mediterranean	Paleobiology Database
Georgiacetus vogtlensis	40.4	37.2	North Atlantic	Paleobiology Database
Gaviacetus razai	48.6	40.4	Indian	Paleobiology Database
Maiacetus inuus	48.6	40.4	Indian	Paleobiology Database
Basilosaurus cetoides	37.2	33.9	Mediterranean<br>North Atlantic	Paleobiology Database
Cynthiacetus peruvianus	37.2	33.9	South Pacific	Paleobiology Database
Dorudon atrox	37.2	33.9	Mediterranean, North Atlantic	Paleobiology Database
Zygorhiza kochii	37.2	33.9	North Atlantic	Paleobiology Database
Mammalodon colliveri	28.4	23.03	South Pacific	Paleobiology Database
Janjucetus hunderi	23.9	27	South Pacific	Fitzgerald (2006)
Fucaia buelli	33.9	31	North Pacific	Marx et al. (2015)
Aetiocetus weltoni	28.4	23.3	North Pacific	Paleobiology Database
Waharoa ruwhenua	27.3	20.43	South Pacific	Boessenecker & Fordyce (2015)
Yamatocetus canaliculatus	28.4	23.3	North Pacific	Paleobiology Database
Eomysticetus whitmorei	28.4	23.3	North Atlantic	Paleobiology Database
Micromysticetus rothauseni	33.9	28.4	North Atlantic	Paleobiology Database
Horopeta umarere	27.3	25.2	South Pacific	Tsai & Fordyce (2015)
Sitsqwayk cornishorum	28.4	23.03	North Pacific	Peredo & Uhen (2016)
Morenocetus parvus	20.03	15.97	South Atlantic	Buono et al. (2018)
Miocaperea pulchra	11.608	7.246	South Pacific	Bisconti (2012)
Caperea marginata	0.012	0.0	South Pacific	Paleobiology Database
Balaenella brachyrhynus	5.3	5.0	North Sea	Bisconti (2005)
Balaena mysticetus	0.012	0.0	North Atlantic, North Pacific	Paleobiology Database
Eubalaena glacialis	0.012	0.0	North Atlantic	Paleobiology Database
Balaenula astensis	3.4	3.2	Mediterranean	Bisconti (2000)
Titanocetus sammarinensis	15.97	13.81	Mediterranean	Bisconti (2006)
Tiucetus rosae	13.65	7.246	South Pacific	Paleobiology Database
Metopocetus hunteri	11.608	7.246	North Sea	Paleobiology Database
Cophocetus oregonensis	20.43	15.97	North Pacific	Paleobiology Database
Aglaocetus moreni	20.43	15.97	South Atlantic	Paleobiology Database
Mixocetus elysius	11.608	7.246	North Pacific	Paleobiology Database
Uranocetus gramensis	11.608	7.246	North Sea	Paleobiology Database
Isanacetus laticephalus	20.43	15.97	North Pacific	Kimura and Ozawa (2002)
Metopocetus durinasus	15.97	13.65	North Atlantic	Paleobiology Database
Diorocetus hiatus	15.97	13.65	North Atlantic	Paleobiology Database
'Aglaocetus' patulus	15.97	13.65	North Atlantic	Paleobiology Database
Parietobalaena palmeri	15.97	13.65	North Atlantic	Paleobiology Database
Pelocetus calvertensis	15.97	13.65	North Atlantic	Paleobiology Database
Joumocetus shimizui	11.608	7.246	North Pacific	Kimura and Hasegawa (2010)
Parietobalaena campiniana	15.0	13.2	North Sea	Bisconti et al. (2013)
USNM 187416	17.0	15.0	North Atlantic	Gottfried et al. (1994)
Piscobalaena nana	11.608	4.0	South Pacific	Paleobiology Database
Herpetocetus morrowi	3.6	2.6	North Pacific	Paleobiology Database
Cetotherium riabinini	11.6	7.246	Paratethys	Paleobiology Database
Cetotherium rathkei	13.65	7.246	Mediterranean (Paratethys)	Paleobiology Database
Thinocetus arthritus	13.65	11.608	North Atlantic	Paleobiology Database
Halicetus ignotus	13.65	11.608	North Atlantic	Paleobiology Database
Herentalia nigra	11.608	7.246	North Sea	Paleobiology Database
Archaeschrichtius ruggieroi	11.0	7.5	Mediterranean	Bisconti & Varola (2006)
Eschrichtioides gastaldii	5.0	3.0	Mediterranean	Bisconti (2008)

Eschrichtius robustus	0.1	0.0	North Sea, North Atlantic, North Pacific	Paleobiology Database
'Balaenoptera' ryani	11.608	7.246	North Pacific	Paleobiology Database
RBINS M. 2231	5.0	5.0	North Sea	Bisconti & Bosselaers in prep. a
MPTAM 207.13307	3.6	3.2	Mediterranean	Bisconti et al. in prep.
Archaebalaenoptera castriarquati	3.8	2.558	Mediterranean	Bisconti (2007a); Freschi & Cau (2016)
Protororqualus cuvieri	3.1	3.0	Mediterranean	Bisconti (2007b); Freschi & Cau (2016)
'Balaenoptera' cortesii var. *portisi*	3.6	2.588	Mediterranean, North Atlantic, North Pacific	Deméré et al. (2005)
SAM PQL-55001	5.3	5.0	South Atlantic	Govender et al. (2016)
Plesiobalaenoptera quarantellii	11.608	7.246	Mediterranean	Bisconti (2010)
'Balaenoptera' bertae	3.35	2.5	North Pacific	Boessenecker (2013)
Parabalaenoptea baulinensis	7.246	5.332	North Pacific	Zeigler et al. (1997)
Fragilicetus velponi	5.332	5.0	North Sea	Bisconti and Bosselaers (2016)
'Megaptera' hubachi	5.332	3.6	South Pacific	Bisconti (2011)
Diunatans luctoretemergo	5.3	2.558	North Sea	Bosselaers & Post (2010)
'Balaenoptera' siberi	7.246	5.332	South Pacific	Paleobiology Database
MHNL 1610	8.0	7.0	South Pacific	Bisconti et al. (in prep. a)
MHNL 1613	7.5	7.3	South Pacific	Bisconti et al. (in prep. b)
UT PU13842/5	3.4	3.2	Mediterranean	Caretto (1970)
MAB002286	8.2	7.5	North Sea	Bisconti et al. (in prep. c)
RBINS 2315	3.71	2.74	North Sea	Bisconti & Bosselaers (in prep. b)
NMR 7096	3.7	2.7	North Sea	Bisconti & Bosselaers (in prep. c)
Incakujira anillodefuego	7.5	7.3	South Pacific	Marx & Kohno (2016)
Megaptera novaeangliae	0.781	0.0	North Atlantic, North Pacific, South Atlantic, South Pacific, Indian Ocean	Paleobiology Database;
'Balaenoptera' bertae	5.332	2.558	North Pacific	Boessenecker (2013)
Miobalaenoptera numataensis	6.8	6.5	North Pacific	Tanaka & Watanabe (2019)
'Megaptera' miocaena	11.608	7.246	North Pacific	Paleobiology Database
Maesawa-Cho	5.3	5.0	North Pacific	Oishi et al. (1985)
Shimajirikujira	9.0	8.0	North Pacific	Kimura et al. (2015)
Balaenoptera borealis	2.6	0.0	North Atlantic, North Pacific, South Atlantic, South Pacific, Indian Ocean	Paleobiology Database
Balaenoptera edeni	0.012	0.0	North Atlantic, North Pacific, South Atlantic, South Pacific, Indian Ocean	Paleobiology Database
Balaenoptera musculus	1.806	0.0	North Atlantic, North Pacific, South Atlantic, South Pacific, Indian Ocean	Paleobiology Database
Balaenoptera omurai	0.012	0.0	North Pacific	Paleobiology Database
Balaenoptera brydei	0.012	0.0	North Pacific	Wada et al. (2007)
Balaenoptera physalus	1.3	0.0	North Atlantic, North Pacific, South Atlantic, South Pacific, Indian Ocean, Mediterranean	Paleobiology Database
Balaenoptera acutorostrata	3.6	0.0	North Atlantic, North Pacific, Mediterranean, Indian Ocean	Paleobiology Database
Balaenoptera bonaerensis	0.012	0.0	South Atlantic, South Pacific	Paleobiology Database

FO, first occurrence; LO, last occurrence. Data in Ma.

Institutional abbreviations

AMNH, American Museum of Natural History, New York, USA;
ChM, The Charleston Museum, Charleston, USA;
RBINS, Royal Belgian Institute of Natural Sciences, Brussels, Belgium;
MAB, Oertijdmuseum Boxtel, Bosscheweg 80, 5283 WB Boxtel, The Netherlands;
MAUL, Museo dell'Ambiente, Università di Lecce, Lecce, Italy;
MGB, Museo Geopalaeontologico 'G. Capellini', Bologna, Italy;
MCA, Museo Geopalaeontologico 'G. Cortesi', Castell'Arquato, Italy;
MHNL, Museo de Historia Natural, Lima, Peru;
MPTAM, Ente Gestione Aree Protette Artigiane, Asti, Italy and Museo Paleontologico Territoriale dell'Astigiano e del Monferrato, Asti, Italy;
MRSN, Museo Regionale di Scienze Naturali, Torino, Italy;
MSNT, Museo di Storia Naturale del Territorio, Calci, Italy;
MPST, Museo Palaeontologico, Salsomaggiore Terme, Italy;
NBC, Naturalis Biodiversity Center, Leiden, Holland.
NHG, Natuurlijke Historie Genootschap, Zeeuws Museum, Middelburg, The Netherlands;
NMB, NatuurMuseum Brabant, Tilburg, Holland;
NMR, Natuurhistorisch Museum, Rotterdam, Holland;
RBINS, Royal Belgian Institute of Natural Sciences, Brussels, Belgium;
SMSN, Staatliches Museum für Naturkunde, Stuttgart, Germany;
USNM, United States National Museum of Natural History, Smithsonian Institution, Washington, DC, USA;
ZMA, Instituut voor Systematiek en Populatiebiologie/Zoölogisch Museum, Amsterdam, Holland (the zoological and paleontological collections of ZMA recently moved to NBC).

Specimens used in the comparative analysis

The specimens listed below were examined by one or all the authors. In some cases, it was not possible to directly examine the specimens; in those cases, the relevant literature is provided in the list below. The list includes 8 undescribed taxa that are included in a phylogenetic analysis for the first time in this paper. These taxa are from Italy (MPTAM 207.13307 and UT PU13842/5), Belgium (RBINS M. 2231, M. 2315, NMR 7096, MAB002286) and Peru (MHNL 1610 and 1613). All these new taxa are balaenopterids and their publications are in progress. Relative ages of the species listed below are from the Cetacea section of the Paleobiology Database mostly compiled by Mark Uhen and, for undescribed taxa, from the cited literature.

1. **Protocetidae**
   We compiled the matrix by using the following taxa:
   (i) *Protocetus atavus*: SMNS 11084 (holotype); middle Eocene.
   (ii) *Georgiacetus vogtlensis*: Hulbert *et al*. (1996), Hulbert (1998); middle Eocene.
   (iii) *Maiacetus inuus*: Gingerich et al. (2009); middle Eocene.
   (iv) *Gaviacetus razai*: Luo & Gingerich (1998); middle Eocene.
2. ***Basilosaurus cetoides***
   Kellogg (1936); Uhen (1998); late Eocene.
3. ***Cynthiacetus peruvianus***
   Martinez-Caceres & Muizon (2017); late Eocene-to-early Oligocene.
4. ***Dorudon atrox***
   Uhen (2004); late Eocene.
5. ***Zygorhiza kochii***
   USNM 4748, 16638, 449538; Kellogg (1936), Uhen (1998); late Eocene.
6. ***Aetiocetus weltoni***
   Barnes *et al.* (1994), Deméré & Berta (2008); late Oligocene.
7. **Mammalodontidae**
   We compiled the matrix by using the following taxa:
   (i) *Mammalodon colliveri*: Fitzgerald (2010); late Oligocene.
   (ii) *Janjucetus hunderi*: Fitzgerald (2006); late Oligocene.
8. ***Fucaia buelli***
   Marx et al. (2015); early Oligocene.
9. ***Waharowa ruwhenua***
   Boessenecker & Fordyce (2015); late Oligocene.
10. ***Yamatocetus canaliculatus***
    Okazaki (2012); late Oligocene.
11. ***Micromysticetus rothauseni***
    Sanders & Barnes (2002a); late Oligocene.
12. ***Eomysticetus whitmorei***
    ChM PV4253 (holotype), Sanders & Barnes (2002b); late Oligocene.
13. ***Horopeta umarere***
    Tsai & Fordyce (2015); late Oligocene.
14. ***Sitsqwayk cornishorum***
    Peredo & Uhen (2016); late Oligocene
15. ***Morenocetus parvus***
    Buono et al. (2018): early Miocene.
16. ***Caperea marginata***
    AMNH AMO 36692; RBINS 1536; Baker (1985), Beddard (1901); Recent.
17. ***Miocaperea pulchra***
    Bisconti (2012); late Miocene.

18. *Balaena mysticetus*
USNM 257513; ZML 1680, 3997, 2563, 2001; Bisconti (2003), Burns *et al.* (1993), Reeves & Leatherwood (1985); Recent.

19. *Balaenula astensis*
MSNT MC CF 35 (holotype); Bisconti (2000); early Pliocene.

20. *Balaenella brachyrhynus*
Natuurmuseum Brabaant (Tilburg), specimen 42001 (holotype); Bisconti (2005); early Pliocene.

21. *Eubalaena glacialis*
AMNH 42752, 256803, 90241; MSNT 264; USNM 267612, 3339990, 23077, 301637; Bisconti (2003), Cummings (1985a), True (1904); Recent.

22. *Tiucetus rosae*
Marx et al. (2017); middle-to-late Miocene.

23. *Pelocetus calvertensis*
USNM 11976 (holotype); Kellogg (1965); middle Miocene.

24. *'Aglaocetus' patulus*
USNM 13472; Kellogg (1968c); middle Miocene.

25. *Uranocetus gramensis*
Steeman (2009); middle-to-late Miocene.

26. *Isanacetus laticephalus*
Kimura & Ozawa (2002); early Miocene.

27. *Joumocetus shimizui*
Kimura & Hasegawa (2010); late Miocene.

28. *Parietobalaena palmeri*
AMNH 128885; USNM 10677, 16570, 24883, 10909; Kellogg (1968d); middle Miocene.

29. *Parietobalaena campiniana*
RBINS M.399-R.4018 (holotype); Bisconti *et al.* (2013); middle Miocene.

30. *Diorocetus hiatus*
USNM 16783 (holotype), 205990; Kellogg (1968b); middle Miocene.

31. USNM 187416; middle Miocene.

32. *Herpetocetus morrowi*
El Adli et al. (2014); late Pliocene.

33. *Piscobalaena nana*
Bouetel & De Muizon (2006); late Miocene-to-early Pliocene.

34. *Cetotherium rathkei*
Pilleri (1986); middle Miocene.

35. *Cetotherium riabinini*
Gol'Din et al. (2014); late Miocene.

36. *Mixocetus elysius*
Kellogg (1934b); late Miocene.

37. *Metopocetus hunteri*
Marx et al. (2015); late Miocene.

38. *Metopocetus durinasus*
USNM 60460 (holotype); Kellogg (1968a); late Miocene.

39. *Herentalia nigra*
Bisconti (2015); late Miocene.

40. *Cophocetus oregonensis*
Packard & Kellogg (1934); early Miocene.

41. Aglaocetus moreni
Kellogg (1934a); early Miocene.

42. Thinocetus arthritus
Kellogg (1969a); late Miocene.
43. Halicetus ignotus
Kellogg (1969b); late Miocene.
44. *Eschrichtius robustus*
AMNH 181374, 34260, 1750 (‘*Eschrichtius cephalum*’), A; NMB 42001; USNM 364969, 364580, 571931, 364969, 364977, 364970, 364973, 504305; ZML St20350, St13130, 630. Andrews (1914).
45. *Eschrichtioides gastaldii*
MGPT 13802 (holotype); Bisconti (2008); early Pliocene.
45. *Archaeschrichtius ruggieroi*
MAUL 230/1; Bisconti & Varola (2006); late Miocene.
46. *Titanocetus sammarinensis*
MGB 9073 1CMC172 (1-6) (holotype); Bisconti (2006); middle Miocene.
47. *‘Balaenoptera’ ryani*
Hannah & McLellan (1924); late Miocene.
48. *Archaebalaenoptera castriarquati*
holotype (inventory of the Soprintendenza per i Beni Archeologici dell’Emilia Romagna item No. 240536; MCA): Bisconti (2007a); late Pliocene.
49. *Protororqualus cuvieri*
Bisconti (2007b); late Pliocene.
50. *‘Balaenoptera’ cortesi* var. *portisi*
Sacco (1890); Portis (1884); early Pliocene.
51. *Plesiobalaenoptera quarantellii*
holotype (inventory of the Soprintendenza per i Beni Archeologici dell’Emilia Romagna item No. 240505; MPST); Bisconti (2010); late Miocene.
52. *Parabalaenoptera baulinensis*
Zeigler *et al*. (1997); late Miocene.
53. *Fragilicetus velponi*
Bisconti & Bosselaers (2016); early Pliocene.
54. UT PU13842/5
Caretto (1970); early Pliocene.
55. *Miobalaenoptera numataensis*
Tanaka & Watanabe (2019); late Miocene.
56. Shimajiri-kujira
Kimura et al. (2015); late Miocene.
57. Maesawa-cho
Oishi (1984); early Pliocene.
58. *‘Megaptera’ hubachi*
Dathe (1983); Bisconti (2011); middle Pliocene.
59. *Megaptera novaeangliae*
AMNH 24679; MSNT 263; USNM 269982, 486175 (1-2), 13656/16252, 21492; ZMA 14964, 14953 (1-2), 14952 (1-2), 14965, 14966, 14967; Winn & Reichley (1985); Recent.
60. *Diunatans luctoretemergo*
NHG 22279 holotype; Bosselaers & Post (2010); early Pliocene.
61. *‘Balaenoptera’ siberi*
Pilleri (1989); late Miocene.
62. *‘Balaenoptera’ bertae*
Boessenecker (2013); early-to-late Pliocene.

63. ***'Megaptera' miocaena***
Kellogg (1925); Late Miocene.
64. ***Balaenoptera omurai***
Wada et al. (2003); Yamada et al. (2008); Recent.
65. ***Balaenoptera acutorostrata***
AMNH 181411, 35680; RBINS 1537; MSNT 260, 261; ZMA 12873; Stewart & Leatherwood (1985), True (1904); Recent.
66. ***Balaenoptera bonaerensis***
Omura (1975); Recent.
67. ***Balaenoptera physalus***
AMNH 35026, 256796; MSNT 251, 252, 253, 258, 255, 257; ZMA 14950 (1-2), 14927 (1-2), 14935 (1-2), 23353, 14947; Gambell (1985*a*); Recent.
68. ***Balaenoptera musculus***
AMNH 234949, 256797, 256798; MSNT 250; ZMA 23356, 23354, 23355, 14946, 14942, 14961; Yochem & Leatherwood (1985), True (1904); Recent.
69. ***Balaenoptera edeni***
USNM 504692, 236680 (1-3); Cummings (1985b); Recent.
70. ***Balaenoptera brydei***
NBC Reg. 4003; NBC RGM 17712; Yamada et al. (2008); Recent.
71. ***Balaenoptera borealis***
USNM 504699, 504698, 504701, 504244, 486174; Gambell (1985b); Recent.
72. ***Nehalaennia devossi***
NMR 14035; this work; late Miocene.
73. MPTAM 207.13307
Bisconti et al. (in prep. a); early Pliocene.
74. NMR 7096
Bisconti & Bosselaers (in prep. a); late Pliocene.
75. RBINS M. 2231
Bisconti & Bosselaers (in prep. b); early Pliocene.
76. RBINS M. 2315
Bisconti & Bosselaers (in prep. c); early Pliocene.
77. MAB002286
Bisconti et al. (in prep b); Late Miocene.
78. SAM55001
Govender et al. (2017); late Miocene.
79. ***Incakujira anillodefuego***
Marx & Kohno (2016); late Miocene.
80. MHNL 1610
Bisconti et al. (in prep. c); late Miocene.
81. MHNL 1613
Bisconti et al. (in prep. d); early Pliocene.

Outline of undescribed specimens

MPTAM 207-13307
This specimen represents a new genus and species of Balaenopteridae whose description is now complete. It includes an incomplete skull with periotic still in articulation and part of the postcrania. The estimated age is earliest Piacenzian.

NMR 999100007096
This specimen includes skull, periotic and part of the postcrania. Its morphology suggests a close relationship to 'Balaenoptera' portisi. In the remainder of the paper and in the illustrations it is called NMR 7096. The estimated age is early Piacenzian.

RBINS M. 2231
This specimen includes skull, periotics, dentaries and part of the postcrania. It is closely related to *'Balaenoptera' sibbaldina* of which it represents the first reasonably complete skeleton. The specimen is briefly presented in Bisconti & Bosselaers (2014) and a full description is currently in progress. The specimen is currently held by RBINS. The estimated age is Early Pliocene.

RBINS M. 2315
The specimen includes partial skull and postcrania of a Pliocene individual very close to *Protororqualus cuvieri*. The description of this specimen is currently in progress.

MHNL 1613
The specimen includes a large skull with periotics still in articulation. It represents a new balaenopterid genus characterized by wide exposure of parietal at the cranial vertex. A description is currently close to be finished. The estimated age is Late Miocene.

MHNL 1610
The specimen includes a partially prepared skull with fragments of dentary. Its morphology suggests close relationships with *Archaebalaenoptera castriarquati* of which it could be an additional species. A description is currently in progress. The estimated age is Late Miocene.

Character list

The following character list is developed from the morphological dataset of Bisconti & Bosselaers (2016). In the present dataset, character states were redefined and re-formulated in order to warrant clear understanding. In defining character states, we made use of personal observations on specimens listed above and of literature. In particular, we need to cite the following papers that we used for character definitions and codings: Boessenecker & Fordyce (2015); Fordyce & Marx (2012); Steeman (2009); Geisler & Sanders (2003); Kimura & Ozawa (2001); Kellogg (1923); Miller (1925).

ROSTRUM: PREMAXILLA, MAXILLA, NASAL

1) Rostrum length:
- (0) Rostrum length shorter or equal to neurocranium length;
- (1) Rostrum length longer than neurocranium length.

2) Rostrum width:
- (0) Rostrum narrow;
- (1) Rostrum wide.

3) Rostrum straight:
- (0) Yes;
- (1) No, rostrum highly arched.

4) Rostrum arc:
- (0) Continuous;
- (1) Discontinuous.

5) Mesorostral groove:
- (0) Absent;
- (1) Present.

6) Ventral keel along rostrum:
- (0) Absent;
- (1) Present.

7) Premaxilla widens at anterior end:
- (0) No;
- (1) Yes.

8) Premaxillary foramen:
- (0) Present;
- (1) Absent.

9) Posterior end of premaxilla:
- (0) More anterior than frontonasal suture;
- (1) At posterior end of nasal;
- (2) Anterior to nasal.

10) Sutural contact between rostrum and frontal limited to ascending process of the maxilla:
- (0) No;
- (1) Yes.

11) Premaxilla and frontal articulation:
- (0) Sutured;
- (1) Not sutured.

12) External surface of maxilla:
- (0) Sub-vertical;
- (1) Sub-horizontal.

13) Medial border of maxilla anterior to narial fossa:
(0) Straight;
(1) Sinuous.
14) Lateral border of maxilla:
(0) Uniformly concave;
(1) Straight;
(2) Uniformly convex;
(3) Sinuous
15) Thickness of lateral border of maxilla:
(0) Thin;
(1) Thick.
16) Lateral process of maxilla:
(0) Absent;
(1) Present.
17) Length of lateral process of maxilla:
(0) Short;
(1) Long.
(2) Very long.
18) Position of external apex of lateral process of maxilla:
(0) Anterior to antorbital corner of orbit;
(1) Anterior and medial to orbit.
19) Infraorbital process of maxilla:
(0) Absent;
(1) Present.
20) Ascending process of maxilla:
(0) Absent;
(1) Present.
21) Width of ascending process of maxilla relative to its length:
(0) Narrow;
(1) Wide.
22) Length of ascending process of maxilla:
(0) Short;
(1) Long;
23) Lateral border of ascending process of maxilla:
(0) Forms an evident corner with posterior border of maxilla;
(1) Forms a wide curve with posterior border of maxilla.
24) Position of posterior ends of ascending processes of maxillae:
(0) Posterior ends do not meet along midline;
(1) Posterior ends meet along midline.
25) Meeting of ascending processes of the maxillae along the longitudinal axis of the skull:
(0) Contact limited to posterior corners;
(1) Contact extended to most of medial borders of the ascending processes of the maxillae.
26) Shape of posterior end of ascending process of maxilla at adulthood:
(0) Triangular;
(1) Squared;
(2) Rounded.
27) Shape of posterior end of ascending process of maxilla during late ontogeny:
(0) Triangular;
(1) Squared;
(2) Rounded.

28) Lateral and medial borders of ascending process of maxilla:
(0) Anteriorly diverging;
(1) Parallel;
(2) Anteriorly converging.
29) Position of posterior end of maxilla:
(0) Anterior to nasal;
(1) At level of posterior end of nasal;
(2) Posterior to nasal.
30) Position of posterior ends of maxillae:
(0) Transversely far;
(1) Transversely close;
(2) Transversely very close.
31) Numerous dorsal infraorbital foramina:
(0) Absent (only one foramen is present);
(1) Present.
32) Location of dorsal infraorbital foramina:
(0) Scattered along dorsal surface of maxilla;
(1) Mostly located close to the medial border of maxilla.
33) Medial border of maxilla:
(0) not relieved;
(1) relieved and forming a crest.
34) Antorbital notch:
(0) Absent;
(1) Present.
35) Shape of antorbital notch:
(0) Concavity in anterior edge of lateral process of maxilla without medial-projecting groove;
(1) Developed along medial-projecting groove.
36) Articulation between maxilla and frontal:
(0) Tight;
(1) Loose.
37) Maxillary pocket:
(0) Absent;
(1) Present.
38) Infraorbital plate visible in dorsal view:
(0) No;
(1) Yes.
39) Teeth at adulthood in maxilla and premaxilla:
(0) Present;
(1) Absent.
40) Grooves for vasculature of baleen epithelium:
(0) Absent;
(1) Present.
41) Fissure located along posterior border of maxilla in ventral view:
(0) Absent;
(1) Present.
42) Elongation of fissure:
(0) Fissure short;
(1) Fissure long.

43) Nasal length:
(0) Nasal reaching the anterior 20% of rostrum;
(1) Nasal reaching approximately rostrum midlength;
(2) Nasal reaching the posterior 20% of rostrum;
(3) Nasal reaching a point close to the anterior border of the supraorbital process of frontal.
(4) Nasal reaching a point located within the interorbital region of the frontal.

44) Anterior border of nasal:
(0) Concave;
(1) Straight;
(2) Convex.

45) Median keel in nasal:
(0) Absent;
(1) Present.

46) Position of anterolateral corner of nasal:
(0) Anterior to anteromedial corner;
(1) Lateral to anteromedial corner;
(2) Posterior to anteromedial corner.

47) Position of frontonasal suture:
(0) At anterior border of interorbital region of frontal;
(1) Well within interorbital region of frontal.

48) Nasal borders:
(0) With a concavity at midlength
(1) Converging anteriorly;
(2) Parallel-to-subparallel;
(3) Diverging anteriorly.

49) Nasal width:
(0) Nasal transversely wide;
(1) Nasal with strong transverse compression along its entire length.

FRONTAL

50) Shape of supraorbital process of frontal:
(0) Flat and forming a dorsal shield;
(1) descending from interorbital region of frontal;

51) Diversity of depressions:
(0) No depression;
(1) Gentle depression from interorbital region of frontal;
(2) Abrupt depression from interorbital region of frontal;

52) Cross-sections of depressions:
(0) No depression;
(1) Triangular;
(2) Laterally concave;
(3) Squared;
(4) Half-circle.

53) Anteroposterior length of supraorbital process of frontal;
(0) Short;
(1) Long;
(2) Very long.

54) Transverse diameter of supraorbital process of frontal:
(0) Short;
(1) Long;
(2) Very long.

55) Anterior border of supraorbital process of frontal:
(0) Directed posteriorly;
(1) Directed transversely;
(2) Directed anteriorly.

56) Anterior border of supraorbital process of frontal:
(0) Straight;
(1) Convex;
(2) Concave.

57) Backing of central and distal portions of the anterior border of the supraorbital process of frontal from its anteromedial corner:
(0) Absent;
(1) Present.

58) Posterior border of supraorbital process of frontal:
(0) Uniformly concave;
(1) Medial concavity;
(2) Straight.

59) Posterior border of supraorbital process of frontal:
(0) Directed posteriorly;
(1) Directed transversely;
(2) Directed anteriorly.

60) Supraorbital foramina:
(0) Present;
(1) Absent.

61) Orbitotemporal crest:
(0) Along posterodorsal edge of supraorbital process of frontal;
(1) From postorbital corner to anteromedial end of supraorbital process of frontal;
(2) Forming a curve from postorbital corner onto dorsal surface of supraorbital process of frontal;
(3) Forming a curve along anterior edge of supraorbital process of frontal.

62) Orbitotemporal crest:
(0) Well developed and sharp;
(1) Well developed and rounded;
(2) Highly reduced to a line.

63) Superimposition of parietal on interorbital region of frontal:
(0) Absent;
(1) Present.

64) Long superimposition of posteromedial elements of rostrum on interorbital region of frontal:
(0) Absent;
(1) Present.

65) Posterior border of interorbital region of frontal:
(0) In contact with parietal;
(1) In contact with supraoccipital.

66) Shape of coronal (frontal-parietal) suture:
(0) Straight;
(1) Anteriorly convex;
(2) Anteriorly concave.

67) Coronal suture in dorsal view:
(0) Visible;
(1) Not visible because superimposed by the supraoccipital.

68) Frontal encircles ascending process of maxilla:
(0) No;
(1) Yes.

69) Postorbital process and zygomatic process of squamosal:
(0) Far;
(1) Close;
(2) Superimposed and articulated by dedicate facet.

70) Location of optic canal in ventral surface of supraorbital process of frontal:
(0) Along anterior three-fourth;
(1) Along posterior one-fourth.

71) Length of intertemporal constriction:
(0) Very long;
(1) Long;
(2) Short;
(3) Very short.

72) Transverse diameter of intertemporal constriction:
(0) Wide;
(1) Moderately constricted;
(2) Highly constricted.

73) Presence of narial process:
(0) Present;
(1) Absent.

74) Length of narial process relative to nasal length:
(0) Short;
(1) Long.

75) Shape of narial process:
(0) The narial processes form a triangle in dorsal view;
(1) The narial processes form a bilobated protrusion in dorsal view.

PARIETAL

76) Location of frontal border of parietal:
(0) Posterior to posterior apex of ascending process of maxilla;
(1) Anterior to posterior apex of ascending process of maxilla.

77) Anterolateral corner of parietal (for Balaenidae only):
(0) Sharp;
(1) Broad.

78) Anterior portion of external surface:
(0) Visible in dorsal view;
(1) Not visible in dorsal view because overhanged by temporal crest.

79) Posterior portion of external surface:
(0) Visible in dorsal view;
(1) Not visible in dorsal view because overhanged by temporal crest.

80) Post-parietal foramen:
(0) Present;
(1) Absent.

81) Parietal spreading onto emergence of supraorbital process of frontal:
(0) Absent;
(1) Present.

82) Parietal exposed at cranial vertex:
(0) Yes;
(1) No.

83) Length of parietal exposure at vertex:
(0) Long;
(1) Moderate;
(2) Short;
(3) Very short.
84) Sagittal crest at cranial vertex:
(0) Present;
(1) Absent.
85) Attach for temporalis muscle at intertemporal constriction:
(0)Very narrow;
(1)Slightly widened;
(2)Moderately widened;
(3)Wide.
86) Shape of sagittal crest:
(0) Sharply-edged;
(1) Forming two opposite concavities.
87) Tubercle at lambdoid suture:
(0) Absent;
(1) Present.
88) Parietal-squamosal suture:
(0) Sinuous;
(1) Straight.

SQUAMOSAL
89) Dorsoventral height of squamosal:
(0) Low dorsoventral height;
(1) High dorsoventral height.
90) Anteroposterior length of zygomatic process of squamosal:
(0) Very long;
(1) Long;
(2) Short.
(3) Very short.
91) Height of zygomatic process of squamosal:
(0) Zygomatic process higher than postglenoid process;
(1) Zygomatic process at the same level of postglenoid process;
(2) Zygomatic process much higher than postglenoid process.
92) Projection of anterior portion of zygomatic process of squamosal in dorsal view:
(0) Projecting anteromedially;
(1) Projecting anterolaterally;
(2) Projecting anteriorly.
93) Projection of posterior portion of zygomatic process of squamosal in dorsal view:
(0)Projecting anteromedially;
(1)Projecting anterolaterally
(2)Projecting anteriorly.
94) Zygomatic process of squamosal in dorsal view:
(0) Anteriorly straight;
(1) Anteriorly twisted.
95) Distinctive articular facet for postorbital process of frontal on zygomatic process of squamosal:
(0) Absent;
(1) Present.

96) Projection of apex of zygomatic process in lateral view:
(0)Anterior;
(1)Ventral.
97) Postglenoid process of squamosal:
(0) Projecting ventrally;
(1) Projecting posteroventrally.
98) Twisted postglenoid process of squamosal:
(0) No;
(1) Yes.
99) Lateral surface of squamosal:
(0) Smooth;
(1) With single fossa for sternomastoid muscle;
(2) With double fossa for sternomastoid muscle.
100) Anteroposterior concavity along dorsolateral edge of glenoid fossa of squamosal:
(0) Absent;
(1) Present.
101) Glenoid fossa of squamosal:
(0) Forming a right angle in lateral view;
(1) Slightly concave;
(2) Highly concave (half-moon shaped);
(3) Straight.
102) Location of glenoid fossa of squamosal:
0) posterior to orbit;
1) immediately posteroventral to orbit.
103) Height of squamosal at nuchal crest:
(0) Low;
(1) High.
104) Supramastoid crest:
(0) Present;
(1) Absent.
105) Orientation of supramastoid crest:
(0) Dorsal;
(1) Anterior.
106) Nuchal crest in dorsal view:
(0) Wide;
(1) Narrow;
(2) Very narrow.
107) Nuchal crest in dorsal view:
(0) Circular;
(1) Triangular.
108) Nuchal crest in dorsal view:
(0) Reaching a point anterior to occipital condyle;
(1) Reaching a point posterior to occipital condyle;
(2) Reaching a point at the same level as occipital condyle.
109) Squamosal bulging into temporal fossa:
(0) No;
(1) Yes.
110) Extension of temporal fossa:
(0) Very wide;
(1) Wide;
(2) Reduced.

111) Extension of temporal fossa:
(0) Longer than wide;
(1) Wider than long.
112) Shape of temporal fossa in dorsal view:
(0) Oval;
(1) Almond-shaped;
(2) Triangular.
113) Surface of temporal fossa anterior to nuchal crest:
(0) More horizontal than ventral-most portion;
(1) Developed dorsoventrally.
114) Squamosal cleft:
(0) Absent;
(1) Present.
115) Shape of squamosal cleft:
(0) Straight;
(1) Triangular.
116) Length of squamosal cleft:
(0) Short;
(1) Long;
(2) Very long.
117) Origin of squamosal cleft at adulthood:
(0) From parietal-squamosal suture;
(1) From parietal-squamosal-alisphenoid suture;
(2) From squamosal-alisphenoid suture;
(3) From squamosal-pterygoid suture.
118) Origin of squamosal cleft during late ontogeny:
(0) From parietal-squamosal suture;
(1) From parietal-squamosal-alisphenoid suture;
(2) From squamosal-alisphenoid suture;
(3) From squamosal-pterygoid suture.
119) Infundibulum of Foramen ovale:
(0) Absent;
(1) Present.
120) Foramen ovale:
(0) Infundibulum complete;
(1) Infundibulum incomplete.
121) Foramen ovale:
(0) Located within squamosal;
(1) Located between squamosal and pterygoid.
(2) Located within pterygoid.
122) Suture present in foramen ovale:
(0) No;
(1) Yes.
123) Squamosal crease:
(0) Absent;
(1) Present.
124) Secondary squamosal crest:
(0) Absent;
(1) Present.

125) Secondary squamosal fossa:
- (0) Absent;
- (1) Present.

126) Basicranial foramina:
- (0) Separate foramina in posterolateral portion of skull;
- (1) Foramina confluent into a single and large posterior lacerate foramen.

SUPRAOCCIPITAL

127) Supraoccipital in dorsal view:
- (0) Not visible because main development is dorsoventral;
- (1) Visible because it superimposes on parietal.

128) Anteroposterior supraoccipital elongation:
- (0) No anteroposterior elongation;
- (1) Short: supraoccipital superimposed on posterior portion of parietal;
- (2) Long: supraoccipital superimposed on most of parietal;
- (3) Very long: supraoccipital superimposed on whole parietal and part of interorbital region of frontal.

129) Anteroposterior supraoccipital elongation with respect to zygomatic process of squamosal:
- (0) Anterior border of supraoccipital reaching a point located more posteriorly than the anterior apex of the zygomatic process of squamosal;
- (1) Anterior border of supraoccipital reaching a point located more anteriorly than the anterior apex of the zygomatic process of squamosal.

130) Shape of anterior border of supraoccipital:
- (0) Round;
- (1) Triangular;
- (2) Squared;
- (3) Ogival.

131) Distinctive articular facets for ascending process of the maxilla in anterior border of supraoccipital:
- (0) Absent;
- (1) Present.

132) Size of anterior border of supraoccipital:
- (0) Wide;
- (1) Pointed;
- (2) Narrow.

133) Elevation of anterior border of supraoccipital in lateral view:
- (0) High elevation formed by dorsal protrusion of parietals lateral and in front of the anterior border of supraoccipital;
- (1) Low elevation without contribution by the parietal;
- (2) No elevation at all.

134) Distinctive depression in front to supraoccipital in lateral view:
- (0) Present;
- (1) Absent.

135) Dorsal surface of supraoccipital:
- (0) Concave;
- (1) Flat-to convex.

136) Attach sites for neck muscle attachments:
- (0) Not evident;
- (1) Well developed.

137) Attach sites for neck muscle attachments:
(0) Shaped as triangular relieves with flat surface;
(1) Shaped as tubercles.
138) External occipital crest:
(0) Absent;
(1) Present.
139) Lateral borders of supraoccipital in dorsal view:
(0) Not visible;
(1) Uniformly convex;
(2) uniformly straight;
(3) uniformly concave;
(4) sinuous because of the presence of a transverse constriction.
140) Position of transverse constriction of supraoccipital:
(0) In anterior-most portion;
(1) At mid-length;
(2) In posterior half.
141) Degree of transverse constriction:
(0) Scarce;
(1) Moderate;
(2) Strong.
142) Lateral borders of supraoccipital anterior to the transverse constriction:
(0) Concave;
(1) Straight-to-convex.
143) Length of external occipital protuberance:
(0) Long;
(1) Moderate;
(2) Short.
144) Anterolateral corner of supraoccipital:
(0) Not distinguishable;
(1) Collapsed into a single anterior point;
(2) Rounded;
(3) Squared.
145) Supraoccipital bent at midlength:
(0) No;
(1) Yes.

INTERPARIETAL
146) Interparietal:
(0) Absent;
(1) Present.
147) Shape of interparietal:
(0) Short;
(1) Long.
148) Shape of interparietal:
(0)Wide;
(1)Narrow.

JUGAL
149) Jugal elongation:
(0) Jugal elongated and mostly straight;
(1) Jugal short and rounded.

LACRIMAL

150) Lacrimal exposed in dorsal view:
(0) No;
(1) Yes.

151) Sutured lacrimal:
(0)Yes;
(1)No.

EXOCCIPITAL

152) Exoccipital in posterior view:
(0) Anterolateral border forming a right angle with lateral edge of supraoccipital;
(1) Anterolateral border continuous with lateral edge of supraoccipital.

153) Exoccipital development in posterior view:
(0) Exoccipital transversely elongated;
(1) Transverse elongation of exoccipital reduced.

154) Protrusion of posterolateral corner of exoccipital:
(0) At level of postglenoid process;
(1) Medial to postglenoid process.

155) Protrusion of posterolateral corner of exoccipital:
(0) Reaching a point more anterior than occipital condyles;
(1) Reaching a point more posterior than occipital condyles.

156) Protrusion of posterolateral corner of exoccipital:
(0) More posterior than postglenoid process of squamosal.
(1) More anterior than postglenoid process of squamosal;

157) Occipital condyle:
(0) Convex articular face;
(1) Flat-to-slightly convex articular face.

158) Neck of occipital condyle:
(0) Well developed;
(1) Indistinct.

159) Condyloid foramen:
(0) Present;
(1) Absent.

160) Foramen in jugular notch:
(0) Present;
(1) Absent.

BASIOCCIPITAL

161) Basioccipital crest:
(0) Absent;
(1) Present.

162) Fusion of medial crest of basioccipital crest and falcate process of basioccipital:
(0) Absent;
(1) Present.

ALISPHENOID

163) Alisphenoid exposure in temporal fossa:
(0) Present;
(1) Absent.

164) Size of alisphenoid exposure in temporal fossa:
(0) Large;
(1) Small;
(2) Very small.
165) Alisphenoid borders:
(0) Between frontal, parietal, squamosal and pterygoid;
(1) Between parietal, squamosal and pterygoid;
(2) Between parietal and squamosal;
(3) Between parietal and pterygoid.

PALATINE
166) Palatine reaching a point located close to posterior border of skull:
(0) No;
(1) Yes.

PTERYGOID
167) Pterygoid fossa:
(0) Absent;
(1) Present.
168) Pterygoid hamulus:
(0) Short;
(1) Well developed.
169) Ventral lamina of pterygoid:
(0) Absent;
(1) Present.
170) Pterygoid exposure in temporal fossa:
(0) Absent;
(1) Present.

PERIOTIC
171) Posterior process exposure in lateral wall of skull:
(0) Absent;
(1) Present.
172) Posterior process length:
(0) Short;
(1) Moderate;
(2) Long.
173) Posterior process size and shape:
(0) Prismatic and robust;
(1) Transversely compressed and flattened.
174) Facial sulcus along posterior process:
(0) Absent;
(1) Present.
175) Facial sulcus along posterior process:
(0) Short;
(1) Long.
176) Position of facial sulcus on posterior process:
(0) Along medial border and hidden in ventral view;
(1) Ventromedial;
(2) Completely ventral.

177) Borders of facial sulcus:
(0) Sulcus bordered by crests;
(1) Sulcus widened and bordered by narrow relieves.
178) Facial sulus completely included in a tube-like structure:
(0) No;
(1) Yes.
179) Shape of posterior border of posterior process:
(0) Clavate;
(1) Squared;
(2) Pointed.
180) Stylomastoid fossa:
(0) Not distinguishable;
(1) Elongated and shallow;
(2) Elongated and covered by a relieved dorsal edge in the posterior process;
(3) Short and included within posterior process as a notch.
181) Anterior process:
(0) Absent;
(1) Present.
182) Anterior process length:
(0) Short;
(1) Long.
183) Anterior process thickness:
(0) Thick;
(1) Thin;
(2) Blade-like.
184) Origin of anterior process:
(0) Abruptly depressed from dorsal surface of periotic;
(1) Anterior process continuous with dorsal surface of periotic.
185) Anterior process in dorsal (or ventral) view:
(0) Squared;
(1) Irregular shape;
(2) Triangular;
(3) Elliptical.
186) If triangular, medial edge of anterior process:
(0) Convex or straight;
(1) Concave.
187) If triangular, lateral edge of anterior process:
(0) Convex or straight;
(1) Concave.
188) If triangular, apex of anterior process:
(0) Round;
(1) Pointed.
189) Lateral tuberosity:
(0) Absent;
(1) Present.
190) Size of lateral tuberosity:
(0) Small;
(1) Large.
191) Shape of lateral tuberosity:
(0) Protruding and squared or rounded;
(1) Protruding and triangular.

192) Lateral process of anterior process:
(0) Absent;
(1) Present.
193) Length of lateral process of anterior process:
(0) Long;
(1) Short.
194) Shape of lateral process of anterior process:
(0) Broadly triangular;
(1) Sharply triangular.
195) Medial emergence of anterior process:
(0) Absent;
(1) Present.
196) Tensor tympani groove:
(0) Present;
(1) Absent.
197) Dorsal surface of periotic:
(0) Highly relieved;
(1) Low.
198) Highly relieved dorsal surface of periotic:
(0) Squared;
(1) Dome-shaped.
199) Dorsal surface of periotic and anterior process forming a straight line in medial view:
(0) No;
(1) Yes.
200) Suprameatal area:
(0) Concave;
(1) Gently descending;
(2) Convex and protruding.
201) Superior process:
(0) Present;
(1) Absent.
202) Size of superior process:
(0) Convex dorsal profile in medial view;
(1) Reduced to a low ridge;
(2) Absent.
203) During late ontogeny, internal acoustic meatus including:
(0) Tractus spiralis foraminosus, foramen singulare and endocranial opening of facial canal;
(1) Tractus spiralis foraminosus and foramen singulare.
204) At adulthood, internal acoustic meatus including:
(0) Tractus spiralis foraminosus, foramen singulare and endocranial opening of facial canal;
(1) Tractus spiralis foraminosus and foramen singulare.
205) Crista transversa during ontogeny:
(0) Septum-like;
(1) Thick.
206) Crista transversa during adulthood:
(0) Septum-like;
(1) Thick.
207) Position of crista transversa at adulthood:
(0) Does not reach medial rim of internal acoustic meatus;
(1) Reaches medial rim of internal acoustic meatus.

208) Fissure in endocranial opening of facial canal during ontogeny:
(0) Absent;
(1) Present.
209) Fissure in endocranial opening of facial canal at adulthood:
(0) Absent;
(1) Present.
210) Vascular groove:
(0) Evident;
(1) Reduced;
(2) Absent.
211) Transverse elongation of pars cochlearis:
(0) Short;
(1) Elongated.
212) Anteroposterior elongation of pars cochlearis:
(0) Short;
(1) Elongated.
213) Inflation of pars cochlearis:
(0) Absent;
(1) Present.
214) Anterior crest along pars cochlearis:
(0) Absent;
(1) Present.
215) Cochlear window (round window) and aperture for cochlear aqueduct (endolymphatic foramen) confluent during late ontogeny:
(0) No;
(1) Yes.
216) Cochlear window (round window) and aperture for cochlear aqueduct (endolymphatic foramen) confluent at adulthood:
(0) No;
(1) Yes.
217) Cochlear window (round window) and aperture for cochlar aqueduct (endolymphatic foramen) opening in a tube-like channel:
(0) No;
(1) Yes.
218) Promontorial groove:
(0) Absent;
(1) Present.
219) Size of promontorial groove:
(0) Small;
(1) Large.
220) Endocranial opening of facial canal connected to internal acoustic meatus by a groove:
(0) No;
(1) Yes.
221) Pyramidal process:
(0) Present;
(1) Absent.

TYMPANIC BULLA
222) Shape of posterior border:
(0) Bilobated;
(1) Transversely straight;

(2) Convex;
(3) Keeled.

223) Elongation of portion posterior to conical process:
(0) Present;
(1) Absent.

224) Posterior border fissurated:
(0) Yes;
(1) No.

225) Elliptical foramen:
(0) present;
(1) absent.

226) Ventral keel:
(0) Absent;
(1) Present.

227) Ventral concavity:
(0) Present;
(1) Absent.

228) involucral protrusion in dorsal view:
(0) Absent;
(1) Present.

229) Dorsal border of involucrum in medial view:
(0) Gently descending;
(1) Not descending.

230) Position of Eustachian opening relative to overall height of tympanic bulla:
(0) Low;
(1) High.

231) Eustachian opening bordered anteriorly:
(0) no;
(1) yes.

232) Flat posterior dorsomedial face:
(0) No;
(1) Yes.

233) Anterolateral expansion:
(0) Absent;
(1) Present.

234) Extension of anterolateral expansion:
(0) Short;
(1) Long.

235) Shape of anterolateral expansion in dorsal view:
(0)Round;
(1)Pointed.

236) Tympanic cavity:
(0) High;
(1) Low.

237) Height of tympanic bulla:
(0) High;
(1) Low.

238) Anterior border:
(0) Anteriorly convex;
(1) Anteriorly straight-to-concave.

239) Sigmoid process:
(0) Anteroposteriorly elongated;
(1) Transversely elongated.
240) Conical process:
(0) High;
(1) Very reduced.
241) Proportional size of tympanoperiotic complex with respect of head size:
(0) Large;
(1) Small.
242) Outer lip and dorsal border of involucrum:
(0) Descending parallel toward anterior end;
(1) Posteriorly diverging as the outer lip is more inclined than involucrum.

DENTARY
243) Cranio-mandibular joint:
(0) Tight;
(1) Loose.
244) Teeth on dentary at adulthood:
(0) Present;
(1) Absent.
245) Mental symphysis:
(0) Present;
(1) Absent.
246) Groove for mental ligament:
(0) Absent;
(1) Present.
247) Anterior torsion:
(0) Absent;
(1) Present.
248) Massive elongation of dentary ramus:
(0) Absent;
(1) Present.
249) Coronoid process height:
(0) High;
(1) Moderately high;
(2) Low;
(3) Very low-to-absent.
250) Postcoronoid crest:
(0) Absent;
(1) Present.
251) Postcoronoid fossa:
(0) Absent;
(1) Present.
252) Size of postcoronoid fossa:
(0) Wide;
(1) Small.
253) Satellite process:
(0) Absent;
(1) Present.

254) Size of satellite process:
(0) Large;
(1) Small.
255) Orientation of articular surface of mandibular condyle:
(0) Posterodorsal;
(1) Dorsal;
(2) Posterior.
256) Posterodorsal corner of dentary:
(0) Round;
(1) Sharp.
257) Angular process:
(0) High;
(1) Moderately high;
(2) low;
(3) Very low.
258) Angular process in lateral view:
(0) Located more anteriorly than articular surface of condyle;
(1) Rounded and not protruded.
(2) Projecting ventrally;
(3) Projecting posteriorly.
(4) Squared and not protruding.
259) Mandibular foramen:
(0) Wide;
(1) Small.
260) Shape of mandibular foramen:
(0) Posteriorly concave;
(1) Triangular;
(2) Fissurated.
261) Gingival foramina:
(0) Absent;
(1) Present.
262) Mental foramina:
(0) Only one per dentary;
(1) Several mental foramina present per dentary.
263) Dentary curvature in dorsal view:
(0) Dentary with lateral concavity in dorsal view;
(1) Dentary straight;
(2) Dentary moderately bowed;
(3) Dentary strongly bowed.
264) External curvature in dorsal view:
(0) Absent;
(1) Continuous;
(2) Discontinuous.
265) Presence of dorsoventral curvature in dentary in lateral view:
(0) Absent;
(1) Present.
266) Types of dorsoventral curvature in dentary in lateral view:
(0) Absent;
(1) Continuous;
(2) Discontinuous.

267) Mylohyoidal groove:
(0) Absent;
(1) Present.
268) Crest along the ventral border of the dentary with a parallel groove:
(0) Absent;
(1) Present.
269) Medial face of dentary ramus:
(0) Flat;
(1) Convex.
(2) Concave.

VERTEBRAE
270) Cervical vertebrae:
(0) Free;
(1) Fused.
271) Cervical vertebrae:
(0) Elongated;
(1) Shortened.
272) Neural processes of cervical vertebrae:
(0) Free;
(1) Fused.
273) Dorsal process of C3:
(0) Present;
(1) Absent.
274) Dorsal process of C4:
(0) Present;
(1) Absent.
275) Dorsal process of C5:
(0) Present;
(1) Absent.
276) Dorsal process of C6:
(0) Present;
(1) Absent.
277) Dorsal process of C7:
(0) Present;
(1) Absent.
278) Ventral process of C3:
(0) Present;
(1) Absent.
279) Ventral process of C4:
(0) Present;
(1) Absent.
280) Ventral process of C5:
(0) Present;
(1) Absent.
281) Ventral process of C6:
(0) Present;
(1) Absent.
282) Ventral process of C7:
(0) Present;
(1) Absent;

(2) Reduced to a tubercle.

283) Foramen transversarium in C3:

(0) Complete;
(1) Incomplete.

284) Foramen transversarium in C4:

(0) Complete;
(1) Incomplete.

285) Foramen transversarium in C5:

(0) Complete;
(1) Incomplete.

286) Foramen transversarium in C6:

(0) Complete;
(1) Incomplete.

287) Foramen transversarium in C7:

(0) Complete;
(1) Incomplete.

288) Foramen transversarium

(0) Complete in C2;
(1) Incomplete in C2.

289) Fusion of sacral vertebrae:

(0) Present at least in part;
(1) Absent.

290) Number of sacral vertebrae:

(0) >1;
(1) 1.

291) Sharp lateroventral projection of transverse process:

(0) Present;
(1) Absent.

292) Foramen at emergence of transverse process:

(0) In caudal vertebrae;
(1) In last lumbar and caudal vertebrae.

SCAPULA

293) General proportions of scapula:

(0) High and narrow;
(1) Low and wide.

294) Orientation of scapular spine:

(0) Divergent from margo cranialis and directed dorsally;
(1) Parallel to margo cranialis and directed anterodorsally.

295) Development of teres fossa:

(0) Small;
(1) Enlarged.

296) Margo cranialis:

(0) Straight;
(1) Convex;
(2) Concave.

297) Inclination of margo cranialis with respect to horizontal axis:

(0) High;
(1) Scarce.

298) Margo caudalis:
(0) Straight-to-scarcely concave;
(1) Highly concave.
299) Development of supraspinous fossa:
(0) Wide;
(1) Reduced;
(2) Invisible in lateral view.
300) Scapular spine:
(0) Well developed:
(1) Reduced.

HUMERUS
301) Orientation of caput humeri:
(0) Along longitudinal axis of humerus;
(1) Located posteriorly to longitudinal axis.
302) Size of tuberculum majus:
(0) Small;
(1) Large.
303) Direction of tuberculum majus:
(0) Anteroposterior;
(1) Dorsal;
(2) Ventral.
304) Shape or margo ulnaris:
(0) Straight;
(1) Concave.
305) Shape of caput humeri:
(0) Flat;
(1) Highly convex.
306) Lateral edge of caput humeri:
(0) Straight;
(1) Forming a corner.
307) Orientation of lateral edge of caput humeri:
(0) Anteroposterior;
(1) Oblique (from a posterodistal to an anteroproximal position);
(2) Anteroposterior posterodistally and dorsoventral anteroproximally.
308) Lateral expansion of articular surface of caput humeri:
(0) Scarce;
(1) Well developed.
309) Deltopectoral crest:
(0) Present;
(1) Absent.
310) Tuberculum deltoideus:
(0) Highly relieved;
(1) Reduced;
(2) Absent.
311) Articulation with radius and ulna:
(0) Rotational;
(1) Non-rotational.
312) Position of ulnar epycondyle:
(0) High;
(1) Low;

(2) Almost absent.

313) Relative length of humerus:

(0) Longer than radius and ulna;
(1) Humerus length nearly equals that of radius and ulna;
(2) Much shorter than radius and ulna.

314) Proximal surface of tuberculum deltoideus:

(0) Continuous with deltopectoral crest;
(1) Concave;
(2) Straight and projecting posteriorly.

RADIUS

315) Proximal curvature:

(0) Massive;
(1) Reduced-to-absent.

316) Distal expansion:

(0) Absent;
(1) Present.

317) Proximal contact with ulna:

(0) Present;
(1) Absent.

318) Size of radius with respect to ulna:

(0) Anteroposterior diameter similar to that of ulna;
(1) Anteroposterior diameter larger than that of ulna.

ULNA

319) Olecranon: proximal corner:

(0) Directed proximally;
(1) Directed distally.

320) Olecranon: size:

(0) Well developed;
(1) Reduced.

321) Olecranon: dorsal and ventral borders:

(0) Parallel;
(1) Diverging posteriorly;
(2) Forming a right angle.

322) Olecranon: ventral angle:

(0) Right angle-to-obtuse;
(1) Acute.

323) Olecranon: posterior border:

(0) Squared;
(1) Round;
(2) Straight.

324) Proximal articular facet of ulna and upper side of olecranon:

(0) Forming a corner;
(1) Straight.

325) Distal expansion of ulna:

(0) Absent;
(1) Present.

MANUS

326) Articulation of carpals:
(0) Tight articulation;
(1) Loose articulation.

327) Digit number:
(0) Five;
(1) Four.

328) Hyperphalangy:
(0) Absent;
(1) Present.

329) Proportions of manus:
(0) Manus wide;
(1) Manus narrow.

330) Trapezium:
(0) Present;
(1) Absent.

331) Separate cartilagineous fields for trapezoid and unciform:
(0) Yes;
(1) No.

HINDLIMB

332) Pelvis articulated with vertebral column:
(0) Yes;
(1) No.

333) Massive reduction of pelvis size:
(0) No;
(1) Yes.

334) Functional hindlimbs in adults:
(0) Yes;
(1) No.

STERNUM AND RIBS

335) Number or ribs articulated to sternum:
(0) >1;
(1) 1.

336) First rib shape:
(0) Not expanded;
(1) Expanded.

337) Sternum formed by several sternebra:
(0) Yes;
(1) No, only by one manubrium.

338) Head of first rib:
(0) Bifid;
(1) Single.

339) Ribs with bifid head posterior to 5th:
(0) Yes;
(1) No.

340) Pachyosteoschlerotic ribs:
(0) Absent;
(1) Present.

DENTITION

341) Positions of upper premolars and molars:
- (0) Close to each other;
- (1) Well separated by diastemata.

342) Positions of lower premolars and molars:
- (0) Close to each other;
- (1) Well separated by diastemata.

343) Number of denticles on posterior upper teeth:
- (0) >3 along anterior or posterior borders;
- (1) 3 or less along anterior or posterior borders.

344) Dental generations:
- (0) Polyophiodonty;
- (1) Monophiodonty.

345) Heterodont teeth on dentary:
- (0) Present;
- (1) Absent.

346) Dentition reduced to a few anterior upper teeth:
- (0) No, complete dentition is present;
- (1) Yes.

347) Inferred or observed loss of mineralization in teeth (due to C4*orf* gene mutation):
- (0) Absent;
- (1) Present.

BALEEN

348) Inferred or observed presence of baleen:
- (0) Negative;
- (1) Positive.

349) Inferred or observed length of baleen:
- (0) Short;
- (1) Long.

350) Direction of baleen racks:
- (0) Limited to posterior part of rostrum;
- (1) Parallel;
- (2) Anteriorly convergent.

Taxon x character matrix used in phylogenetic analysis

Protocetidae
```
000-00000  0000000--0 0--------- -0-00-00-0 00-0000000 0000000000 0000000000
0000000-00 0000000000 0000000000 00000-0000 00000----0 -000000000 0-00000-00
----000--0 0000000100 000-0000-0 00000---00 00-------0 --0--00??? ???????0?0
000000000- 0?00000000 0000--0000 0000000000 00-0-00000 0000000000 0000000000
0000000000 0000000000 0????????? ?0?0?00000 0000000000 0000000000 000000000- -
```

Cynthiacetus peruvianus
```
000-00000  0000000--0 0--------- -0-00-00-0 00-1000000 0000000000 1000002000
0000000-00 0000000000 0000000000 00000-0010 00000----0 -000000000 0-00100-00
----000--0 1000000100 0000000000 00000---00 010????--0 --0--0???? ???????0?0
0001000010 0?00000000 0000--0001 0000000000 00-0-00000 0010000000 0000000000
0000000001 1000011000 0000000000 0000000000 0000000000 0011000000 000000000- -
```

Basilosaurus cetoides
```
000-00000  0000000--0 0--------- -0-00-00-0 00-1000000 0000000000 1000000000
0000000-00 0000000000 0000000000 00000-0010 00000----0 -000000100 0-00000-00
----000--0 0000000100 0000000000 00000---00 010000---0 --0--00000 00000000?0
0001000010 0000000000 0000--0001 0000000000 00-0-00000 0010000000 0000000000
0000000001 1000011010 0000000000 0000000100 0000000000 0011000000 000000000- -
```

Dorudon atrox
```
000-00000  0000000--0 0--------- -0-00-00-0 00-1000010 0000000000 1000000000
0000000-00 0000000000 0000000000 00000-0010 00000----0 -000110100 0-00000-00
----000--0 0000000100 0000000000 00000---00 010000---0 --0--00000 00000000?0
0001000010 0000000000 0000--0001 0000000000 00-0-00000 0010000000 0000000000
0000000001 1000010010 0000000000 0000000000 0101000000 0011000000 000000000- -
```

Zygorhiza
```
000-00000  0000000--0 10000-00?0 00-00-00-0 00-1000000 0000000000 1000001000
0000000-00 0000000000 0000000000 00000-0010 00000----0 -000110100 0-00000-00
----000--0 1000000100 0000000000 00000---00 010000---0 --0--00000 00000000?0
0001000010 0000000000 0000--0001 0000000000 00-0-00000 0010000000 0000000000
0000000001 10000?2010 00??0????0 0000000100 0000000000 0011000000 000000000- -
```

Mammalodontidae
```
010-10100  1000011000 10000-0?00 0100111000 00-3100000 0000010000 1000002001
0100--0-00 1000000010 0?20000?00 00000-0000 00000----0 -0?0111110 1-10000-11
----100--0 1000000100 ?011??00?0 ?000???01 ?10000---1 000--00100 000?0?10?0
00010?000- 0000000000 0000--000? 0000000000 00-0-00100 0011000000 0000??????
?????????? ?????????? ?????????? ?????????? ?????????? ???????0?? ?00100000- -
```

Fucaia buelli
```
010-10001  1010111001 10000-0?01 0100101000 0102000120 0000010000 0000002000
0101000-00 1000000010 0020000000 01000-0000 00000----1 00?0111110 1-10000-11
----10110? 1000000100 ?011000010 ?000101000 3110122001 000--010-0 000?0?00?0
00010?0010 0000000000 0000--0001 0011011010 00-0-00100 0011000000 0000000000
01000011?? 1?0?1000?0 0????????? ?????????? ?????????? ??????1??? ?01000000- -
```

Aetiocetus weltoni
```
010-10001  1010111001 10000-0?01 0100111000 10-2001120 0000000000 0000002002
0100--0-00 1000000010 0000000000 02000-0000 00000----1 0000111110 1-1000??11
----100--? 1000000111 1011000010 1000101?0? ?????????? ?????????? ??????????
?????????? ??00000000 0000--0001 0001011010 00-0-00100 0011000000 ??????????
?????????? ?????????? ?????????? ?????????? ?????????? ?????????? ?111110010 0
```

Yamatocetus canaliculatus
```
110-11101  1110211001 10000-0?01 1110110000 0101000120 0000020000 0000001001
0000--0-00 1000000010 0111000000 02001-0000 00000----1 0??0111110 1-10001012
```

```
----100--? 1000010111 ?110020000 0000????0? ?????????? ?????????? ??????????
?????????? ??00000000 0000--0000 0001111010 00-0-00100 0111000000 0000000000
000000001? ???1011100 0000000000 0100000100 011100???? ????????00 0------010 0
```

Eomysticetus whitmorei

```
110-1?101  11102110?1 0------?-- -??0??0001 0??1000120 0000010000 0000000000
0000--0-00 1000000010 0011000000 02001-0000 00000----1 0000111110 1-10001012
----100--? ?100010111 111????000 00000---01 311210---0 --0--01001 001?0?00?0
00010?000- 0100000000 0000--0000 0001111010 00-0-00100 0111000000 000?000000
000?????1? ?0?1????1? ?00-000000 0100010100 000100???? ?????????? ?------010 0
```

Micromysticetus rothauseni

```
?10-?????  ?????????? ?????????? ?????????? ?????????? ?00??????? ???000?0??
??0?--0-00 1?00000010 0011000000 02001-0000 00000----1 0000111110 1-10001012
----100--? ?100010111 11100??000 0000100001 311210---0 --0--01010 001?0?00?0
00010?000- 01???????? ?????????? ?????????? ?????????? ?????????? 000???????
?????????? ?????????? ?????????? ?????????? ?????????? ?????????? ???????0?? ?
```

Waharowa ruwhenua

```
110-11000  1110211001 ?????????? ???0????0 0??2000120 0000021000 1000001000
0000--0-00 1000000010 0011000000 02001-0000 00000----1 0??0111110 1-10001012
----100--? ?1000??011 111102?000 0000100001 311210---1 000--00010 0010000?0
0001000010 0100000000 0000--0000 0001111010 00-0-00100 0111000000 000?0????0
?????????? ?0?0110011 1???0????0 0110010100 011100???? ?????1?000 0------010 ?
```

Sitsqwayk cornishorum

```
110-1?000  1110211001 0------?-- -1100-0000 0111?0?120 0000020021 0010000001
0100--0-00 1000000010 1?10000000 0101000011 00000----1 0??0001110 1-10001002
----100--? 0110110111 111002?10? 0000????0? ?????????? ?????????? ??????????
?????????? ??00000010 0000--000? ?0?1111010 00-0-00100 0111000000 0000000000
0011?1111? ?0?1011100 010-011210 0100010100 011000???? ?????0?000 0------010 ?
```

Horopeta umarere

```
?10-????1  111??????? ?????????? ?????????? ?????0?120 000?0????? 1??000100?
??00--0-00 1?00000010 ??????0000 0?0???0??? 00000----? ???000?11? 1-10000-01
----100--? ??????0111 11100????? ?000100001 311110---1 000--00010 01200?11?0
00010?0010 0100000000 0000--0000 001111???0 1100-????? ?111000000 0000000000
001???10? ???0100011 0????????? ?????????1 ?01000???? ?????0?000 0------010 ?
```

Morenocetus parvus

```
????????1  ?????????? 10000-1?11 0????????? ???????0?0 1111000020 0111011100
030???0-00 101-13-011 3211000000 0?11000100 21210----1 0000001131 0-01000002
----000--? ?111100111 111011?101 0120112101 ?110?0---0 --0--00??? ????00??0?
??001?0010 0????????? ?????????? ?????????? ?????????? ?????????? ??????????
?????????? ?????????? ?????????? ?????????? ?????????? ?????????? ?--?-???11 ?
```

Caperea marginata

```
001011011  1110111011 10000-1111 0100111001 10-3212020 1121000020 0111011101
0300--0010 101-13-011 3201000000 0011000100 21210----1 0000001131 0-01011012
----300--1 0111010111 1111--0101 0120112101 111011---0 --0--001-0 01210?101?
0100100010 0011110001 1101001111 1001111013 00-0-10211 0111011100 1111111101
1111111111 1111111111 1110111211 1112110101 0010101111 0111111110 1---11-011 1
```

Miocaperea pulchra

```
001011011  1110111011 10000-1?11 0100111001 1103212020 1121000020 0111011100
0300--0010 101-13-011 3221000000 0011000100 21210----1 0200001131 0-01011012
----200--? 0111000111 1111--0101 0120112101 1110??---0 --0--001-0 ??????????
??001?0010 0????????? ?????????? ?????????? ?????????? ?????????? ??????????
?????????? ?????????? ?????????? ?????????? ?????????? ?????????? ?-?-1?-011 1
```

Balaenella brachyrhynus
101011011 1100111201 10000-1?11 0100111001 1??3???0?0 1110100020 0111011101
0301100010 101-13-111 3111000100 0301011100 21210----1 1100001131 0-21011002
----300--? 0111100111 1111--1101 0020112101 1110?0---0 --11101??? ?????0????
??001?0010 ???1110001 110100111? ?011?????? ?????????? ?????????? ??????????
?????????? ?????????? ?????????? ?????????? ?????????? ?????????? ?-?-1?-011 1

Balaena mysticetus
101011011 1100111201 10000-1111 0100111001 1113212020 1110100020 0111011101
0301100010 101-13-111 3111000100 0301011100 21210----1 1100001131 0-21011002
----300--1 0111100111 1111--1101 0020112101 111010---0 --11101000 012000000?
0100100010 0011110001 1101001111 1011111113 00-0-10211 011311?100 1110000000
1111111111 1110000000 0110110111 1121111000 1000011010 0111111110 0---11-011 1

Balaenula astensis
10111??11 1100?11211 10000-1?11 0100111001 11?3001020 1110110021 0101011101
0301010110 111-13-011 3111000000 0011011100 21210----1 1100001131 0-01011001
----200--? 0111100110 1110131101 0020112101 111010---0 --10001000 012?00?00?
01001?0010 0011110001 110100111? ?011111113 00-0-10211 0111112100 ????000??
?????????? ?????????? ?????????? ?????????? ?????????? ?????????? ?---11-011 1

Eubalaena spp.
101111011 1100111211 10000-1111 0100111001 1113001020 1110110021 0101011101
0301010110 111-13-011 3111000100 0301011100 21210----1 1100001131 0-01011001
----200--1 0111100111 1110131101 0020112101 111010---0 --10001000 212000001?
0100100010 0011110001 1101001111 1011111113 00-0-10211 0113112100 1110000010
1111111111 1110000000 0110110111 1121111110 1000011010 0111111110 0---11-011 1

‘Aglaocetus’ patulus
110-11111 1110111101 11000-0?01 1100111001 10-3101030 1111110000 0111101001
0200--0-11 0001000010 1011000100 0301000010 11100----1 0110001121 1-11000-02
----100--1 0111110111 1110130100 1010112100 311011---1 000--01010 012?0?00?0
10010?0010 0011110110 0111100001 001??????? ?????????? ?????????? 0100?0??0?
0??1?????1 11???????? ?????????? ?????????? ?????????? ?????????? ?---11-110 2

Pelocetus calvertensis
110-11111 1110211101 11000-0?01 1100101001 10-3?01031 1110121000 0111101001
0200--0-11 0001000010 1011000100 0301000010 11100----1 0110001121 1-11000-03
----100--1 0111110111 1110130100 1020112100 311??????? ??0--01??? ??????????
?0010??010 ??21110110 0111100001 0011111012 1100-00120 ?113100001 010?0?00?0
?02?1??101 11?1011110 0110011211 212?-????? ????010?? ????11110 0---11-110 2

Uranocetus gramensis
110-11111 1110211101 11000-0?01 1100101001 10-3101131 1111110000 0111101001
0200--0-11 0001000010 1011000100 0301000110 11100----1 0110001121 1-11000-14
0010100--1 0111110111 11101?0100 1020112100 311??1---1 000--01010 012?0?00?0
10010?0010 0?21110110 0111100001 00111??12 1100-00120 1113000001 100000000?
?100???01 1??0102011 0110011211 ?122?10100 011100???? ?????????? ?---11-110 2

Isanacetus laticephalus
110-11111 1110211001 11000-2?01 1100111011 10-3101021 1111121000 0111101001
0200--0-11 0001???010 1011000100 1301000110 11100----1 0110001121 1-110?????
????100--1 01111?01?? ?1110-0100 1010112100 311011---1 000--01010 012?0?00?0
10010?0010 0111110110 0111100001 00111???1? ?????????? ?????????? ??????????
?????????? 1????????? ?????????? ?????????? ?????????? ?????????? ?---11-110 2

Joumocetus shimizui
110-11111 1110011001 11000-0?01 1100111011 10-3101131 1111220?0 0111101001
0200--0-11 0001001010 10??????00 ??01??0000 11100----1 0110001121 1-1100???4
0010100--1 01111?0111 11110-0100 1010112100 ?1???1---? 000--0???? ??????????
?0010????? ??11110110 0110--0001 ?01111101? ?????0013? ?112000001 ??????????
?????????? 1????????? ?????????? ?????????? ?????????? ?????????? ?---11-110 2

Parietobalaena palmeri

```
110-11111  1110??1001 11000-2101 11001?10?1 10-3101021 1111112000 0111101001
0200--0-11 0001000010 1011000000 1101000100 11100----1 0110011121 1-11000-14
0010100--1 0111110111 1110130100 1010112100 310001---1 000--01010 0121111?0
1001000010 1111110110 0110--0001 0011111012 1100-00120 1111000001 0100000000
0??1?????1 11???????? ?????????? ?????????? ?????????? ?????????? ?---11-110 2
```

Parietobalaena campiniana

```
110-11111  1110111001 ?????????? ?100111001 10-??????? 1111122020 011??????1
02???-?-?? ?0?????010 1011000000 1101000100 11100----1 0110001121 ??????????
?????????1 ?111100111 111??30100 1010112100 ?10001---1 000--01010 012?1?11?0
10010?0010 1111110110 0110--0001 0011111012 1100-00120 1111000001 0100000000
112??111?1 1????????? ?????????? ?????????? ?????????? ?????????? ?---11-110 2
```

Tiucetus rosae

```
110-11??1  111??????1 110?0-0?02 2100??10?1 1??3000121 111????-?? 0?11101001
0200--0-11 0001111010 1011000000 0101000100 1110100?1 0110011121 1-11101014
0010100--1 0111100111 1110100100 1110112100 ?11???---1 000--01??0 ??????????
?0010??010 ??11110110 011???0001 001??????? ?????????? ?????????? ??????????
?????????1 1????????? ?????????? ?????????? ?????????? ?????????? ?---11-110 2
```

Diorocetus hiatus

```
110-11111  1110110110 11010-0?01 2100111001 10-2001030 1111020000 0111100001
0200--0-00 1002000010 1011000100 0101001100 11100----1 0010001121 1-12101-14
0011000--1 0111110111 1110130100 0010102000 311013---1 000--01010 212?1?11?0
10010?0010 1111110100 0110--0011 0011111012 1100-00131 ?111000001 01?????0??
??2????101 1101012010 0????????? ?????????? ?????????? ?????????? ?---11-110 2
```

USNM187416

```
110-11?11  1110110110 10000-0?02 2100111001 10-2001020 1111010021 0111101001
0200--0-00 1002000010 1021000100 0101001100 11100----1 00?0001121 1-12101-14
0001000--1 0111110111 1110130100 0120112000 ?????????? ?????????? ??????????
?????????? ??111101?? 0111010011 01???????? ?????????? ?????????? ??????????
?????????? ?????????? ?????????? ?????????? ?????????? ?????????? ???-11-110 2
```

Mixocetus elysius

```
110-11111  1110110110 1111002?01 1100100001 10-3001120 1121021002 0210102001
0210--1-00 1002111010 202100000? 0301000001 1100?----1 0010001120 0-22110-01
----000--1 0111110111 1110110100 ??22?????? ?????????? ?????????? ??????????
?001?????? ?????????? ?????????? ?1?1111012 ???0-00211 ?11?000000 01?0000000
000???00?? ?1?1?1?111 1????????? ?????????? ?????????? ?????????? ?---11-110 2
```

Cetotherium rathkei

```
100-1???0  111??10110 1111102?02 2???10?001 1103101130 1121021021 0210102001
0210--0-00 1002111010 2022000001 0301000000 11000----1 00?0001120 1-12100-14
0111000--1 0111010111 1111--0110 0????????? ?????????? ?????????? ??????????
?????????? ??20110l?? 0111000011 01???????? ?????????? ?????????? ??????????
?????????? ?????????? ?????????? ?????????? ?????????? ?????????? ?-?-11-110 2
```

Cetotherium riabinini

```
100-11?10 1110110110 1111102?02 210011?001 10-2211120 1121010001 0210102001
0210--1-00 1002111010 2022000001 0301000000 11000----1 0110001120 1-12100-14
0011000--1 0111110111 1110120100 0120????0? ?????????? ?????????? ??????????
?????????? ??201101?? ???0--00?1 01?1111012 1100-10021 2111000001 010?0001?0
002?0001?1 1000110002 111101???1 1112-10100 011100???? ??111????1 1---11-110 2
```

Metopocetus durinasus

```
??0-1???0  111??????0 111?100?02 2?????0??? ???3???130 11???????? ???010200?
?210--1-00 1002111010 ????000??? 0301000101 11?00----1 0??0??112? 1-12100-14
0111000--1 ?111?10111 11101??100 ????????0? 311113---1 000--00010 012?1?11?0
10010?0010 10???????? ?????????? ?????????? ?????????? ?????????? ??????????
?????????? ?????????? ?????????? ?????????? ?????????? ?????????? ???????1?? ?
```

Metopocetus hunteri
```
??0-1???0  111??????0 111?000?02 2????????? ???????1?0 1121?????? ???010200?
?210--1-00 1002111010 2022000001 0301001101 11?01000?1 0000001120 1-12100-11
----100--1 ?111110111 1110120100 011010201? ?1111????? ???????010 012?1?11??
?0000?0010 1021110110 0110--0001 011??????? ?????????? ?????????? ??????????
?????????? ?????????? ?????????? ?????????? ?????????? ?????????? ???????1?? ?
```

Piscobalaena nana
```
100-11110  1110110110 1111110?02 2100111001 1104001130 1121021000 0210102001
0210--1-00 1002111010 2022000101 0301000000 11000----1 0110001120 0-22100-11
----200--1 01110101?? ?111--0100 0100102000 311113---1 000--00010 012?1?1110
00010?0010 1101110110 0110--0001 0111111012 1100-00131 2112000001 0100000000
0020001101 1101011100 0111011211 2111210100 011001???  0?11110110 0---11-110 2
```

Herpetocetus morrowi
```
100-11??0  111?110110 1111112?02 2100111001 10-3001130 1121022001 0210102001
0210--0-00 0002111010 1022000001 0001002100 11000----1 0100001120 1-12100-14
0012000--1 0111110111 1110020110 1100102010 311113---1 100--01010 012?1?1110
10010?0010 1001110110 0110--0001 0111111012 1100-00130 1112000001 0?????????
????????01 110??????? ?????????? ?????????? ?????????? ?????????? ?---11-110 2
```

Herentalia nigra
```
??0-1???0  111??????0 111?000?02 2????????? ???????120 1121?????? 021010200?
?210--1-00 1002111010 ?????????? ???1??0001 11?01001?1 0000?0112? 0-02101011
----200--1 ?111010111 1110220100 0120102000 ?1111????1 000--?1010 ??????????
?00??????? ?????????? ?????????? ?????????? ?????????? ?????????? ??????????
?????????? ?????????? ?????????? ?????????? ?????????? ?????????? ???????1?? ?
```

Thinocetus arthritus
```
??0-1????  ?????????? ?????????? ?????????? ?????????? ?????????? ??????????
???????-?? ?????????0 ?????????? ?????????? ?????????? ??????1??? ??????????
?????????? ????010111 111???0100 ??20112010 31111????1 000--00100 01??1?11?0
00010?0010 1011110100 0111000011 011??????? ?????????? ?????????? 010000??00
0??0????01 1101110111 0112011211 1112210100 011100???? ????10110  ???????1?? ?
```

Halicetus ignotus
```
??0-1????  ?????????? ?????????? ?????????? ?????????? ?????????? ??????????
???????-?? ?????????0 ?????????? ?????????? ?????????? ??????1??? ??????????
?????????? ?????????? ?????????? ??10112000 31101????0 100--01100 01??1?11?0
00010?0010 1011110100 0111010011 011??????? ?????????? ?????????? 0100000000
0110111101 110??????? ?????????? ?????????? ?????????? ?????????? ???????1?? ?
```

Titanocetus sammarinensis
```
110-11010  1110110110 11100-2?00 0100100001 10-3001100 1111120002 0100102001
0210--0-00 1001111010 1011000001 0301000011 11100----1 0??0001120 0-02100-01
----200--1 0111010111 1110120100 01???????? ?????????? ?????????? ??????????
?????????? ?????????? ?????????? ?1?1111012 1100-10120 1113100000 010???????
????????1? ?????????? ?????????? ?????????? ?????????? ?????????? ???????1?? ?
```

Cophocetus oregonensis
```
110-11111  1110110110 11010-2?01 1100100001 10-3001120 1111121002 0210102001
0210--0-00 1002111010 1022000100 0101000010 11110----1 0011001121 1-12100-04
0101000--1 0111010111 1110110100 0020?????? 311013---1 000--00100 012?0?01?0
10010?0010 0111110100 0110--0011 00111??012 1100-20120 2112100001 0100000000
011????01  1101111002 011?011211 2122-????0 010100???? ?????????? ?---11-110 2
```

Aglaocetus moreni
110-11110 1110110110 10000-2?01 1100110001 10-3001100 1111110001 0110102001
0210--0-00 1002111010 1011000100 0101000010 11110----1 0011001121 1-12100-04
0101000--1 0111010111 1110110100 0020?????? ?????????? ?????????? ??????????
?????????? ?????????? ?????????? ???1111012 1100-0012? ?112000001 ??????????
?????????? ?????????? ?????????? ?????????? ?????????? ?????????? ?---11-110 2

Eschrichtius robustus
101011011 1110211001 10000-2211 0100111001 10-4102120 1121110021 0220102101
0211--0-00 1002121010 2122000101 0301000001 2111111?21 0101001120 2-22101114
11103010-1 0111110111 1110100110 1020???02 3101120001 000--111-0 0121101?10
101111100- 1211111000 0111000010 0111111013 00-1110131 2112011000 0100000000
0001111101 1100110001 1112011211 1111211100 0201011111 ??11111110 0---11-110 2

Eschrichtioides gastaldii
110-11??0 1110?????1 10000-2?11 0100??1001 10-3101120 1121110011 0220100001
0211--0-00 1002121010 2111000101 0301000101 2101100?1 01?1001120 0-02101101
----00???1 0111100111 111???0110 ?????????? ?????????? ?????????? ??????????
?????????? ??10111000 0111000010 0111111013 00-1110131 2112011000 010???????
????????1? ?????????? ?112011211 2121-????0 020200???? ??111????? ?---11-110 2

Archaeschrichtius ruggieroi
????????? ?????????? ?????????? ?????????? ?????????? ?????????? ??????????
???????-?? ?????????? ?????????? ?????????? ?????????? ?????????? ??????????
?????????? ?????????? ?????????? ?????????? ?????????? ?????????? ??????????
?????????? ?????????? ?????????? ???1111012 11010????? ?112011002 ??????????
?????????? ?????????? ?????????? ?????????? ?????????? ?????????? ?---11-11? ?

RBINS M. 2231
110-????? 111???1001 10100-??1? ??????1?1? ???????1?0 1230020022 0320102011
131???1-11 1003121010 ???????01 0?01000000 2121101?1 0110001121 0002100-01
----200--? ?111110111 111011?1?? 1020112100 1110020011 110--11010 012?0?00?0
11111?110- 0131111100 0101100001 0011111012 1100-21331 2113100002 010????0??
??????11? ???1110002 1112011211 2112-10?00 01120?1??? ?????????? ?---11-110 2

'Balaenoptera' ryani
????????? ?????????? 1???0-???2 0????????? ???????0?0 1231?0???2 011110100?
?210--0-01 1002121010 ?????????? ???1??1100 21?11000?1 010?00112? 1012101004
1110100--? ?11??10111 111020?110 1????????? ?111120011 000--11010 012?0?01?0
00011?1010 01????11?0 01?110000? ??0??????? ?????????? ?????????? ??????????
?????????? ?????????? ?????????? ?????????? ?????????? ?????????? ???????1?? ?

Protororqualus cuvieri
110-11010 1110211001 10100-2?12 1100111111 10-4101120 1242101012 0320102011
0220--1-11 1003021010 1?11000??? ??01002100 2121?????1 01?1001121 1012101014
0201100--? 0111100111 111???0110 ?????????? ?????????? ?????????? ??????????
?????????? ??3111110? ?10??000?1 01?1111012 1100-21241 ?111000000 0100????0?
???????01 11?111?112 1112001211 1121210100 0101001??1 ??1111???? ?---11-110 2

RBINS M. 2315
??0-????? 11???????? 101?0-2?12 2??0?11??? ???????1?? 12421???11 0??0102012
122???1-11 1003021010 ???1?10101 0?01001000 2121110?1 010100112? 10121????4
0201100--? ?111110111 111010?1?0 1120112100 ?111120001 100--001-1 112?0?01??
?1110?1010 0131111100 1101100001 011??????? ?????????? ?????????? ??????????
?????????? ?????????? ?112?11211 ?1???????? ?????????? ?????????? ???????1?? 2

UT PU13842/5
110-11112   1111211??1 10100-1?12 1100??1111 10-4201120 1242200012 0320102012
1211--1-11 1003021010 1121010101 0101001100 2121?????1 01?1001121 1012101014
0201110--1 0111100111 111010?110 1?2??????? ?111120001 100--011-1 112?0?01?0
21110?0010 0131111100 1101100001 0111111012 1100-21241 2112100000 0100000000
0021111101 1101112112 1110010001 2122-10100 011100???? ??11111111 0---11-110 2

'Balaenoptera' cortesi var. *portisi*

```
100-11112  111011???1 10100-0?12 2111??1??1 1??????130 1231100011 0321102111
122???0-11 1002121010 1011100111 0100000100 2121112230 1?00011213 1021111004
1212100--? 0111110111 1111--0110 1120112100 ?111?20011 110--00??? ??????????
?1110??00- ??31111110 010??0000? ?1?1111012 1100-21131 2112000000 0100000000
000?????1? ???1112112 1112011211 11122????1 001200???? ?????????? ?---11-110 2
```

NMR 7096

```
??0-1?112  1110?????? 10100-0?12 2101?????1 ???????130 1231?????? ???110201?
?221--1-11 1002121010 1211100012 0100000100 211211123? 1100001121 3012110-04
1212100--? ?111110111 11100??1?0 1020102100 1101020?1 110--00010 112?1?11??
?1110?000- 0131111110 010110000? ?11??????? ?????????? ?????????? ??????????
?????????? ?????????? ?????????? ?????????? ?????????? ?????????? ?-?-1?-110 2
```

MPTAM 207.13307

```
110-110?1  1110??10?1 10100-2?12 210???1111 1??4010130 1232100012 0320102011
1210--1-11 1003121010 2011000001 0301001100 2121110210 1000011212 2022101004
1210300--? ?111110111 111011?1?0 1110112100 1111120001 110--001-0 0???1?11??
?1110?100- 01???????? ?????????? ?????????? ?????????? ?????????? 0100000000
002111110? ?????????? ?????????? ?????????? ?????????? ?????????? ???????1?? 2
```

Plesiobalaenoptera quarantellii

```
110-11111  11112110?1 10100-2?1? 1100111?11 1????????? 12?21????? ???01?????
??1????-11 1???121??? ?????????? ?????????? ?????????? ???????12? 2022010-04
01030????? ?????1011? ?????????? ??21112000 1111020011 110--00010 012?1?11?0
21110?0011 1130111100 1101100001 0011111012 1100-21341 0113100002 01000???00
???11????1 11???????? ?????????? ?????????? ?????????? ?????1111? ?---11-110 2
```

SAM55001

```
????????? ?????????? ?????????? ?????????? ?????????? ?????????? ??????????
???????-?? ?????????0 ?????????? ???0?????0 ?????????1 010100???? ??????????
?????????? ?111?????? ???????1?? ?????????? 1110200111 010--00010 012?1?11?0
21110?0011 1030111100 1101100001 0?1??????? ?????????? ?????????? ??????????
?????????? ?????????? ?????????? ?????????? ?????????? ?????????? ???????1?? 2
```

Parabalaenoptera baulinensis

```
110-11111  1110??1001 10100-2?11 2100111?01 1??3001120 1232201022 0320102011
?220001-11 1003121010 1021100001 0301001100 21211?2??1 01?000?121 0022101014
1100210--? 0111??011? ?????????? ?????????? ?????????? ?????????? ??????????
?????????? ?????????? ?????????? ???1111012 1110-21241 0113000002 0100????0?
???1????01 11???????? ?????????? ?????????? ?????????? ?????????? ?---11-110 2
```

Fragilicetus velponi

```
??0-?????  11???????? 10100-2?11 2????????? ???????110 12322???22 0??010201?
1221--1-11 1003121010 10110?0000 0301000101 11201122?1 0100001121 2022101014
1110300--? ?111110111 111111?1?0 1120112100 1111200?1 110--001-1 112?1?11?0
11110?0010 11???????? ?????????? ?????????? ?????????? ?????????? ??????????
?????????? ?????????? ?????????? ?????????? ?????????? ?????????? ???????1?? 2
```

'Megaptera' hubachi

```
110-11010  1110111001 10100-2?11 1100111111 1??3011120 1232101021 0320102011
1210001-11 1003121010 1021000000 0101000100 11211100?1 0100001121 2022101004
1110300--? 0111110111 11?11101?0 1021112002 1111?2000? ??0--00??? ????1?11?0
21110?0011 11???????? ?????????? ???1111012 1100-21241 ?113000002 0100000000
0021111101 1101110111 1110011211 2112110100 0111001111 0111111111 0---11-110 2
```

Incakujira anillodefuego

```
110-11111  1110311101 10100-2?11 1100111111 10-4011130 1242200112 0320102012
1210001-11 1003121010 1011110111 0201001100 21211????1 01?0001121 2022101012
----301101 0111110111 1110210110 1121?????? ?11??20??1 100--00??? ??????????
?1110?001? ??31111110 1101100001 0111111012 1100-2134? ?112000?0? 0100000000
00?0????01 1111111110 1110011211 2122110100 0111001111 01111?1?11 0---11-110 2
```

MHNL 1613
```
110-11111  1110311101 10100-2?12 1100111111 10-3011130 1242202122 0320102012
1211--1-11 1003121010 1011110112 0101000100 2121100?1 0010001121 2022101012
----30110? 0111110111 11102001?0 102??????? ????????? ????????? ?????????
????????? ????????? ????????? ?1???????? ????????? ????????? ?????????
????????? ????????? ????????? ????????? ????????? ????????? ?---1?-110 2
```

Archaebalaenoptera castriarquati
```
110-1?110  1110111101 10100-2?12 1100111111 ???3000120 1242100022 0320102011
1221101-01 1013121010 11110?0102 0301001100 1121?????? ???000?121 3022101014
121001100? 0111101??? ????????? ?1???????? ????????? ????????? ?????????
????????? ????????? ????????? ???1111012 1100-212?? ?111?????? ?????????
????????? ????????? ????????? ????????? ????????? ????????? ?---11-110 2
```

MHNL 1610
```
110-1?012  1110111101 10100-2?12 2100111111 ???4010120 1242100021 0320102012
?221--1-11 1003121010 1011010102 0101001100 2121?????? ???000?121 3022101014
1210011001 0111?101?? ????????? ????????? ????????? ????????? ?????????
????????? ????????? ????????? ???1????1? ????????? ?111?????? 0100000??
????????0? ????????? ????????? ????????? ????????? ????????? ?---11-110 2
```

MAB002286
```
??0-?????  ????????? 10??0-2??2 2????????? ???????1?0 1242?????? ???010201?
1221--1-01 1003121010 ???????02 ???1001100 21?11003?? ??0000112? 3022101014
121001100? ?111110111 111020?1?0 1121102100 ?11??200?1 110--10010 012?0?01??
?1110????? ????????? ????????? ????????? ????????? ????????? ?????????
????????? ????????? ????????? ????????? ????????? ????????? ???????1?? 2
```

Nehalaennia devossi
```
?10-11??0  111?111001 10100-1?12 2100111111 10-3010130 1232102121 0320102011
1310001-11 1003121010 202200000? 0101001100 21211003?? ???0001121 0022101012
----01100? 0111110111 1111--?1?0 1110112100 ?111120001 110--001-0 ?????????
?1110?0??? ????????? ????????? ????????? ????????? ????????? 010???????
????????? ????????? ????????? ????????? ????????? ????????? ?---11-110 2
```

‘Balaenoptera’ bertae
```
??0-?????  ????????? 1???0-???? 2????????? ???????1?? 1231100110 0320102011
121???1-11 1003121010 2012000101 0201001100 2121100??? ???1001121 2022101004
0111300--? ?111110111 11?????1?0 1110112100 ????????? ????????? ?????????
????????? ????????? ????????? ????????? ????????? ????????? ?????????
????????? ????????? ????????? ????????? ????????? ????????? ???????1?? 2
```

Shimajiri-kujira
```
??0????01  ??????1110 1?100-???? 2???111111 ???????1?? 1232100122 0130122011
121???1-11 1003121000 2011000101 0201001100 2121?????? ???1001121 ?0?2101012
----?00--? ?111110111 11????01?? ?1???????? ?11??20001 010--00??? ?????????
?1110?0010 ??3101110? ???110?00? ?0???????? ????????? ????????? ?????????
????????? ????????? ????????? ????????? ????????? ????????? ???????1?? 2
```

Maesawa-cho
```
110-11011  1110?11101 10100-??12 1100111111 10-4102130 1232200?20 0320102012
?211--1-11 10031210?0 1211010001 0201001100 2121?????1 ????00?121 0022110-04
1010200--? ?11111011? ????????? ????????? ????????? ????????? ?????????
????????? ????????? ????????? ???1111012 1100-212?? ?111000??? 010???????
????????? ???1?1???? ????????? ????0????? ????????? ????????? ?---11-110 2
```

Miobalaenoptera numataensis
```
?????????  11????1101 ?????-???? ????????1? ????????? 1231100120 0320102012
121????-?1 1003121010 1011110111 0101001100 2121?????1 01?0001121 00221?????2
----000--? ?111110111 111????1?0 ??201121?? ?111120001 100--001-0 012?1?11?0
11111?1010 01???????? ????????? ????????? ????????? ????????? ?????????
????????? ????????? ????????? ????????? ????????? ???????1?? ???????1?? 2
```

Diunatans luctoretemergo

```
????????1  ?????????? 101?0-1?11 0????????? ???4012120 1232?????? ??20120 1??
130???1-01 1003121010 2022000001 0101000100 2121111??1 0100001121 2002110-04
1110300--? ?111110111 111020?1?0 1120112100 ?111120011 110--001-1 011?1?11??
?1111?0010 0131111111 110???0000 001??????? ?????????? ?????????? 0100???00
?????????? ?????????? ?????????? ?????????? ?????????? ?????????? ??????1?? 2
```

Megaptera miocaena

```
?10-?1??1  111??11001 10000-2?21 0100111111 10-3011120 1232110022 0320102012
1311--1-11 1003131010 1211010001 0201001100 21211????1 01?0001121 0002110-02
----000--? 0111111111 1111--0110 1?2??????? ?111120011 1?0--001-0 012?0?00?0
21111?000- 1011111100 1110001101 1????????? ?????????? ?????????? ??????????
?????????? ?????????? ?????????? ?????????? ?????????? ?????????? ?-?-?1?110 2
```

'Balaenoptera' siberi

```
110-11011  1110211101 10100-1?22 1100111111 1??41?2130 1232202121 0320112112
?300011--1 111-131010 1?22010?01 0?01001000 2121?????1 0???001121 0002110-02
----000--1 011111?111 11???????? ?????????? ?????????? ?????????? ??????????
?????????? ?????????? ?????????? ??11111111 00-0-212?? ?111?????? ?10???????
?????????? ?111110111 1110011211 1122010100 0111001111 1111111??? ?---11-110 2
```

Megaptera novaeangliae

```
110-11011  1110211101 10100-2211 0100111111 10-4010120 1232202121 0320102012
1300011-11 0003131010 1211010101 0201001100 2121112301 0111001121 0022100-14
1010310--1 0111101111 1111--0110 1021112102 3110120011 110--101-0 012?0?00?0
11111?000- 0130111110 1101100001 0011111013 1110-21341 2113200011 0100011111
0001111101 1111112111 1112011211 1122011100 1112111111 1111111111 0---11-110 2
```

Balaenoptera musculus

```
110-11011  1110211101 10100-1222 0100111111 10-4011120 1232200020 0320112112
1301--1-11 011-131010 1211011101 0201001100 2121112371 0111001121 2102100-04
1110310--1 0111101111 1110210110 1021112102 1111020011 110--10010 112?0?00?0
11111?110- 0130111101 1101100001 0011111012 1110-21341 2113200012 0100000000
0020001101 1111112111 1110011211 1112010100 0000001111 1111111111 0---11-110 2
```

Balaenoptera physalus

```
110-11011  1110111101 10100-1222 0100111111 10-4010130 1232202021 0320102012
1301--1-11 0003131010 1221011101 0201002100 2121112321 0111001121 2102100-14
1110310--1 0111101111 1110210110 1021112102 1110120111 100--011-1 0121010010
111101000- 0030111101 1101100001 0011111012 1110-21341 2113200012 0100000000
0021111101 1111112111 1110011211 1112010100 0111001111 1111111111 0---11-110 2
```

Balaenoptera acutorostrata

```
110-11011  1110111101 10100-1222 0100111111 10-3202130 1232200020 0320102012
1300011-11 0003131010 1221011101 0201002100 2121112331 0111001121 0102100-14
1110311101 0111101111 1110210110 1011112102 1110120011 000--111-0 0121010000
111111000- 0130111101 1101100001 0011111012 1110-21341 2113200012 0100000000
0020101101 1111112111 1112011211 1112010100 0111001111 1111111111 0---11-110 2
```

Balaenoptera bonaerensis

```
110-11011  1110111101 10100-1222 0100111111 10-3202130 1232200020 0320102012
1300011-11 0003131010 1221011101 0201002100 2121112331 0111001121 0102100-14
1110311101 0111101111 1110210110 1011112102 1110121111 000--011-0 012?0?0000
11111?000- 0130111101 1101100001 0011111012 1110-21341 2113200012 010?000000
0020101101 1111112111 1112011211 1112010100 0111001111 1111111111 0---11-110 2
```

Balaenoptera omurai

```
110-11011  1110211101 10100-1222 0100111111 10-4000130 1232200020 0320102012
1300001-11 0003131010 1?21011101 0201002100 21211123?1 0111001121 2002100-14
1110311101 0111101111 1110230110 1021112102 1110121011 010--111-0 012?0?00?0
11111?000- 0030111101 1101100001 0011111012 1110-21331 2113200012 010???????
?????????? ?????????? ?????????? ?????????? ?????????? ?????????? ?---11-110 2
```

Balaenoptera brydei
110-11011 1110211101 10100-1222 0100111111 10-4001120 1232200111 0320102012
1301--0-11 0003131010 1?21011101 0201002100 21211123?1 0111001121 0002101014
1110311001 0111101111 1110210110 1021112101 3110120111 000--111-0 212?0?00?0
11111?000- 0030111101 1101100001 0011111012 1100-21331 2113200012 0100000000
0021111101 1111112111 1112011211 1112010100 0111001111 1111111111 0---11-110 2

Balaenoptera edeni
110-11011 1110211101 10100-1212 0100111111 10-3000130 1232200111 0310102012
1300011-11 0003131010 1221011101 0201002100 21211123?1 0111001121 2102101014
1110311101 0111101111 1110210110 1021112101 3110120111 000--111-0 212?0?0010
11111?000- 0030111111 1101100001 0011111012 1100-21331 2113200012 0100000000
0020001101 1111112111 1112011211 1112010100 0111001111 1111111111 0---11-110 2

Balaenoptera borealis
110-11011 1110211101 10100-1222 0100111111 10-4000120 1232202110 0320102012
1301--1-11 0003131010 1221011101 0201102100 21211123?1 0111001121 2002100-14
1110311101 0111101111 1110210110 1021112102 1110121111 110--111-0 2121010010
111111100- 0130111101 1101100001 0011111012 1100-21301 2113200012 0100000000
0021111101 1111112111 1112011211 1122110100 0111001111 1111111111 0---11-110 2

Table S2

Results of phylogenetic analysis.

**Research algorithms**	**N[1]**	**TL[1]**	**CI[1]**	**RI[1]**
Traditional search	25	1606	0.291	0.756
Traditional search (weighted characters)	1	1638	0.285	0.749
**New Technology search (sectorial search set off; all algorithms)[2]**	**2**	**1604**	**0.291**	**0.756**
New Technology search (sectorial search set off; all algorithms; weighted characters)	4	1605	0.291	0.749
New Technology search (sectorial search set on; Tree Fusing)	1	1605	0.291	0.756
New Technology search (sectorial search set on; Tree Fusing; weighted characters)	2	1640	0.285	0.749
New Technology search (sectorial search set on; Drift)	2	1605	0.291	0.756
New Technology search (sectorial search set on; Drift; weighted characters)	2	1640	0.285	0.749
New Technology search (sectorial search set on; Ratchet)	1	1605	0.291	0.756
New Technology search (sectorial search set on; Ratchet; weighted characters)	1	1640	0.285	0.749

[1] Caption: N, number of cladograms found by the corresponding algorithms; TL, Tree length; CI, Consistency Index; RI, Retention Index.

[2]Bold: most parsimonious solutions used in the paper representing our preferred hypothesis of phylogeny.

Table S3

Dataset used for analysis of evolutionary radiation hypothesis. Numbers in the left column represent time intervals; numbers in the other columns represent numbers of rami observed in the cladogram in the corresponding time intervals.

**Time interval** (in Ma)	**Balaenomorpha**	**Balaenomorpha without Balaenopteridae**	**Balaenopteridae**	**Balaenoidaea** (from *Bisconti et al., 2017*)
0-1	14	6	9	5
1-2	14	6	9	5
2-3	23	6	17	5
3-4	33	8	25	11
4-5	21	9	12	9
5-6	29	14	15	11
6-7	21	6	15	5
7-8	40	16	24	9
8-9	39	16	23	3
9-10	32	16	16	3
10-11	32	16	16	3
11-12	49	22	27	4
12-13	17	11	6	1
13-14	21	21	0	1
14-15	14	14	0	1
15-16	12	12	0	1
16-17	21	21	0	2
17-18	11	11	0	2
18-19	9	9	0	2
19-20	9	9	0	2
20-21	14	14	0	0
21-22	5	5	0	0
22-23	1	1	0	0
23-24	6	6	0	0
24-25	6	6	0	0
25-26	8	8	0	0
26-27	8	8	0	0
27-28	8	8	0	0
28-29	10	10	0	0
29-30	10	10	0	0
30-31	10	10	0	0
31-32	10	10	0	0
32-33	10	10	0	0
33-34	11	11	0	0
34-35	17	17	0	0

Literature cited in the Supplementary Information

Andrews RC. 1914. Monograph of the Pacific Cetacea – The gray whale (*Rhachianectes glaucus* Cope). *Memoirs of the American Museum of Natural History* **1**:227–287.

Baker AN. 1985. Pygmy right whale *Caperea marginata* (Gray, 1846. In: Ridgway SM, Harrison R, eds. *Handbook of marine mammals*, Vol. 3. London: Academic Press, 345–354.

Barnes L, Kimura GM, Furusawa H, Sawamura H. 1995. Classification and distribution of Oligocene Aetiocetidae (Mammalia; Cetacea; Mysticeti) from western North America and Japan. *The Island Arc* **3**:392–431.

Beddard FE. 1901. Contribution towards a knowledge of the osteology of the pigmy whale (*Neobalaena marginata*). *Transactions of the Zoological Society* **16:**87–108.

Bisconti M. 2000. New description, character analysis and preliminary phyletic assessment of two Balaenidae skulls from the Italian Pliocene. *Palaeontographia Italica* **87:**37–66.

Bisconti M. 2003. Evolutionary history of Balaenidae. *Cranium* **20:**9–50.

Bisconti M. 2005. Morphology and phylogenetic relationships of a new diminutive balaenid from the lower Pliocene of Belgium. *Palaeontology* **48:**793–816.

Bisconti M. 2006. *Titanocetus*, a new baleen whale from the Middle Miocene of northern Italy (Mammalia, Cetacea, Mysticeti). *Journal of Vertebrate Paleontology* **26:**344–354.

Bisconti M. 2007a. A new basal balaenopterid from the Early Pliocene of northern Italy. *Palaeontology* **50:**1103–1122.

Bisconti M. 2007b. Taxonomic revision and phylogenetic relationships of the rorqual-like mysticete from the Pliocene of Mount Pulgnasco, northern Italy (Mammalia, Cetacea, Mysticeti). *Palaeontographia Italica* **91:**85–108.

Bisconti M. 2008. Morphology and phylogenetic relationships of a new eschrichtiid genus (Cetacea: Mysticeti) from the Early Pliocene of northern Italy. *Zoological Journal of the Linnean Society* **153:**161–186.

Bisconti M. 2010. A new balaenopterid whale from the Late Miocene of the Stirone River, northern Italy (Mammalia, Cetacea, Mysticeti). *Journal of Vertebrate Paleontology* **30:**943–958.

Bisconti M. 2011. New description of *'Megaptera' hubachi* Dathe, 1983 based on the holotype skeleton held in the Museum für Naturkunde, Berlin. In: Bisconti M, Roselli A, Borzatti de Loewenstern A, eds. *Climatic Change, Biodiversity, Evolution: Natural History Museum and Scientific Research. Proceedings of the Meeting. Quaderni del Museo di Storia Naturale di Livorno* **23:**37–68.

Bisconti M. 2012. Comparative osteology and phylogenetic relationships of *Miocaperea pulchra*, the first fossil pygmy right whale genus and species (Cetacea, Mysticeti, Neobalaenidae). *Zoological Journal of the Linnean Society* **166:**876–911.

Bisconti M. 2014. Anatomy of a new cetotheriid genus and species from the Miocene of Herentals, Belgium, and the phylogenetic and paleobiogeographic relationships of Cetotheriidae s.s. (Mammalia, Cetacea, Mysticeti). *Journal of Systematic Palaeontology* **13:**377–395.

Bisconti M, Bosselaers M. 2016. *Fragilicetus velponi*: a new mysticete genus and species and its implications for the origin of Balaenoperidae (Mammalia, Cetacea, Mysticeti). *Zoological Journal of the Linnean Society* **177:**450-474.

Bisconti M, Lambert O, Bosselaers M. 2013. Taxonomic revision of *Isocetus depawi* (Mammalia, Cetacea, Mysticeti) and the phylogenetic relationships of archaic 'cetothere' mysticetes. *Palaeontology* **56:**95–127.

Bisconti M, Varola A. 2006. The oldest eschrichtiid mysticete and a new morphological diagnosis of Eschrichtiidae. *Rivista Italiana di Paleontologia e Stratigrafia* **119:**447–457.

Boessenecker RW. 2013. A new marine vertebrate assemblage from the Late Neogene Purisima Formation in Central California, part II: Pinnipeds and Cetaceans. *Geodiversitas* **35:**815–940.

Boessenecker R, Fordyce RE. 2015. Anatomy, feeding ecology, and ontogeny of a transitional baleen whale: a new genus and species of Eomysticetidae (Mammalia: Cetacea) fromthe Oligocene of New Zealand. *PeerJ* **3**:e1129; DOI 10.7717/peerj.1129.

Bosselaers M, Post K. 2010. A new fossil rorqual (Mammalia, Cetacea, Balaenopteridae) from the Early Pliocene of the North Sea, with a review of the rorqual species described by Owen and Van Beneden. *Geodiversitas* **32:**331–363.

Bouetel V, De Muizon C. 2006. The anatomy and relationships of *Piscobalaena nana* (Cetacea, Mysticeti), a Cetotheriidae s.s. from the early Pliocene of Peru. *Geodiversitas* **28:**319–395.

Buono MR, Fernandez MS, Cozzuol MA, Cuitiño JI, Fitzgerald EMG. 2018. The early Miocene balaenid *Morenocetus parvus* from Patagonia (Argentina) and the evolution of right whales. *PeerJ* **5**:e4148

Burns JJ, Montague JJ, Cowles CJ, eds. 1993. The Bowhead whale. *The Society for Marine Mammalogy, Special Publication* **2:**1–787.

Caretto PG. 1970. La balenottera delle sabbie plioceniche di Valmontasca (Vigliano d'Asti). *Bollettino della Società Paleontologica Italiano* **9:**3–750.

Clapham PJ. 2002. Humpback whale (*Megaptera novaeangliae*). In: Perrin WF, Wursig B, Thewissen JGM, eds. *Encyclopedia of Marine Mammals*. London: Academic Press, 589–592.

Cummings WC. 1985a. Right whales – *Eubalaena glacialis and Eubalaena australis*. In: Ridgway SM, Harrison R, eds. *Handbook of marine mammals,* Vol. 3. London: Academic Press, 275–304.

Cummings WC. 1985b. Bryde's whale – *Balaenoptera edeni* Anderson, 1878. In: Ridgway SM, Harrison R, eds. *Handbook of marine mammals,* Vol. 3. London: Academic Press, 137–170.

Dathe F. 1983. *Megaptera hubachi* n. sp., ein fossiler Bartenwal aus marinen Sandsteinschichten des tieferen Pliozäns Chiles. *Zeitscrift Für Geological Wissenschaften* **11:**813–852.

Deméré TA, Berta A, McGowen MR. 2005. The taxonomic and evolutionary history of fossil and modern balaenopteroid mysticetes. *Journal of Mammalian Evolution* **12:**99–143.

El Adli JJ, Deméré TA, Boessenecker RW. 2014. *Herpetocetus morrowi* (Cetacea: Mysticeti), a new species of diminutive baleen whale from the Upper Pliocene (Piacenzian) of California, USA, with observations on the evolution and relationships of the Cetotheriidae. *Zoological Journal of the Linnean Society* **170:**400–466.

Fitzgerald EMG. 2010. The morphology and systematics of *Mammalodon colliveri* (Cetacea: Mysticeti), a toothed mysticete from the Oligocene of Australia. *Zoological Journal of the Linnean Society* **158:**367–476.

Fitzgerald EMG. 2006. A bizarre new toothed mysticete (Cetacea) from Australia and the early evolution of baleen whales. *Proceedings of the Royal Society B* **273:**2955–2963.

Fordyce RE, Marx FG. 2012. The pygmy right whale *Caperea marginata* – the last of the cetotheres. *Proceedings of the Royal Society, Series B* **280:**20122645.

Freschi A, Cau S. 2016. La riscoperta di Monte Pulgnasco: nuova collocazione cronostratigrafica e geografica di *Protororqualus cuvieri* (Cetacea: Misticeti, Balaenopteridae). *Quaderni del Museo Civico di Storia Naturale di Ferrara* **3:**21-30.

Gambell R. 1985a. Sei whale – *Balaenoptera borealis*. In: Ridgway SM, Harrison R, eds. *Handbook of marine mammals,* Vol. 3. London: Academic Press, 155–170.

Gambell R. 1985b. Fin whale – *Balaenoptera physalus*. In: Ridgway SM, Harrison R, eds. *Handbook of marine mammals,* Vol. 3. London: Academic Press, 171–192.

Geisler JH, Luo Z. 1998. Relationships of Cetacea to terrestrial Ungulates and the evolution of cranial vasculature in Cete. In: Thewissen JGM, ed. *The emergence of whales: evolutionary patterns in in the origin of Cetacea*. New York: Plenum Press, 163–212.

Geisler JH, Sanders AE. 2003. Morphological evidence for the phylogeny of Cetacea. *Journal of Mammalian Evolution* **10:**23–129.

Gingerich PD, ul-Haq M, von Koenigswald W, Sanders WJ, Holly Smith B, Zalmout IS. 2009. New Protocetid Whale from the Middle Eocene of Pakistan: Birth on Land, Precocial Development, and Sexual Dimorphism. *Plos One* **4**:e4366.
Gol'Din P, Startsev D, Krakhmalnaya T. 2014. The anatomy of *Cetotherium riabinini* Hofstein, 1948, a baleen whale from the late Miocene of Ukraine. *Acta Palaeontologica Polonica* **59:**795–814.
Gottfried MD, Bohaska DJ, Whitmore FC Jr (1994). Miocene cetaceans of the Chesapeake Group. *Proceedings of the San Diego Society of Natural History* **29:**229-238.
Govender R, Bisconti M, Chinsamy A. 2016. A late Miocene–early Pliocene baleen whale assemblage from Langebaanweg, west coast of South Africa (Mammalia, Cetacea, Mysticeti). *Alcheringa* **40:**542-555.
Hanna GD, McLellan ME. 1924. A new species of fin whale from the type locality of the Monterey Group. *Proceedings of the California Academy of Sciences* **13:**237–241.
Horwood J. 2002. Sei whale (*Balaenoptera borealis*). In: Perrin WF, Wursig B, Thewissen JGM, eds. *Encyclopedia of Marine Mammals*. London: Academic Press, 1069–1071.
Hulbert RC Jr. 1998. Postcranial osteology of the North American Middle Eocene protocetid *Georgiacetus*. In: Thewissen JGM, ed. *The emergence of whales*. New York: Plenum Press, 235–267.
Hulbert RC Jr, Petkewich RM, Bishop GA, Bukry D, Aleshire DP. 1996. A new Middle Eocene protocetid whale (Mammalia: Cetacea: Archaeoceti) and associated biota from Georgia. *Journal of Paleontology* **72:**907–927.
Jones ML, Schwartz SL. 2002. Gray whale (*Eschrichtius robustus*). In: Perrin WF, Wursig B, Thewissen JGM, eds. *Encyclopedia of Marine Mammals*. London: Academic Press, 524–537.
Kato H. 2002. Bryde's whales (*Balaenoptera edeni* and *B. brydei*). In: Perrin WF, Wursig B, Thewissen JGM, eds. *Encyclopedia of Marine Mammals*. London: Academic Press, 171–177.
Kellogg R. 1928. The history of whales – their adaptation to life in the water. *Quarterly Review of Biology* **3:**29–76; and 174–208.
Kellogg R. 1922. Description of the skull of *Megaptera miocaena*, a fossil humpback whale from the Miocene diatomaceous earth of Lompoc; California. *Proceedings of The United States National Museum* **61:**1–18.
Kellogg R. 1934a. The Patagonia fossil whalebone whale, *Cetotherium moreni* (Lydekker). *Contributions in Palaeontology, Carnegie Insitution Washington* **447:**63–81.
Kellogg R. 1934b. A new cetothere from the Modelo Formation at Los Angeles, California. *Contributions in Palaeontology, Carnegie Insitution Washington* **447:**85–104.
Kellogg R. 1936. A review of the Archaeoceti. *Carnegie Institution Washington* **482:**1–366.
Kellogg R. 1965. A new whalebone whale from the Miocene Calvert Formation. *United States National Museum Bulletin* **247:**1–45.
Kellogg R. 1968a. Miocene Calvert mysticetes described by Cope. *United States National Museum Bulletin* **247:**103–132.
Kellogg R. 1968b. A hitherto unrecognized Calvert mysticete. *United States National Museum Bulletin* **247:**133–161.
Kellogg R. 1968c. A sharp-nosed cetothere from the Miocene Calvert. *United States National Museum Bulletin* **247:**163–197.
Kellogg R. 1968d. Supplement to description of Parietobalaena palmeri. *United States National Museum Bulletin* **247:**175–197.
Kellogg R. 1969a. The skeleton of a Miocene Choptank cetothere. *United States National Museum Bulletin* **294:**1–24.
Kellogg R. 1969b. The vertebrae of a second Miocene Choptank cetothere. *United States National Museum Bulletin* **294:**24–40.

Kimura T, Hasegawa Y. 2010. A new baleen whale (Mysticeti: Cetotheriidae) from the earliest Late Miocene of Japan and a reconsideration of the phylogeny of cetotheres. *Journal of Vertebrate Paleontology* **30:**577–591.

Kimura T, Ozawa T. 2002. A new cetothere (Cetacea: Mysticeti) from the Early Miocene of Japan. *Journal of Vertebrate Paleontology* **22:**684–702.

Kimura T, Adaniya A, Oishi M, Marx FG, Hasegawa Y. 2015. A Late Miocene balaenopterid ("Shimajirikujira") from the Okamishima Formation, Shimajiri Group, Miyako Island, Okinawa, Japan. *Bulletin of the Gunma Museum of Natural History* **19:**39–48. (in Japanese with English abstract).

Luo Z, Gingerich PD. 1999. Terrestrial Mesonychia to aquatic Cetacea: transformation of the basicranium and evolution of hearing in whales. *University of Michigan Papers in Paleontology* **31:**1–98.

Martinez-Caceres M, Lambert O, Muizon C de. 2017. The anatomy and phylogenetic affinities of *Cynthiacetus peruvianus*, a large *Dorudon*-like basilosaurid (Cetacea, Mammalia) from the late Eocene of Peru. *Geodiversitas* **39**:1–163.

Marx FG, Tsai C-H, Fordyce RE. 2015. A new Early Oligocene toothed 'baleen'whale (Mysticeti: Aetiocetidae) from western North America: one of the oldest and the smallest. *Royal Society Open Science* **2**:150476.

Marx FG, Lambert O, Muizon C de. 2017. A new Miocene baleen whale from Peru deciphers the dawn of cetotheriids. *Royal Society Open Science* **4**:170560.

Marx GF, Kohno N. 2016. A new Miocene baleen whale from the Peruvian desert. *Royal Society Open Science* **3:**160542. http://dx.doi.org/10.1098/rsos.160542.

Miller GS. 1923. The telescoping of the cetacean skull. *Smithsonian Miscellaneous Collections* **76:**1–70.

Oishi M, Kawakami T, Hasegawa, Y. 1985. Pliocene baleen whales and bony-toothed bird from Iwate Prefecture, Japan (Parts I–VI). *Bulletin of the Iwate Prefectural Museum* **3:**143–157.

Okazaki Y. 2012. A new mysticete from the upper Oligocene Ashiya Group, Kyushu, Japan and its significance to mysticete evolution. *Bulletin of the Kitakyushu Museum of Natural History and Human History Series A (Natural History)* **10:**129–152.

Omura H. 1975. Osteological study of the minke whale from the Antarctic. *Scientific Reports of the Whales Research Institute* **27**:1–36.

Packard EL, Kellogg R. 1934. A new cetothere from the Miocene Astoria Formation of Newport, Oregon. *Publication of Carnegie Institution Washington* **447**:1–62.

Peredo CM, Uhen MD. 2016. A new basal chaeomysticete (Mammalia: Cetacea) from the late Oligocene Pysht Formation of Washington, USA. *Papers in Palaeontology* **2016**:1–22.

Perrin WR, Brownell RL Jr. 2002. Minke whales (*Balaenoptera acutorostrata* and *B. bonaerensis*). In: Perrin WF, Wursig B, Thewissen JGM, eds. *Encyclopedia of Marine Mammals*. London: Academic Press, 750-754.

Pilleri G. 1986. *Beobachtungen an den fossilen Cetaceen des Kaukasus*. Ostermundigen: Brain Anatomy Institute.

Pilleri G. 1989. *Balaenoptera siberi*, ein neuer Spätmiozäner Bartenwal aus der Pisco-Formation Perus. In: Pilleri G, ed. Beiträge zur Paläontologie der Cetaceen Perus. Ostermundigen: Hirnanatomisches Institut der Universitat Bern (Schweiz), 63-84.

Portis A. 1885. Catalogo descrittivo dei Talassoterii rinvenuti nei terreni terziari del Piemonte e della Liguria. *Memorie Della Reale Accademia Delle Scienze Di Torino* **37:**247–365.

Reeves RR, Leatherwood S. 1985. Bowhead whale – *Balaena mysticetus*. In: Ridgway SM, Harrison R, eds. *Handbook of marine mammals,* Vol. 3. London: Academic Press, 305–344.

Rugh DJ, Shelden KEW. 2002. Bowhead whale (*Balaena mysticetus*). In: Perrin WF, Wursig B, Thewissen JGM, eds. *Encyclopedia of Marine Mammals*. London, Academic Press, 12-131.

Sacco F. 1890. Sopra una mandibola di *Balaenoptera* dell'Astigiana. *Atti della Regia Accademia delle Scienze di Torino* **25:**3–8.

Sanders AE, Barnes LG. 2002a. Paleontology of the Late Oligocene Ashley and Chandler Bridge Formations of South Carolina, 2: *Mycromysticetus rothauseni*, a primitive cetotheriid mysticete (Mammalia: Cetacea). In: Emry RJ, ed. *Cenozoic mammals of land and sea: tributes to the career of Clayton E. Ray*. *Smithsonian Contributions in Paleobiology* **97:**271–293.

Sanders AE, Barnes LG. 2002b. Paleontology of the late Oligocene Ashley and Chandler Bridge formations of South Carolina, 3: Eomysticetidae, a new family of primitive mysticetes (Mammalia: Cetacea). In: Emry RJ, ed. *Cenozoic mammals of land and sea: tributes to the career of Clayton E. Ray*. *Smithsonian Contribution in Paleobiology* **93:**313–356.

Steeman ME. 2009. A new baleen whale from the Late Miocene of Denmark and early mysticete hearing. *Palaeontology* **52:**1169-1190.

Stewart BS, Leatherwood S. 1985. Minke whale – *Balaenoptera acutorostrata*. In: Ridgway SM, Harrison R, eds. *Handbook of marine mammals*, Vol. 3. London: Academic Press, 91–136.

Tanaka Y, Watanabe M. 2019. An early and new member of Balaenopteridae from the upper Miocene of Hokkaido, Japan. *Journal of Systematic Palaeontology* DOI: 10.1080/ 14772019.2018.1532968.

True FW. 1904. The whalebone whales of the western north Atlantic, compared with those occurring in European waters; with some observations on the species of the north Pacific.
Smithsonian Contributions to Knowledge **33:**1–332.

Tsai C-H, Fordyce RE. 2015. The Earliest Gulp-Feeding Mysticete (Cetacea: Mysticeti) from the Oligocene of New Zealand. *Journal of Mammalian Evolution* DOI 10.1007/s10914-015-9290-0.

Uhen MD. 1998. Middle to Late Eocene basilosaurines and dorudontines. In: Thewissen JGM, ed. *The emergence of whales: evolutionary patterns in in the origin of Cetacea*. New York: Plenum Press, 29–63.

Uhen MD. 2004. Form, function, and anatomy of *Dorudon atrox* (Mammalia, Cetacea): an archaeocete from the middle to late Eocene of Egypt. *University Michigan Paper in Paleontology* **34:**1–222.

Wada S, Oishi M, Yamada TK. 2003. A newly discovered species of living baleen whale. *Nature* **426:**278–281.

Winn HE, Reichley NE. 1985. Humpback whale – *Megaptera novaeangliae*. In: Ridgway SM, Harrison R, eds. *Handbook of marine mammals*, Vol. 3. London: Academic Press, 241–274.

Yamada TK, Kakuda T, Tajima Y. 2008. Middle sized balaenopterid whale specimens in
the Philippines and Indonesia. *Memoirs of the National Museum of Natural Sciences, Tokyo* **45**:75–83.

Yochem PK, Leatherwood S. 1985. Bowhead whale – *Balaena mysticetus*. In: Ridgway SM, Harrison R, eds. *Handbook of marine mammals*, Vol. 3. London: Academic Press, 305–354.

Zeigler CV, Chan GL, Barnes LG, 1997. A new Late Miocene balaenopterid whale (Cetacea: Mysticeti), *Parabalaenoptera baulinensis*, (new genus and species) from the Santa Cruz Mudstone, Point Reyes Peninsula, California. *Proceedings of the California Academy of Sciences* **50**:115–138.
